# Tumor-targeting nanocarriers amplified immunotherapy of cold tumors by STING activation and inhibiting immune evasion

**DOI:** 10.1126/sciadv.adr1728

**Published:** 2025-06-27

**Authors:** Jinhua Zhao, Aiping Tong, Jing Liu, Mingxia Xu, Peng Mi

**Affiliations:** ^1^ Department of Radiology, Huaxi MR Research Center (HMRRC), and State Key Laboratory of Biotherapy, West China Hospital, Sichuan University, Chengdu, Sichuan 610041, China.; ^2^ Department of Biotherapy and State Key Laboratory of Biotherapy, West China Hospital, Sichuan University, Chengdu, 610041, China.

## Abstract

The low immunogenicity and immune escape are bottlenecks for effective tumor immunotherapy. Here, we synthesized multifunctional polymers comprising a photosensitizer and cationic and thiol derivates and engineered a galactose-installed stimulator of interferon genes (STING) agonist and programmed death ligand 1 (PD-L1) small interfering RNA (siPDL1)–encapsulated nanocarriers (cGAMP-siPDL1@GalNPs) for synergistic immunotherapy of low immunogenic tumors through stimulating robust immune responses. cGAMP-siPDL1@GalNPs efficiently delivered the drugs into cancer cells by targeting the galactose receptors to trigger photo-/redox-/pH-activated drug release. cGAMP-siPDL1@GalNPs stimulated robust antitumor immunity via STING activation and immunogenic cell death (ICD) and inhibited immune escape via knockdown of PD-L1 expression in tumors, which synergistically regulated the immune-suppressive tumor microenvironment. Upon laser irradiation, the nanocarriers efficiently eradicated primary melanoma and orthotopic triple-negative breast tumors and induced ICD effects, which synergically inhibited the distant tumor and spontaneous lung metastasis with improved survival rates. This study presents a strategy for developing nanocarriers to activate antitumor immunity and regulate immune invasion for effective immunotherapy.

## Teaser

Tumor-targeting nanocarriers deliver immunotherapeutics to stimulate robust immune responses and inhibit immune evasion.

## INTRODUCTION

Immunotherapy emerged as an important therapeutic strategy for treating tumors, following the clinical application of immune checkpoint inhibitors (*1*). However, only a very low rate of tumors is sensitive to the immune checkpoint inhibitors due to their heterogenetic nature and low immunogenic tumor microenvironment (TME) (*2*, *3*). Thus, boosting robust antitumor immune responses is essential for successful immunotherapy, especially for poorly immunogenic tumors. Recently, the stimulator of interferon genes (STING) pathway was found to be critical to tumor immunotherapy (*4*–*6*); STING is an endoplasmic reticulum (ER) protein that is expressed in both cancer cells and immune cells (*7*), which can be activated by STING agonists (*8*, *9*). After translocating from ER to the Golgi apparatus, STING activates TANK-binding kinase 1 (TBK1) and IκB kinase (*10*, *11*), which in turn activate the transcription factors interferon regulatory factor 3 (IRF3) and nuclear factor κB, respectively (*12*, *13*). These transcription factors then enter the nucleus to induce the expression of type I interferon (IFN-I) and other cytokines, such as tumor necrosis factor (TNF), interleukin-1β (IL-1β), and IL-6, which are capable of directly eradicating cancer cells or indirectly promoting antitumor immunity (*14*). Of note, STING activation in several cell types can benefit cancer immunotherapy. Activation of STING in antigen-presenting cells (APCs) such as dendritic cells (DCs) triggers DC maturation, promotes cross-talk between APCs and cytotoxic T lymphocytes (CTLs), and potentiates antitumor T cell responses (*15*, *16*). STING agonists can reprogram M2-type macrophages into M1-type antitumor macrophages (*17*). Moreover, selective activation of STING in cancer cells can produce chemokines and cytokines for recruiting tumor-infiltrating lymphocytes, priming CTLs, and sensitizing originally “cold” (low immunogenicity) tumors to immune checkpoint blockade therapy (*18*). Furthermore, activation of STING in tumor endothelial cells can disrupt abnormal vascular patterns to increase drug accumulation in tumors (*19*). In addition, several chemical compounds have been investigated as STING activators (*20*–*22*), such as the endogenous and high-affinity ligand 2′,3′-cyclic guanosine monophosphate–adenosine monophosphate (cGAMP) (*23*). However, they are generally limited by barriers of easy degradation, low cellular uptake and intracellular trafficking, and unsatisfied accumulation in tumors (*23*–*25*), which together lead to poor antitumor immunity and potential side effects (*25*). Therefore, developing nanocarriers to deliver STING agonists to tumors is critical for selectively activating the STING pathway, boosting robust innate immune responses, and reducing potential side effects (*26*).

While STING agonists stimulate the antitumor immune factors, such as cytotoxic CD8^+^ T cells, they also up-regulate the expression of programmed death ligand 1 (PD-L1) in cancer cells (*27*–*31*), which inevitably attenuates the effect of the activated CD8^+^ T cells and causes immune escape of cancer cells. Therefore, combining STING agonists with PD-L1 blockade is necessary for effective immunotherapy. Although anti–PD-L1 antibody (aPD-L1) is typically administrated for cancer immunotherapy, aPD-L1 usually suffers from poor diffusion, low tumor penetration, and immune-related adverse events, including fatal myocarditis, colitis, vitiligo, etc. (*32*, *33*). Besides, aPD-L1 only blocks PD-L1 on cell membranes, which could be compensated by the constant expression of proteins by cancer cells (*34*). Thus, the knockdown of PD-L1 by RNA interference (RNAi) is an alternative practical strategy. aPD-L1 is only sensitive to a small rate of “hot” tumors and invalid to treat cold tumors, which requires additional approaches (e.g., STING agonists) to activate the antitumor immune responses (*35*). However, a combination of RNAi and STING activation has not been sufficiently studied. In addition, the small interfering RNAs (siRNAs) are sensitive to ribonuclease and can neither enter cancer cells nor accumulate in tumors; therefore, the development of a siRNA delivery strategy involving reliable gene carriers is required for combined cancer immunotherapy (*36*, *37*). Polymeric nanocarriers are generally self-assembled from block copolymers and demonstrate a high potential for delivering bioactive compounds, such as siRNAs and anticancer drugs, which improve their therapeutic efficacy and reduce side effects (*38*, *39*). The successful clinical translation of some polymeric nanocarriers, such as Nanoxel and Genexol-PM, has promoted further development of polymeric nanocarrier therapeutics (*40*). Specifically, polymeric nanocarriers with cationic segments can interact with negatively charged nucleic acids and deliver them into cancer cells via endosome escape to achieve the desired effects of gene expression or protein knockdown effects (*41*, *42*). However, most ionic complexes are unstable under physiological conditions, requiring further optimization (*43*). Alternative strategies have been used to improve the efficacy of gene and drug delivery, including installing ligands on the nanocarrier surfaces for active targeting (*44*), engineering stimuli-responsive functions for desired cargo release (*45*), and laser irradiation-mediated endosome escape (*46*–*50*). Concerning the stable encapsulation and delivery of genes and drugs, efficiently delivering them into the target cells and achieving the desired immunotherapeutic effects remain major challenges (*51*). Therefore, developing polymeric nanocarriers for nucleotides or nucleic acid delivery is required in cancer immunotherapy.

**Fig. 1.**
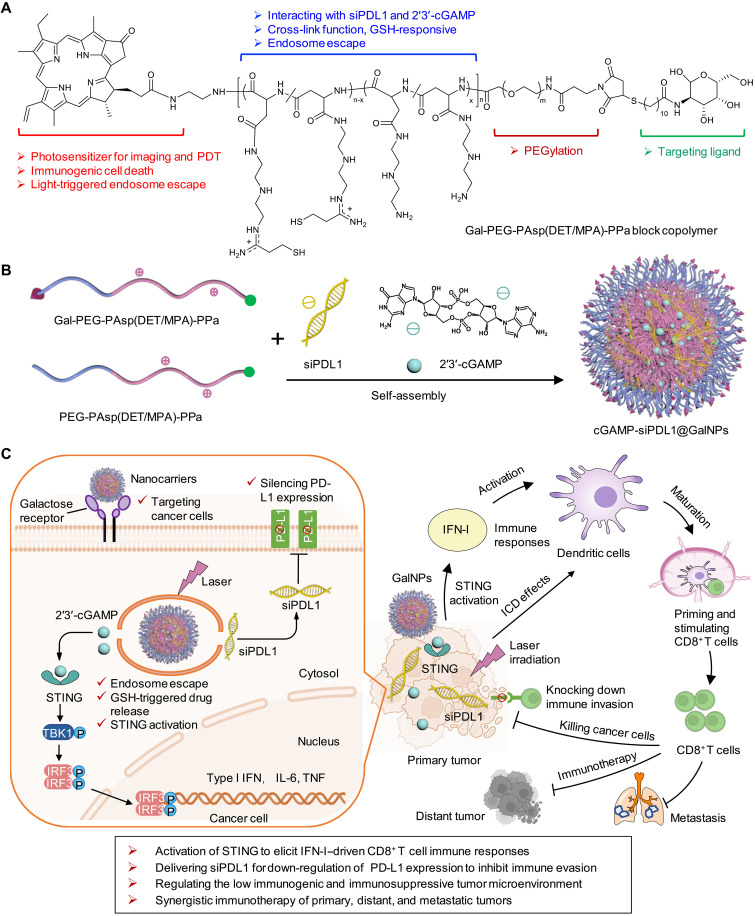
Engineering multiple-responsive polymeric nanocarrier for synergistically amplified immunotherapy of low immunogenic tumors by STING activation and inhibiting immune evasion. (**A**) Chemical structure and multiple functions of the synthesized Gal-PEG-PAsp(DET/MPA)-PPa block copolymer. (**B**) Self-assembly of cGAMP-siPDL1@GalNPs. (**C**) cGAMP-siPDL1@GalNPs actively target cancer cells by interacting with galactose receptors to intracellularly deliver cGAMP and siPDL1, which in turn synergistically stimulate robust antitumor immune responses and inhibit immune evasion through STING activation, ICD effects, and silencing PD-L1 expression for immunotherapy of primary, distant, and metastatic low immunogenic tumors.

Here, we have synthesized multiple functional triblock copolymers comprising a photosensitizer and cationic and thiol side chains and engineered galactose-installed, STING agonist cGAMP and PD-L1 siRNA (siPDL1)–encapsulated polymeric nanoparticles (cGAMP-siPDL1@GalNPs), which synergistically stimulated robust immune responses and inhibited immune evasion by tumor-specific activation of STING, immunogenic cell death (ICD) effects, and silencing PD-L1 expression, for amplified immunotherapy of low immunogenic tumors (Fig. 1, A and B). The nanocarriers were engineered by self-assembly with disulfide cross-linked cores and can intensively target cancer cells via galactose receptors for efficient cytosolic delivery of drugs through endosome escape by the low pH-activated cationic polymer and the photochemical internalization (PCI) of the photosensitizer (Fig. 1C). Redox potential-triggered cGAMP release activated the STING pathway to generate several immune effector factors, such as IFN-γ–secreting CD8^+^ T cells (IFN-γ^+^CD8^+^ T cells), cytokines, etc., while the siPDL1 knocked down PD-L1 expression on the cancer cells, and the photosensitizer allowed the use of tumor photodynamic therapy (PDT) to induce ICD of the cancer cells. These synergistic immunotherapeutic effects effectively eradicate primary, distant, and metastatic tumors, as well as inhibit immune escape (Fig. 1C). This study presents the synthesis and functional analysis of a multifunctional nanocarrier that efficiently delivers immunotherapeutic agents to tumors, thus synergistically activating antitumor immunity and inhibiting immune escape to enable effective immunotherapy of poorly immunogenic tumors.

## RESULTS

### Synthesis of the photosensitizer and targeting ligand-caped triblock copolymer

To efficiently deliver siPDL1 and cGAMP, we synthesized the multifunctional triblock copolymer Gal-PEG–poly{N-[N′-(2-aminoethyl)-2-aminoethyl]aspartamide modified with 1-(3-mercaptopropyl)amidine}–PPa comprising a photosensitizer and a targeting ligand at either end, cationic units, sulfide-containing cationic units, and a poly(ethylene glycol) (PEG) segment, named Gal-PEG-PAsp(DET/MPA). Briefly, we first synthesized the photosensitizer initiator (3S,4S)-*N*-(2-aminoethyl)-9-ethenyl-14-ethyl-4,8,13,18-tetramethyl-20-oxo-3-phorbinepropanamide (PPa-NH_2_), and the thiol-galactose (Gal-SH) was synthesized (fig. S1, A and B). Next, PPa-NH_2_ initiated the ring-opening polymerization of benzyl-l-aspartate N-carboxy anhydride to form PPa–poly(β-benzyl l-aspartate) (PPa-PBLA), which was then reacted with maleimide–PEG–succinimidyl ester and Gal-SH subsequently to obtain Gal-PEG-PBLA-PPa. Next, the side chains were aminiolyzed with diethylenetriamine to obtain cationic amine groups (*52*, *53*), which was partially (15%) reacted with dimethyl 3,3′-dithiobispropionimidate to obtain Gal-PEG-PAsp(DET/MPA)-PPa with sulfide cationic side chain units (fig. S1C) (*54*, *55*). A similar protocol was followed to synthesize the ligand-free triblock copolymer PEG-PAsp(DET/MPA)-PPa. The chemical structures of the above-mentioned products were confirmed by ^1^H nuclear magnetic resonance (^1^H NMR) spectroscopy (figs. S2 to S9).

### Engineering of the photo-/redox-/pH-activatable nanocarriers

**Fig. 2.**
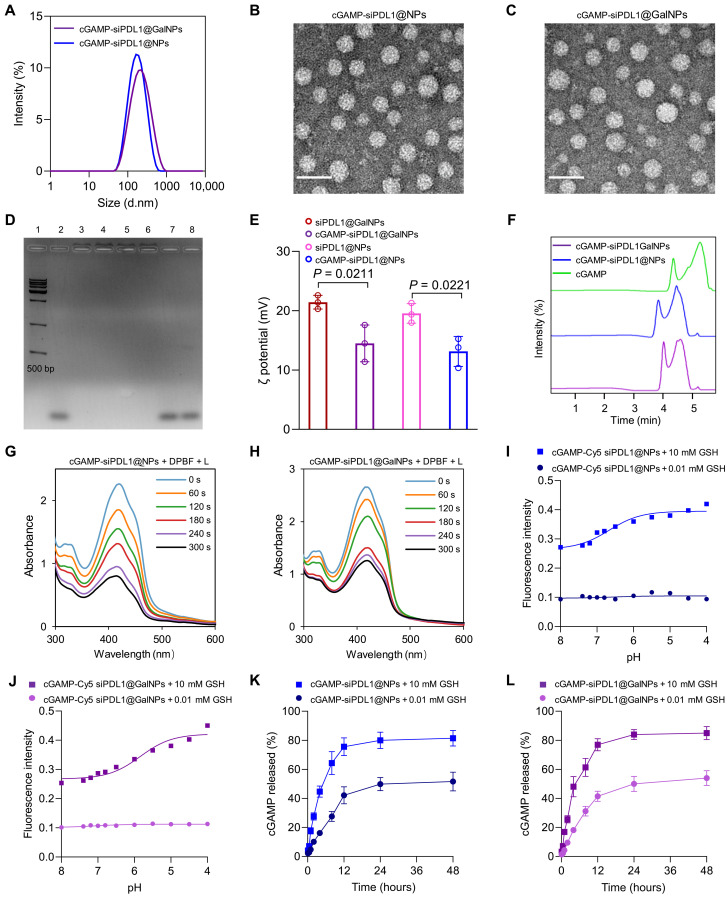
Preparation and characterization of the multifunctional cGAMP-siPDL1@GalNPs. (**A** to **C**) DLS results and TEM images show the diameter (A) and morphology (B and C) of cGAMP-siPDL1@NPs and cGAMP-siPDL1@GalNPs. Scale bars, 100 nm. (**D**) Agarose gel electrophoresis of the siPDL1-loaded nanocarriers with/without adding DTT and heparin shows the stability of the cGAMP-siPDL1@NPs and cGAMP-siPDL1@GalNPs nanocarriers and the DTT-trigged release of siPDL1 (1: DNA marker; 2: siPDL1; 3: cGAMP-siPDL1@NPs; 4: cGAMP-siPDL1@GalNPs; 5: cGAMP-siPDL1@NPs + heparin; 6: cGAMP-siPDL1@GalNPs + heparin; 7: cGAMP-siPDL1@NPs + DTT + heparin; 8: cGAMP-siPDL1@GalNPs + DTT + heparin). (**E**) Zeta potential values of siPDL1@NPs, cGAMP-iPDL1@NPs, siPDL1@GalNPs, and cGAMP-siPDL1@GalNPs. (**F**) The HPLC chromatographic spectrum of free cGAMP and cGAMP extracted from cGAMP-siPDL1@NPs and cGAMP-siPDL1@GalNPs. (**G** and **H**) ROS (i.e., ^1^O_2_) generation upon the laser irradiation of cGAMP-siPDL1@NPs (G) and cGAMP-siPDL1@GalNPs (H), which was monitored by measuring the UV-vis absorption spectra of DPBF. (**I** and **J**) Normalized fluorescence intensity as a function of the GSH and pH for cGAMP-Cy5 siPDL1@NPs (I) and cGAMP-Cy5 siPDL1@GalNPs (J). The lines are the simulated trend lines for the fluorescence intensity of the nanocarriers. (**K** and **L**) Release profiles of cGAMP released from cGAMP-siPDL1@NPs (K) and cGAMP-siPDL1@GalNPs (L) at 10 and 0.01 mM GSH conditions. The data are presented as the mean ± SD (*n* = 3). Statistical significance was determined using two-tailed unpaired *t* tests.

We first prepared nanocarriers cGAMP-siLuc@GalNPs by using firefly luciferase siRNA (siLuc), cGAMP, PEG-PAsp(DET/MPA)-PPa, and Gal-PEG-PAsp(DET/MPA)-PPa with various N/P molar ratios to evaluate the siRNA transfection effects. After transfection, cGAMP-siLuc@GalNPs with an N/P ratio of 20:1 obtained the lowest luminescence intensity in B16F10-Luc cells than nanocarriers with N/P ratios of 5:1 and 10:1, indicating the best RNAi effects at the N/P ratio of 20 (fig. S10). Therefore, nanocarriers were formulated at the N/P ratio of 20:1 for further study. Ligand density is a key factor for optimizing the targeting capability of nanocarriers (*44*, *56*). Recent studies have shown that nanocarriers with 25% PEG installed with glucose for targeting glucose transporter-1 (GLUT-1) in cancer cells can overcome the liver barrier and achieve higher accumulation in tumors and better antitumor activity (*57*). Considering galactose has the same molecular weight as glucose and can also target GLUT-1 (*58*), thus, the cGAMP-siPDL1@GalNPs polymeric nanocarriers were self-assembled by mixing the PEG-PAsp(DET/MPA)-PPa and Gal-PEG-PAsp(DET/MPA)-PPa cationic polymers in a 3:1 ratio along with siPDL1 and cGAMP (N/P molar ratio = 20:1) through electrostatic interactions and cross-linking the core with disulfide bonds (Fig. 1, A and B). Control nanocarriers comprising a negative control siRNA (siNC), siPDL1-incorporated polymeric nanocarriers with/without targeting ligand (galactose), namely, siPDL1@NPs, siPDL1@GalNPs, cGAMP-siNC@GalNP, cGAMP-siNC@NP, and cGAMP-siPDL1@NPs, were engineered in a similar manner. Dynamic light scattering (DLS) revealed similar hydrodynamic diameters for the nanocarriers: 135.6 and 145.6 nm for cGAMP-siPDL1@NPs and cGAMP-siPDL1@GalNPs, respectively (Fig. 2A). The average diameters of the control nanocarriers were ~145 nm (fig. S11). Transmission electron microscopy (TEM) revealed the nanocarriers to be spherical and homogeneous (Fig. 2, B and C).

The stabilities of the nanocarriers were first characterized by gel electrophoresis. Both cGAMP-siPDL1@NPs and cGAMP-siPDL1@GalNPs were stable and did not release siPDL1 (Fig. 2D). However, siPDL1 was completely released when the nanocarriers were exposed to heparin and dithiothreitol (DTT), which interacted with the polycationic polymers and cleaved the disulfide bonds in the nanocarriers. Therefore, the synthesized triblock copolymers could stably encapsulated siPDL1 within the nanocarriers, which responded to redox potential changes for cargo release. In another test that assessed the sizes and polydispersity indices (PDIs) of the nanocarriers under physiological conditions (RPMI 1640 medium containing 10% fetal bovine serum) over 7 days, the nanocarriers also demonstrated relatively high stabilities (fig. S12). All nanocarriers demonstrated slightly positive zeta potential values, with a slightly lower zeta potential for cGAMP-siPDL1@GalNPs compared with that of siPDL1@GalNPs due to the additional encapsulation of the negatively charged cGAMP (Fig. 2E). Besides, the results of the high-performance liquid chromatography (HPLC) chromatographic spectrum exhibited that the cGAMP was successfully incorporated inside the cGAMP-siPDL1@NPs and cGAMP-siPDL1@GalNPs (Fig. 2F). Notably, cGAMP presents a double peak in HPLC (Fig. 2F), because cGAMP exists in the form of disodium salt, which may be easily affected by the pH of the mobile phase. Besides, the encapsulation efficiency (EE) of siPDL1 was 98.8% as detected by the RiboGreen assay (fig. S13A), and the EE of cGAMP was 36.2% as analyzed by HPLC (fig. S13B). In addition, the 1,3-diphenyl-isobenzofuran (DPBF) was applied to detect the singlet oxygen (i.e., ^1^O_2_) generated by cGAMP-siPDL1@NPs and cGAMP-siPDL1@GalNPs upon laser irradiation while monitoring their ultraviolet-visible (UV-vis) absorbance spectra (*59*). A decrease in the absorption spectra of DPBF at 420 nm was only observed for laser-irradiated nanocarriers, indicating the generation of the reactive oxygen species (ROS) ^1^O_2_, which can be used for tumor PDT (Fig. 2, G and H, and fig. S14). As shown in Fig. 2 (I and J), both cGAMP–Cyanine 5 (Cy5) siPDL1@NPs and cGAMP-Cy5 siPDL1@GalNPs were stable at 0.01 mM glutathione (GSH) concentration with pH 4 to 8, as the fluorescence intensity of Cy5-labeled siPDL1 (Cy5 siPDL1) in those nanocarriers almost did not change. However, the nanocarriers degraded at 10 mM GSH as the fluorescence intensity of Cy5 siPDL1 in both cGAMP-siPDL1@NPs and cGAMP-siPDL1@GalNPs increased at lower pH values. Moreover, much more cGAMP were released at 10 mM GSH than at 0.01 mM GSH within 48 hours (Fig. 1, K and L). These results indicate that cGAMP-siPDL1@NPs and cGAMP-siPDL1@GalNPs first cleave the disulfide bond by GSH and then release the drugs in response to the pH response. Both nanoparticles therefore have both redox- and pH-responsive properties.

### Cellular uptake and in vitro photoactivity of cGAMP-siPDL1@GalNPs

**Fig. 3.**
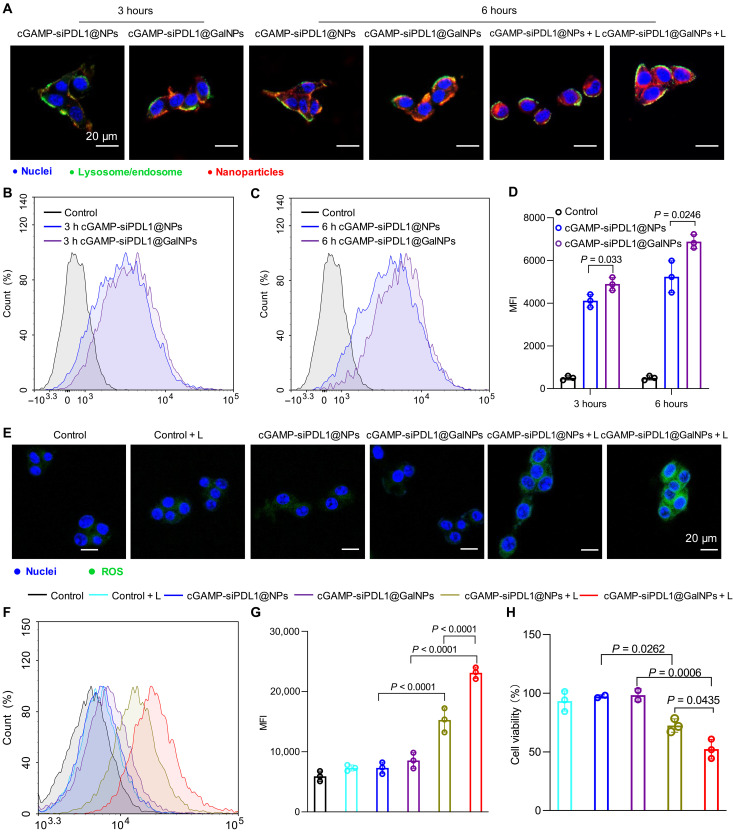
Intracellular drug delivery by cGAMP-siPDL1@GalNPs. (**A**) CLSM images of the B16F10 cancer cells at 3 and 6 hours after being exposed to cGAMP-siPDL1@NPs and cGAMP-siPDL1@GalNPs with/without laser irradiation. The nuclei are shown in blue, lysosomes in green, and nanocarriers in red. (**B** to **D**) Cellular uptake of the nanocarriers by the B16F10 cancer cells at 3 (B) and 6 hours (C) measured using a flow cytometer and (D) the calculated mean fluorescent intensity (MFI). (**E**) CLSM images show the ROS produced by cGAMP-siPDL1@NPs and cGAMP-siPDL1@GalNPs in B16F10 cells with/without laser irradiation. The nuclei and ROS are shown in blue and green, respectively. (**F** and **G**) FCM analysis (F) and MFI (G) of the ROS generated by the cGAMP-siPDL1@NPs and cGAMP-siPDL1@GalNPs nanocarriers in B16F10 cancer cells with/without laser irradiation. DCFH-DA was used as the ROS probe. (**H**) The cell viability of B16F10 cells after exposure to cGAMP-siPDL1@NPs and cGAMP-siPDL1@GalNPs for 6 hours with/without laser irradiation. Data are expressed as means ± SD (*n* = 3). Statistical analysis was performed using one-way analysis of variance (ANOVA) with Tukey test. Scale bars, 20 μm. h, hours.

Cancer cells overexpress galactose receptors, such as GLUT-1 (*44*, *60*, *61*), which could be targeted by the glycosylated nanocarriers to increase their accumulation in tumors and improve the therapeutic outcomes (*62*, *63*). We confirmed that GLUT-1 is overexpressed in both B16F10 melanoma and 4T1 breast tumors (see fig. S15 for a representative image). To verify the cellular uptake of the nanocarriers, cGAMP-siPDL1@GalNPs and cGAMP-siPDL1@NPs were exposed to B16F10 cancer cells, which were subjected to laser irradiation after 6 hours to test the effects of PCI-mediated intracellular delivery of nanocarriers. As demonstrated by confocal laser scanning microscopy (CLSM), cGAMP-siPDL1@GalNPs exhibited higher fluorescent intensities than cGAMP-siPDL1@NPs, demonstrating a higher cellular uptake of cGAMP-siPDL1@GalNPs by cancer cells (Fig. 3A and fig. S16). Notably, the endosomal/lysosomal escape rate of the cGAMP-siPDL1@GalNPs + L group was much higher than that of the cGAMP-siPDL1@NPs + L and cGAMP-siPDL1@NPs groups by quantifying the CLSM images with the Zeiss LSM880 fluorescence microscope (fig. S17). Alternatively, both nanocarriers exhibited more intracellular delivery upon laser irradiation due to the PCI-promoted endosome/lysosome escape. The cellular uptake of the nanocarriers was also verified by flow cytometry (FCM) (Fig. 3, B to D), which showed the same higher cellular uptake efficacy of cGAMP-siPDL1@GalNPs compared with cGAMP-siPDL1@NPs at both 3 and 6 hours. Thus, the addition of galactose to the nanocarriers promoted intracellular drug delivery. To further confirm the intracellular delivery mediated by GLUT-1, the cancer cells were firstly exposed to phloretin to block GLUT-1 and then exposed to the polymeric nanocarriers before evaluating their cellular uptake by CLSM and FCM (*62*, *64*). As shown in fig. S18 (A and B), the cGAMP-siPDL1@GalNPs had a cellular uptake similar to that of cGAMP-siPDL1@NPs when the GlUT-1 was blocked by phloretin, but cGAMP-siPDL1@GalNPs demonstrated a higher rate of cellular uptake in the absence of phloretin. Meanwhile, CLSM also revealed similar fluorescence intensities for cGAMP-siPDL1@GalNPs and cGAMP-siPDL1@NPs in cancer cells with phloretin (fig. S18C). These results showed that the specific internalization between the galactose ligands and GLUT-1 promoted the cellular uptake of cGAMP-siPDL1@GalNPs.

Next, the generation of ROS inside the cancer cells by the nanocarriers was detected using 2′,7′-dichlorodihydrofluorescein diacetate (DCFH-DA), which oxidizes to fluorescent 2′,7′-dichlorofluorescein (DCF) (*65*). The ROS-triggered fluorescence in B16F10 cancer cells was investigated by CLSM and FCM. As shown in Fig. 3E and fig. S19, the strongest DCF fluorescence signal upon laser irradiation was detected in cancer cells treated with cGAMP-siPDL1@GalNPs and laser irradiation, demonstrating that it produced the highest rate of ROS, which could in turn be explained by the highest cellular uptake of cGAMP-siPDL1@GalNPs. However, fewer ROS were generated by cGAMP-siPDL1@NPs and the control samples upon laser irradiation, with almost no ROS generation in the nonirradiated samples. Further FCM evaluation verified that ROS generation was higher in cGAMP-siPDL1@GalNPs compared with that of cGAMP-siPDL1@NPs inside the cancer cells after laser irradiation, indicated by higher mean fluorescent intensity (MFI) values (Fig. 3, F and G). Here, the ROS were generated by the photosensitizer PPa in the nanocarriers, which facilitated cancer cells death and the PDT of tumors (*66*–*68*). Next, the cell viability was tested by exposing the B16F10 cancer cells to nanocarriers and treating them with/without laser irradiation. As shown in Fig. 3H, the cGAMP-siPDL1@NPs and cGAMP-siPDL1@GalNPs obviously induced obvious cancer cell phototoxicity upon laser irradiation (660 nm, 100 mW/cm^2^, 5 min). Therefore, both cGAMP-siPDL1@NPs and cGAMP-siPDL1@GalNPs could generate ROS inside the cancer cells and kill B16F10 cancer cells by PDT.

### cGAMP-siPDL1@GalNPs-induced ICD effect

**Fig. 4.**
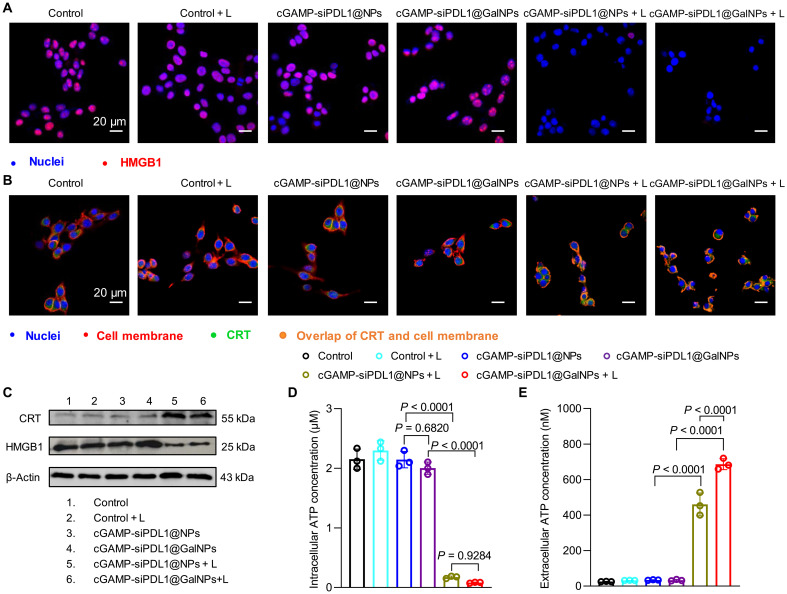
The cGAMP-siPDL1@GalNPs-induced ICD of cancer cells. (**A** and **B**) IF images of HMGB1 (A) and CRT (B) in B16F10 cancer cells after being treated with cGAMP-siPDL1@NPs and cGAMP-siPDL1@GalNPs for 6 hours and followed with/without laser irradiation (660 nm, 100 mW/cm^2^, 5 min). (**C**) WB assay results show the generation of HMGB1 and CRT in B16F10 cancer cells following treatments with cGAMP-siPDL1@NPs and cGAMP-siPDL1@GalNPs for 6 hours and followed with/without laser irradiation (660 nm, 100 mW/cm^2^, 5 min). (**D** and **E**) Intracellular (D) and extracellular (E) ATP levels in B16F10 cells when treated with cGAMP-siPDL1@NPs and cGAMP-siPDL1@GalNPs for 6 hours with/without laser irradiation (660 nm, 100 mW/cm^2^, 5 min). Data are expressed as means ± SD (*n* = 3). Statistical analysis was performed by one-way ANOVA with Tukey test.

Recent studies have shown that PDT not only kills tumor cells but also triggers the ICD effect (*69*). To assess the cGAMP-siPDL1@GalNPs-induced ICD, the cell surface expression of calreticulin (CRT), high mobility group protein 1 (HMGB1) release, and adenosine triphosphate (ATP) secretion were examined on B16F10 cells. Immunofluorescence (IF) staining was used to observe the HMGB1 and CRT distribution. As shown in Fig. 4A and fig. S20, the intracellular HMGB1 fluorescence signals of the nanocarriers in the laser irradiation group (referred to as cGAMP-siPDL1@NPs + L and cGAMP-siPDL1@GalNPs + L) were notably weaker than those of the nonlaser irradiation group (referring to control, control + L, cGAMP-siPDL1@NPs, and cGAMP-siPDL1@GalNPs). A large-scale overlap of the CRT and the B16F10 cell membranes was observed in the irradiated samples (orange area), while CRT was essentially intracellular in the absence of irradiation (Fig. 4B and fig. S21). These results demonstrated that cGAMP-siPDL1@NPs and cGAMP-siPDL1@GalNPs notably induced the extracellular release of HMGB1 and the translocation of CRT to the cell surface when irradiated. Consistent with the IF staining results, Western blot (WB) analyses also indicated the release of HMGB1 to the extracellular environment and translocation of CRT to the cell surface in B16F10 cells treated with cGAMP-siPDL1@GalNPs + L (Fig. 4C). Furthermore, extracellular and intracellular ATP levels were examined using the ATP assay kit. As shown in Fig. 4D, the intracellular ATP levels in the control groups (control, control + L, cGAMP-siPDL1@NPs, and cGAMP-siPDL1@GalNPs group) were significantly higher than those of cGAMP-siPDL1@NPs and cGAMP-siPDL1@GalNPs treated with laser irradiation (cGAMP-siPDL1@NPs + L and cGAMP-siPDL1@GalNPs + L groups). The intracellular ATP level in the cGAMP-siPDL1@GalNPs + L group was 18.55-fold higher than that of the cGAMP-siPDL1@GalNPs (Fig. 4E). These results show that laser-irradiated cGAMP-siPDL1@NPs and cGAMP-siPDL1@GalNPs significantly enhanced ATP secretion from B16F10 cells. In summary, cGAMP-siPDL1@GalNPs–based PDT could efficiently induce the ICD of tumor cells while retaining the potential to evoke an antitumor immune response.

### cGAMP-siPDL1@GalNPs activate STING and down-regulated PD-L1 expression

**Fig. 5.**
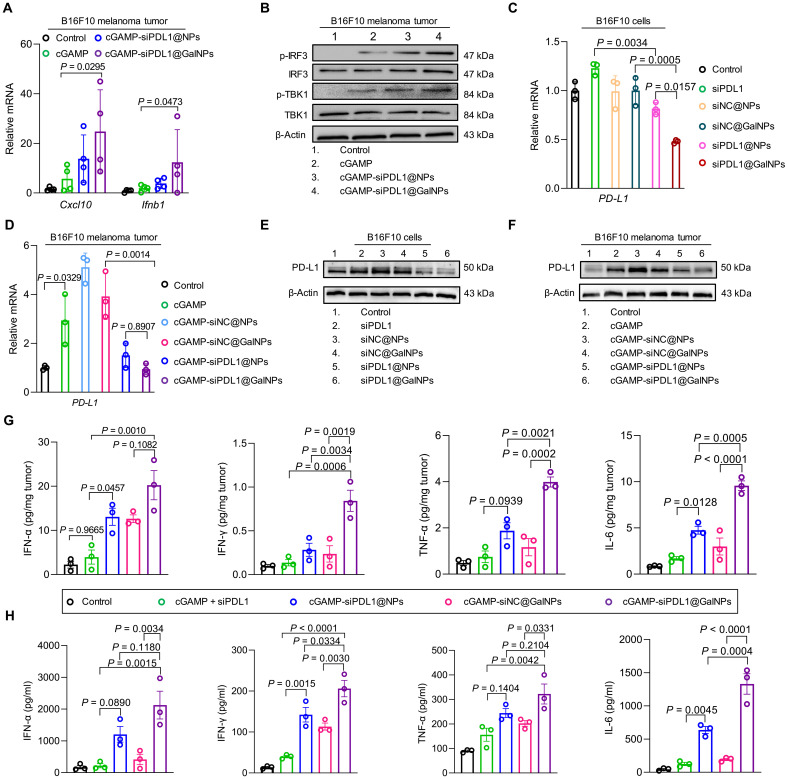
cGAMP-siPDL1@GalNPs activated the STING pathway and down-regulated PD-L1 expression in both cancer cells and tumors. (**A**) qPCR analysis of *Ifnb1* and *Cxcl10* expression in B16F10 tumors after drug administration (*n* = 4 mice). (**B**) WB analysis of STING pathway activation in B16F10 tumors after drug administration. p-IRF3, phosphorylation of IRF3; p-TBK1, phosphorylation of TBK1. (**C** and **D**) qPCR analysis of *PD-L1* mRNA levels in B16F10 cancer cells (C) and B16F10 tumors (D) after treatment with drugs (*n* = 3 mice). (**E** and **F**) WB analysis of PD-L1 expression in B16F10 cancer cells (E) and B16F10 tumors (F) after drug administration. (**G** and **H**) ELISA analysis of IFN-α, IFN-γ, TNF-α, and IL-6 in B16F10 tumors (G) and serum (H) 48 hours after drug administration (*n* = 3 mice). Here are drug concentrations for in vitro (cGAMP = 15 μg and siPDL1/siNC = 15 μg per dose) and in vivo (siPDL1 = 2 μg/ml, NCsiRNA = 2 μg/ml, cGAMP = 100 ng/ml) studies. Data are expressed as means ± SD. Statistical significance was calculated by one-way ANOVA with Tukey test or two-tailed Student’s *t* test when comparing multiple or two groups, respectively.

The STING pathway activation and PD-L1 expression silencing by the cGAMP-siPDL1@GalNPs were evaluated ex vivo and in vivo. To evaluate the STING pathway activation by the cGAMP-siPDL1@GalNPs, the B16F10 melanoma tumors were harvested at 8 hours after administration of the nanocarriers and analyzed by WB and quantitative polymerase chain reaction (qPCR). Compared with the expression levels with free cGAMP + siPDL1 and cGAMP-siPDL1@NPs, the cGAMP-siPDL1@GalNPs significantly increased the expression of *Cxcl10* (4.3-fold higher than that of cGAMP + siPDL1) and *Ifnb1* (5-fold higher than that of cGAMP + siPDL1) (Fig. 5A). In addition, the WB analysis also showed that the cGAMP-siPDL1@GalNPs efficiently elevated the phosphorylation of TBK1 and IRF3 (Fig. 5B).

Recent studies have reported that STING agonists not only stimulate an IFN-I-driven innate immune response but also increase PD-L1 expression in cancer cells (*70*), which blocks the antitumor immunity of CD8^+^ T cells and causes immune escape (*30*, *71*). In this regard, we investigated the PD-L1 expression in cancer cells and tumors and assessed the effects of siPDL1 on PD-L1 expression knockdown by qPCR and WB. As shown in Fig. 5C, the *PD-L1* mRNA levels of the B16F10 cells were significantly down-regulated by the siPDL1@NPs and siPDL1@GalNPs in B16F10 cells compared with that by the siNC@NPs and siNC@GalNPs, indicating the successful RNAi of PD-L1 in cancer cells by the siPDL1 delivery nanocarriers. The in vivo experiments revealed that cGAMP, cGAMP-siNC@NPs, and cGAMP-siNC@GalNPs increased the *PD-L1* mRNA levels in the B16F10 tumors than in the control group by 2.8-, 5.1-, and 3.9-fold, respectively, whereas the PD-L1 expression levels in the cGAMP-siPDL1@NPs and cGAMP-siPDL1@GalNPs groups were similar to those in the control (Fig. 5D). These results indicate that codelivering of siPDL1 can eliminate the immune escape factor PD-L1 produced by the STING agonist cGAMP, thereby facilitating tumor immunotherapy. Similarly, WB analysis was also confirmed that cGAMP and the cGAMP-loaded control nanocarriers promoted PD-L1 expression, whereas the siPDL1-loaded nanocarriers down-regulated PD-L1 expression in both B16F10 cancer cells and melanoma tumors (Fig. 5, E and F). Besides, we evaluated the PD-L1 silencing effect of cGAMP-siPDL1@GalNPs upon laser irradiation (cGAMP-siPDL1@GalNPs + L) in both B16F10 cells and B16F10 tumors by WB and qPCR. Compared to cGAMP-siPDL1@GalNPs and cGAMP-siNC@GalNPs + L groups, cGAMP-siPDL1@GalNPs + L significantly down-regulated the *PD-L1* mRNA expression in the B16F10 cells (fig. S22A) and melanoma tumors (fig. S22B). Consistent with the qPCR results, WB results further confirmed that cGAMP-siPDL1@GalNPs + L forcefully down-regulated the PD-L1 expression in the B16F10 cells (fig. S22C) and melanoma tumors (fig. S22D). Therefore, cGAMP-siPDL1@GalNPs + L can effectively reduce PD-L1 expression both in vivo and in vitro. Here, the cGAMP-siPDL1@NPs and cGAMP-siPDL1@GalNPs efficiently activated the STING pathway and immune responses, and siPDL1 delivery down-regulated the expression of immunosuppressive PD-L1 in cancer cells and tumors. An enzyme-linked immunosorbent assay (ELISA) further demonstrated that the cGAMP-siPDL1@GalNPs significantly triggered the highest expression levels of other immune factors, including IFN-α, IL-6, IFN-γ, and TNF-α, in the B16F10 melanoma tumors and serum (Fig. 5, G and H). In summary, the nanocarriers, especially the cGAMP-siPDL1@GalNPs, efficiently activated the STING pathway and induced cytokine production in the TME.

### cGAMP-siPDL1@GalNPs reprogram the immune TME

**Fig. 6.**
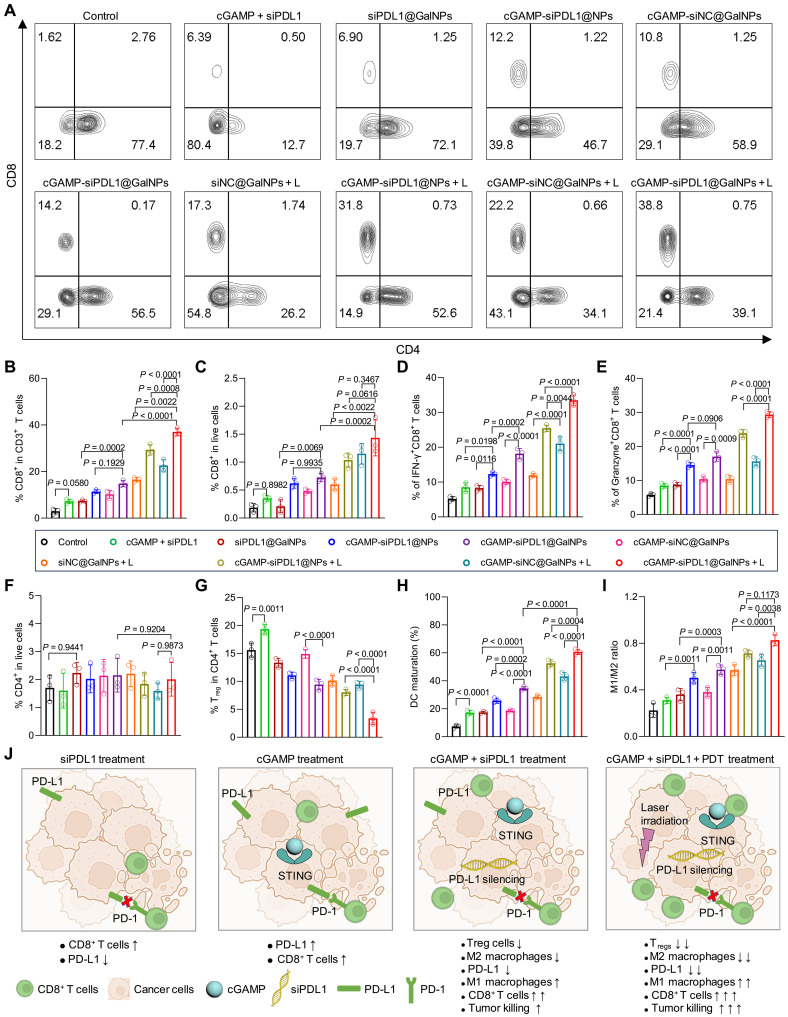
cGAMP-siPDL1@GalNPs synergistically stimulate robust antitumor immune responses and down-regulate immunosuppressive factors in TME. (**A**) Representative FCM dot plots of tumor-infiltrating CD4^+^ and CD8^+^ T cells (gated by CD45^+^CD3^+^) at 48 hours after drug administration and followed with/without laser irradiation (660 nm, 100 mW/cm^2^, 5 min). (**B** and **C**) Quantifying the percentages of infiltrating CD8^+^ T cells among all live cells (B) and within CD3^+^ cells (C) in B16F10 TME after treatments. (**D** and **E**) Quantitative analysis of tumor-infiltrating IFN-γ^+^CD8^+^ T cells (D) and granzyme B^+^CD8^+^ T cells (E) in tumors. (**F**) Percentages of CD4^+^ T cells in TME among all live cells. (**G** and **H**) Quantification of T_regs_ (CD4^+^CD25^+^Foxp3^+^) (G) and DC maturation (H) in B16F10 tumors. (**I**) Comparison between M1-type (CD86^+^CD11b^+^F4/80^+^) and M2-type (CD206^+^CD11b^+^F4/80^+^) macrophages in B16F10 tumors. (**J**) The cGAMP-siPDL1@GalNPs synergistically stimulated anticancer immune responses including IFN-γ^+^CD8^+^ T cells, granzyme B^+^CD8^+^ T cells, and M1-type macrophages and down-regulated immunosuppressive factors including T_regs_, M1-type macrophages, and PD-L1 expression in TME. Data are expressed as means ± SD (*n* = 3 mice). PD-1, programmed cell death protein 1. Statistical significance was determined using one-way ANOVA with Tukey’s multiple comparisons test.

STING agonists stimulate the T cell immune response and up-regulate PD-L1, as shown in numerous tumor models, which convert low immunogenic cold tumors to hot tumors (*70*). Although the PD-L1 up-regulation causes the immune escape of tumor cells to T cell–mediated killing (*71*), combining STING agonists with siPDL1 would promote tumor cell killing by intratumoral CD8^+^ T cells. In addition, the PDT of primary tumors could cause the ICD of cancer cells to activate the immune responses of effector T cells (*66*). To examine the regulation of the immunosuppressive TME, the B16F10 melanoma tumors were irradiated with a laser (660 nm, 100 mW/cm^2^, 5 min) at 6 hours after the nanocarriers administration, with the tumors collected at 48 hours for analyzing the major immune cells. As shown in Fig. 6 (A and B), the cGAMP-siPDL1@GalNPs + L considerably enhanced the populations of CD8^+^ T cells, which were 1.63-, 1.26-, 2.24-, and 2.49-fold compared to cGAMP-siNC@GalNPs + L, cGAMP-siPDL1@NPs + L, siNC@GalNPs + L, and cGAMP-siPDL1@NPs, respectively. Notably, cGAMP-siPDL1@GalNPs + L generated a higher rate of CD8^+^ T cells than cGAMP-siPDL1@GalNPs, because of the additional ICD effect (Fig. 6, B and C). Moreover, further investigations show that cGAMP-siPDL1@GalNPs + L yielded the highest rates of IFN-γ^+^CD8^+^ and granzyme B–secreting CD8^+^ T cells (granzyme B^+^CD8^+^) in the melanoma TME than the other treatments (Fig. 6, D and E, and fig. S23, A and B). Thus, cGAMP-siPDL1@GalNPs + L activated and promoted the highest rate of tumor-killing CD8^+^ T cells in TME.

Although the CD4^+^ T cell rates were almost identical (Fig. 6F), the nanocarrier-based therapies increased the CD8^+^/CD4^+^ rates, with cGAMP-siPDL1@GalNPs + L yielding the highest CD8^+^/CD4^+^ rate (fig. S24A). The cGAMP-siPDL1@GalNPs + L exhibited the greatest ability to down-regulate the immunosuppressive regulatory T cells (T_regs_) in TME compared to cGAMP-siPDL1@NPs + L, cGAMP-siNC@NPs + L, etc. (Fig. 6G and fig. S23C). Next, we assessed the DC maturation and macrophage polarization. Both cGAMP-siPDL1@NPs + L and cGAMP-siPDL1@GalNPs + L promoted the populations of DCs (fig. S24B) and M1 macrophages (fig. S24C) in TME than the others, e.g., cGAMP-siNC@GalNPs, leading to an innate inflammatory niche with the potential to trigger adaptive immunity. As shown in Fig. 6H and fig. S25A, higher rates of DC maturation were obtained by cGAMP-siPDL1@NPs + L (1.85-fold) and cGAMP-siPDL1@GalNPs + L (2.14-fold) compared to siNC@GalNPs + L (Fig. 6H and fig. S25A). In addition, the intratumoral infiltrating proinflammatory M1 and anti-inflammatory M2 macrophages were investigated, which also have immune functions (*72*). The nanocarrier-based therapies yielded high M1/M2 ratios (Fig. 6I and fig. S25, B and C), which can help tumor immunotherapy. Therefore, cGAMP-siPDL1@GalNPs + L efficiently modulated the TME, not only by promoting immune effector cells, such as CD8^+^ T cells, IFN-γ^+^CD8^+^ T cells, granzyme B^+^CD8^+^ T cells, M1 macrophages, and matured DCs, but also by down-regulating immunosuppressive factors, such as T_regs_, PD-L1, and M2 macrophages (Fig. 6J). The combined administration of cGAMP and siPDL1 within the nanocarriers synergistically promoted anticancer immunity.

### cGAMP-siPDL1@GalNPs for immunotherapy of primary and distant B16F10 melanoma tumors

**Fig. 7.**
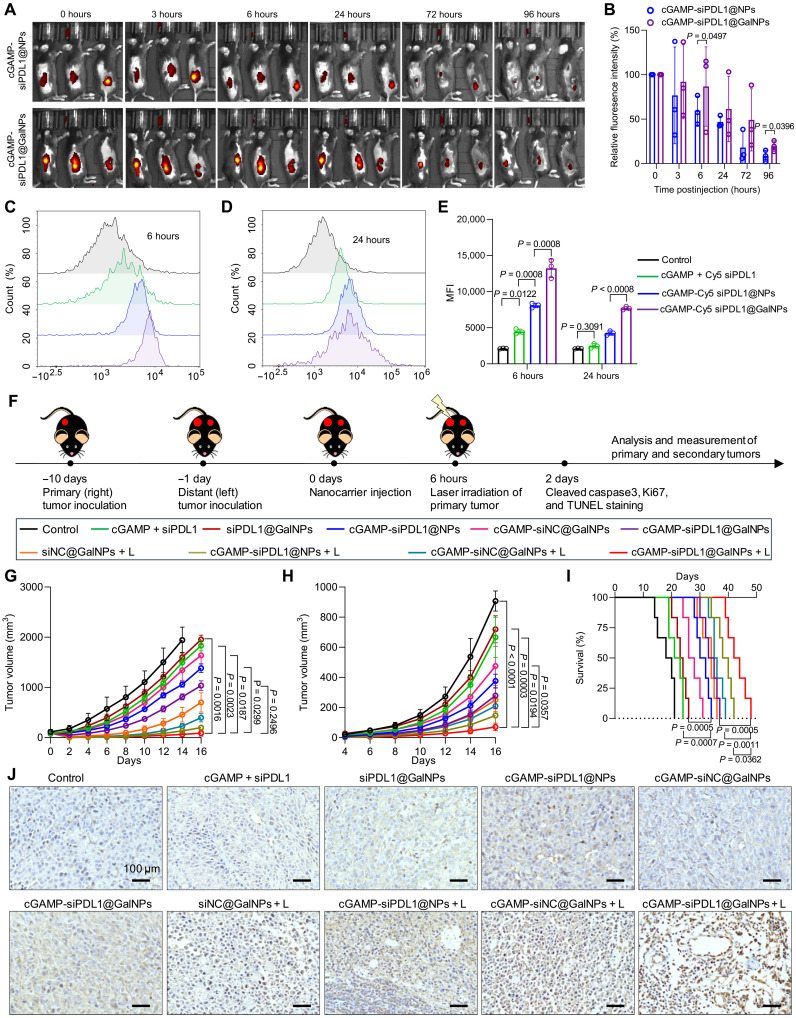
cGAMP-siPDL1@GalNPs for effective immunotherapy of primary and distant B16F10 melanoma tumors. (**A** and **B**) IVIS images (A) and quantitative analysis (B) show cGAMP-siPDL1@NPs and cGAMP-siPDL1@GalNPs in B16F10 melanoma tumors after drug administration (*n* = 3 mice, two-tailed unpaired *t* test). (**C** to **E**) The in vivo cellular uptake of nanocarriers by cancer cells isolated from B16F10 melanoma tumors at 6 (C) and 24 hours (D) after administration of nanocarriers, and the MFI of Cy5 siPDL1 within the cancer cells was calculated (E) (*n* = 3 mice, one-way ANOVA with Tukey test). (**F**) Experimental setup for immunotherapy of subcutaneous B16F10 tumors. C57BL/6 mice were implanted with primary and distant B16F10 melanoma tumors, with only the primary tumors intratumorally injected with drugs (cGAMP = 15 μg and siPDL1/siNC = 15 μg per dose) and received with/without laser irradiation (660 nm, 100 mW/cm^2^, 5 min). (**G** and **H**) Average tumor growth curves of primary (G) and distant (H) B16F10 melanoma tumors posttreatment (*n* = 6 mice, Kruskal-Wallis test with Dunn’s multiple comparisons test). (**I**) Overall survival rates of B16F10 tumor–bearing mice posttreatments (*n* = 6 mice, two-tailed Mantel-Cox test). (**J**) Representative images show IHC staining of cleaved caspase-3 in B16F10 melanoma tumors. Data are expressed as means ± SD.

Influenced by their strong antitumor immune responses, the nanocarriers were used in the immunotherapy of B16F10 melanoma primary tumors, with the inhibition of distant tumors by the stimulated immune responses was also tested. For in vivo immunotherapy, the tumor retention of the cGAMP-siPDL1@NPs and cGAMP-siPDL1@GalNPs was evaluated using an in vivo imaging system (IVIS), since the PPa can be detected with the IVIS. As shown in Fig. 7 (A and B), the fluorescence intensity of the cGAMP-siPDL1@NPs was rapidly dropped at 3 hours, while the retention of the cGAMP-siPDL1@GalNPs remained largely unchanged until 6 hours. Thus, the tumors were irradiated with a laser at 6 hours after the drug administration, after the diffusion and retention of the nanocarriers. Besides, B16F10 melanoma tumors were harvested at 6 and 24 hours after the administration of cGAMP-Cy5 siPDL1@GalNPs to evaluate the in vivo cellular uptake by FCM. As shown in Fig. 7 (C to E), cGAMP-Cy5 siPDL1@GalNPs exhibited the highest rate of cellular uptake by the B16F10 cancer cells in TME than cGAMP-Cy5 siPDL1@NPs and cGAMP + Cy5 siPDL1 at 6 and 24 hours. Immunotherapeutic efficacy was evaluated in B16F10 melanoma tumor–bearing C57BL/6 mice. B16F10 primary tumors were inoculated in the right flanks of mice (day −10), and the distant tumors were made in the left flanks of the same mice 9 days later. Only the primary tumors were irradiated by laser (660 nm, 100 mW/cm^2^, 5 min) at 6 hours after administering nanocarriers (day 0) (Fig. 7F). As shown in Fig. 7G and fig. S26, cGAMP-siPDL1@NPs and cGAMP-siPDL1@GalNPs–based therapies can inhibit the growth of primary tumors, but only cGAMP-siPDL1@GalNPs + L nearly eliminated the primary tumors. Conversely, the primary tumors grew rapidly in the control, cGAMP + siPDL1, siPDL1@GalNPs, and cGAMP-siNC@GalNPs groups. The distant tumors were effectively inhibited in mice with primary tumors treated with nanocarriers plus laser irradiation, such as cGAMP-siPDL1@GalNPs + L (Fig. 7H and fig. S27). In addition, the survival time of cGAMP-siPDL1@GalNPs + L group (48 days) was much longer than that of the cGAMP-siPDL1@NPs + L group (36 days), while those in the other groups died rapidly (Fig. 7I). The primary B16F10 melanoma tumors were obtained on day 2 for immunohistochemical (IHC) staining of cleaved caspase-3 and immunofluorescent staining of Ki67 and terminal deoxynucleotidyl transferase–mediated deoxyuridine triphosphate nick end labeling (TUNEL). Cleaved caspase-3 and TUNEL staining revealed that cGAMP-siPDL1@GalNPs + L induced the highest level of apoptosis of tumor cells compared to the others, e.g., cGAMP-siNC@GalNPs + L group (Fig. 7J and fig. S28). Ki67 staining further revealed that cGAMP-siPDL1@GalNPs + L treatment effectively inhibited the proliferation of B16F10 tumor cells (fig. S29). Those results demonstrated that the nanocarriers supplemented with PDT could effectively eradicate primary tumors and trigger systemic antitumor immunity to suppress the growth of distant tumors and promote the survival rate.

### cGAMP-siPDL1@GalNPs for immunotherapy of orthotopic breast tumors and the spontaneous metastasis

**Fig. 8.**
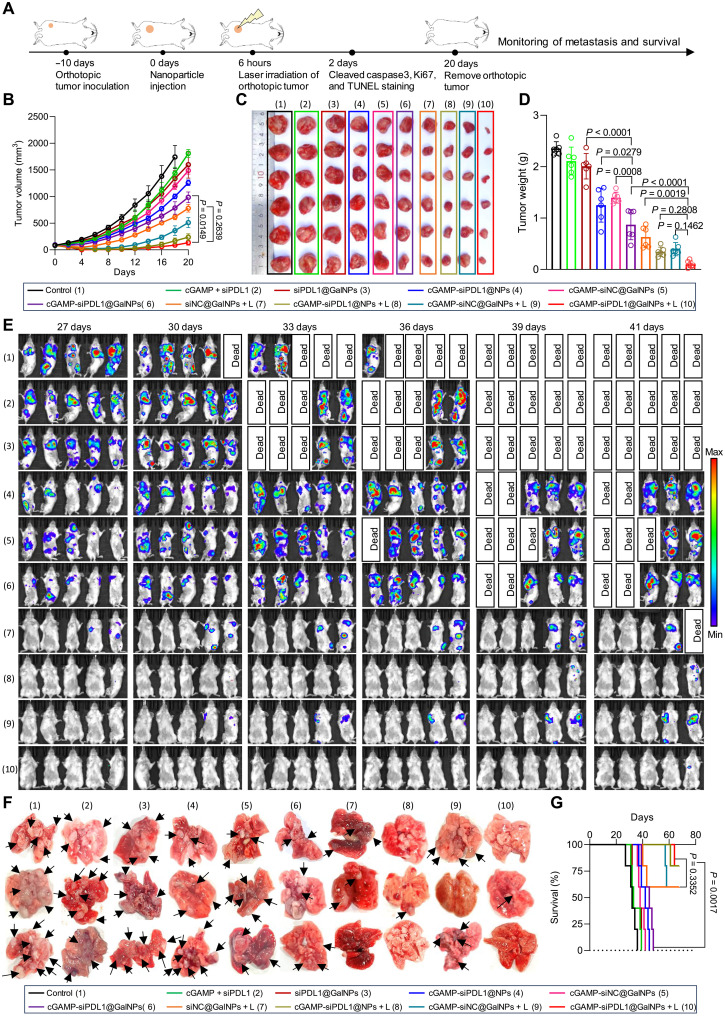
cGAMP-siPDL1@GalNPs for the effective immunotherapy of triple-negative breast tumors and inhibited lung metastasis. (**A**) Experimental setup to study the immunotherapeutic effects in mice bearing orthotropic 4T1-Luc breast tumors. The mice were successively implanted with orthotopic 4T1-Luc breast tumors, which were injected with the drugs (cGAMP = 15 μg and siPDL1/siNC = 15 μg per dose) to receive with/without laser irradiation (660 nm, 100 mW/cm^2^, 5 min) on day 0. The orthotopic 4T1-Luc tumors were surgically removed on day 20. The development of spontaneous lung metastases was monitored using IVIS. (**B**) Growth curves of the orthotopic 4T1-Luc breast tumors posttreatments (*n* = 6 mice, Kruskal-Wallis test with Dunn’s multiple comparisons test). (**C** and **D**) Images (C) and (D) the average weights of the orthotopic tumors after the treatments (*n* = 6 mice, one-way ANOVA with Tukey’s multiple comparisons test). (**E**) IVIS images show spontaneous lung metastases from the orthotopic 4T1-Luc tumors in each group. (**F**) Ex vivo images show lungs with metastatic breast tumors (*n* = 3 mice). Black arrows point to lung metastatic foci. (**G**) Survival rates of mice after receiving the different treatments (*n* = 5 mice, two-tailed Mantel-Cox test). Data are expressed as means ± SD.

On the basis of the immunotherapy strategies of melanoma tumors, the nanocarriers were then used to treat triple-negative breast tumors, since breast cancer is the most common cancer and accounts for 2.3 million new cases and 685,000 deaths annually, as per recent reports (*73*, *74*). However, the breast tumors have low immunogenicity and demonstrate low responses to immunotherapy (*75*). Therefore, there is a demand of developing effective therapeutic approaches to treating triple-negative breast cancer, such as cGAMP-siPDL1@GalNPs. First, the retention of nanocarriers in the orthotopic 4T1 breast tumors was checked by IVIS. At 6 hours after drug administration, the fluorescence intensity of nanocarriers in tumors was still high, but it decreased at 12 hours (fig. S30). Therefore, we conducted laser irradiation of the tumors at 6 hours after drug administration. Female BALB/c mice were established with orthotopic 4T1-Luc breast tumors on the right fourth mammary fat pad. The nanocarriers were administrated on day 0, with the orthotopic tumors irradiated by laser (660 nm, 100 mW/cm^2^, 5 min) at 6 hours. The volumes of the orthotopic tumors were monitored. The orthotopic 4T1-Luc tumor was surgically removed and weighed on day 20, while the development of spontaneous lung metastases was monitored with an IVIS spectrum (Fig. 8A). As shown in Fig. 8 (B to D) and fig. S31, both cGAMP-siPDL1@NPs + L and cGAMP-siPDL1@GalNPs + L treatments effectively eradicated the orthotopic breast tumors, only cGAMP-siPDL1@GalNPs + L achieved the lowest tumor growth rate, weight, and size. However, the tumors grew quickly in the control, cGAMP + siPDL1, siPDL1@GalNPs, cGAMP-siPDL1@NPs, and cGAMP-siNC@GalNPs groups, associating with bigger tumor volumes and higher tumor weights. The orthotopic breast tumors were collected on day 2 for staining cleaved caspase-3, Ki67, and TUNEL, respectively. Cleaved caspase-3 and TUNEL staining revealed that cGAMP-siPDL1@GalNPs + L induced the highest level of cancer cell apoptosis (figs. S32 and S33). Ki67 staining revealed that nanocarriers plus laser irradiation (cGAMP-siPDL1@GalNPs + L) effectively terminated the proliferation of cancer cells in the orthotopic breast tumors compared to that without laser irradiation (fig. S34). Moreover, the spontaneous lung metastases from the orthotopic 4T1-Luc tumors were also effectively inhibited by the cGAMP-siPDL1@GalNPs + L treatment, as monitored by IVIS. Lung metastases developed aggressively in the control, cGAMP + siPDL1, siPDL1@GalNPs, cGAMP-siPDL1@GalNPs, etc., as demonstrated by high bioluminescence signals and mice death (Fig. 8E). Hematoxylin and eosin (H&E) staining (fig. S35) and images (Fig. 8F) of the lungs revealed numerous metastasis foci in the control, cGAMP + siPDL1, siPDL1@GalNPs, cGAMP-siPDL1@NPs, cGAMP-siNC@GalNPs, and cGAMP-siPDL1@GalNPs, while only fewer metastases were found in the cGAMP-siPDL1@NPs + L group, and the fewest metastasis was found in the cGAMP-siPDL1@GalNPs + L group. Notably, the cGAMP-siPDL1@GalNPs + L obtained a high survival rate (75%) of mice within 68 days; however, mice in most control groups died within 48 days (Fig. 8G). Overall, the cGAMP-siPDL1@GalNPs nanocarrier-based PDT could effectively eradicate primary triple-negative breast tumors and suppress its lung metastasis through the combined immunotherapy.

### Safety evaluation

To evaluate the safety of the nanocarrier-based therapies, the therapeutic experiments on mice involved body weight monitoring as well as hematological testing and H&E staining of major organs (i.e., heart, liver, spleen, lung, and kidney). No obvious changes in the body weight were observed in the above animal experiments (figs. S36 and S37). In addition, no visible morphological damages to the major organs were detected on comparing the H&E staining in the control and treatment groups (fig. S38). Moreover, the major factors in the routine blood examinations were within the normal ranges, including the red blood cell, white blood cell, hematocrit, platelet, and lymphocyte (fig. S39A). Furthermore, no significant differences were observed for the serum biochemistry indicators, including alanine aminotransferase, aspartate transaminase, creatinine, and urea (fig. S39B). These results demonstrated the biosafety and biocompatibility of the cGAMP- and siPDL1-loaded nanocarriers.

## DISCUSSION

In summary, we synthesized a triblock cationic polymer comprising a photosensitizer and targeting ligand to coencapsulate cGAMP and siPDL1 for synergistic cancer immunotherapy. The galactose-installed nanocarriers cGAMP-siPDL1@GalNPs efficiently delivered the cGAMP and siPDL1 into the target cancer cells and controlled their release through a photo-/redox-/pH-activated drug delivery approach. The cGAMP-siPDL1@GalNPs nanocarriers effectively and selectively activated the STING pathway in cancer cells via GLUT-1 to increase the biological activity of cGAMP, which stimulated a series of immune responses, and down-regulated the PD-L1 expression in cancer cells and tumors to prohibit the immune escape and eliminate the side effects of STING agonists. Of note, tumor cells rely on glucose metabolism to obtain a large amount of energy, leading to overexpression of GLUT-1 in a wide range of tumors, including hepatic, pancreatic, breast, esophageal, brain, renal, lung, cutaneous, colorectal, endometrial, ovarian, and cervical cancers (*76*). Conversely, sarcomas, lymphomas, and hepatoblastomas did not express GLUT-1 (*77*). In our study, we have conducted IHC assays to show that both B16F10 melanoma and 4T1 breast tumors overexpress GLUT-1 (fig. S15). Therefore, galactose-installed cGAMP-siPDL1@GalNPs were able to target those tumors effectively. We chose galactose as the targeting ligand, as it is easily accessible and low cost compared to other ligands (e.g., antibody) and can target receptors overexpressed in several tumors and multiple receptors in tumors (i.e., GLUT-1, galectin-1, and galectin-3) (*58*).

STING is widely expressed across various cell types and plays a critical role in regulation of immune responses (*16*). As a way to escape immune surveillance, the suppression of STING signaling is often observed in cancer cells, including melanoma, lung cancers, and colon cancers (*78*, *79*). Inhibition of the STING pathway in cancer cells decreases the immunogenicity of tumors, including recruitment of T_regs_ into TME and resistance to immune checkpoint inhibitors (*78*). There are many ways to activate STING signaling. For instance, directly STING activation uses natural cyclic dinucleotide STING agonists (e.g., 3′3′-cGAMP, 2′2′-cGAMP, 3′2′-cGAMP, etc.) and non-nucleotide STING agonists (e.g., SR-717, MSA-2, diABZI compound 3, etc.) (*71*). Indirect STING activation can be caused by cellular double-stranded DNA, including chemotherapy (e.g., cisplatin, 5-aza-2′-deoxycytidine, olaparib, doxorubicin, etc.) (*71*), radiotherapy, and some metal ions (e.g., Mn^2+^, Zn^2+^, and Fe^3+^) (*58*, *80*). Notably, PDT-induced generation of ROS can result in mitochondrial dysfunction, leading to the cytosolic release of mitochondrial DNA and subsequent activation of the STING signaling pathway (*81*, *82*). Different from other combinations of STING activation and PDT (*80*, *81*), our STING activation is mainly derived by cGAMP, which activates STING in cancer cells. The photosensitizer PPa can initiate the ring-opening polymerization of the polymers and mediate PCI and ICD effects to promote the silencing effects of siPLD1 and synergize STING agonists to enhance CD8^+^ T cell–mediated antitumor immunity, respectively. The cGAMP-siPDL1@GalNPs remodeled the TME by stimulating a high level of CD8^+^ T cell infiltration in the tumors. The nanocarriers also inhibited the development of primary, distant, and metastatic tumors by activating systemic immune responses. In general, low immunogenicity is a common feature of most tumors, which can be infiltrated by a high amount of immunosuppressive immune cells, including T_regs_ and M2 macrophages. In addition, these tumors lack proinflammatory immune cells and effector T cells, further contributing to poor immunotherapeutic outcomes. Despite the development of immunotherapeutic drugs, such as immune checkpoint inhibitors, it remains a challenge to effectively treat cold tumors, leading to immunotherapeutic failure. Our findings provided a potential immunotherapeutic strategy for treating cold tumors that are difficult to treat by only administrating immune checkpoint blockades. This study further provides a paradigm of combined cancer immunotherapy, which can boost modest immune responses to regulate the immune-suppressive TME of poorly immunogenic tumors, thereby effectively eradicating the primary tumor and inhibiting its systemic development (e.g., metastasis) and facilitating future clinical cancer immunotherapy.

## MATERIALS AND METHODS

### Study design

The aim of this study was to develop photo-/redox-/pH-activatable nanocarriers that can activate immune responses by STING activation and ICD effects and inhibit immune evasion by knockdown of PD-L1 expression, for synergistic cancer immunotherapy. We synthesized functional polymers, characterized nanocarriers, and screened nanocarriers with different N/P ratios to obtain the best gene-silencing efficacy. The intracellular delivery of nucleic acid drugs by PCI was analyzed. To investigate the ICD effects, activation of the STING pathway, and the down-regulation of PD-L1, we analyzed CRT, HMGB1, ATP, STING, and PD-L1–related mRNA expression and proteins by using CLSM, qPCR, WB, etc. We confirmed the biodistribution of nanocarriers using IVIS, and then the antitumor efficacy was evaluated in B16F10 melanoma and orthotopic 4T1 breast tumor models. Immune cells and cytokines in the TME were also analyzed using FCM and ELISA, respectively. The toxicity of cGAMP-siPDL1@GalNPs was studied by checking body weight, blood test, and pathological study. Mice were randomly divided into different treatment groups. The replicate numbers for each experiment and statistical analysis methods are indicated in the figure legends. All animal experiments involved in this study were conducted according to protocols evaluated and approved by the ethical committee of Sichuan University (20230302063).

### Preparation of cGAMP and siPDL1-incorporated multifunctional nanocarriers

The Gal-PEG-PAsp(DET/MPA)-PPa or PEG-PAsp(DET/MPA)-PPa polymer solution (10 mg/ml) was dissolved in Hepes buffer (10 mM, pH 7.4). siPDL1 and cGAMP were also dissolved in HEPES buffer (10 mM, pH 7.4). The polymer solution was added to a 2-times-excess volume siPDL1 and cGAMP solution (N/P molar ratio = 20:1), and then the mixture was incubated at 4°C for 30 min to obtain nanocarriers cGAMP-siPDL1@NPs [made by PEG-PAsp(DET/MPA)-PPa] and cGAMP-siPDL1@GalNPs [made by 25% Gal-PEG-PAsp(DET/MPA)-PPa and 75% PEG-PAsp(DET/MPA)-PPa], respectively. Similarly, we prepared nanocarriers siNC@NPs, siNC@GalNPs, cGAMP-siNC@NPs, and cGAMP-siNC@GalNPs. The diameter and PDI were detected by Zetasizer Nano ZS90 (Malvern, UK). The zeta potential values of nanocarriers were measured in phosphate buffer (10 mM, pH 7.4) with Zetasizer Nano ZS90 (Malvern, UK). The morphology of nanocarriers was characterized by TEM (JEOL, JEM-2100Plus, Japan). The stability of nanocarriers was obtained by checking their diameter, and PDI was changed in medium (RPMI 1640 containing 10% fetal bovine serum). The release kinetics and EE of cGAMP were detected by HPLC (LC-2050C, Shimadzu, Japan). The mobile phase consisted of (A) 0.1% trifluoroacetic acid (aqueous) and (B) acetonitrile. Gradient elution: 0.01 to 20 min, B in A, 5 to 80%. The UV detector (λ = 254 nm) monitored the peak time of cGAMP at 4.8 min. The EE of siPDL1 were detected using RiboGreen reagent (catalog no. P9745, Solarbio).

### Gel electrophoresis

One gram of agarose is added in tris-acetate-EDTA (TAE) buffer (50 ml, pH 8.3), microwaved for 5 min to dissolve the agarose, and cooled down for adding ethidium bromide (2.5 μl). The nanocarriers solution (10 μl, contained 1 μg of siPDL1) was incubated with/without DTT (5 μl, 200 mM) and heparin (5 μl, 40 mg/ml) at 4°C for 12 hours. The final DTT and heparin concentration were 50 mM and 10 mg/ml, respectively. Then, each sample was loaded on the gel and ran electrophoresis (120 V, 30 min) in TAE buffer (pH 8.3). The band of siPDL1 was detected using a Gel imaging system (Bio-Rad, Gel Doc XR, USA).

### Fluorescence intensity of nanocarriers

cGAMP-Cy5 siPDL1@NPs or cGAMP-Cy5 siPDL1@NPs (20 μl) was diluted in Hepes buffer with different pH values (100 μl, 10 mM). GSH solutions of different pH values are added the previous solution. The final polymer concentration was 0.1 mg/ml. The final GSH concentration was 10 or 0.01 mM. The mixture (100 μl) was added to black 96-well plates with clear bottom. The fluorescence intensity of Cy5 siPDL1 was obtained on a multifunctional microplate reader (Infinite M200 Pro, Tecan, Switzerland). The mixtures were excited at 650 nm, and the emission intensity was recorded at 670 nm.

### Generation of ROS by nanocarriers

In vitro generation of ROS (i.e., ^1^O_2_) by nanocarriers was detected using DPBF as an indicator. DPBF (20 μl, 5 mM) dissolved in ethanol was mixed with cGAMP-siPDL1@NPs and cGAMP-siPDL1@GalNPs (1 ml, 20 μg/ml based on PPa) to receive laser irradiation (660 nm, 100 mW/cm^2^) for different times. Then, the absorbance spectra of the solution were detected using a UV-vis spectrophotometer (UV2550, Shimadzu, Japan).

### Intracellular generation of ROS by nanocarriers

The B16F10 cancer cells (2 × 10^4^ per well) were seeded in a confocal dish for 24 hours and exposed to cGAMP-siPDL1@NPs or cGAMP-siPDL1@GalNPs (20 μg/ml based on PPa) for 6 hours. Phosphate-buffered saline (PBS) buffer washed off the residual nanocarriers. Then, the cells were stained with DCFH-DA (10 μM; catalog no. CA1410, Solarbio) for 20 min under 37°C. DCFH-DA was washed off with PBS buffer. The cells were irradiated with a laser at 660 nm (100 mW/cm^2^, 5 min). Last, the nuclei of cells were stained by Hoechst 33342 (catalog no. C1026, Beyotime). ROS was detected via CLSM (Zeiss LSM 880, Zeiss, Germany). For FCM analysis, B16F10 cells (2 × 10^5^ per well) were added to six-well plates and exposed nanocarriers, stained with DCFH-DA subsequently as described above, and washed with PBS. After that, the cells were collected and dispersed in 0.4 ml of PBS for checking using a flow cytometer (NovoCyte, Agilent, USA).

### Cellular uptake

The B16F10 cells (2 × 10^4^ per well) were seeded in a confocal dish for 24 hours and exposed to cGAMP-siPDL1@NPs or cGAMP-siPDL1@GalNPs (20 μg/ml based on PPa) for 3 and 6 hours. The cells were divided into six groups as follows: 3 hour–cGAMP-siPDL1@NPs group, 3 hour–cGAMP-siPDL1@GalNPs group, 6 hour–cGAMP-siPDL1@NPs group, 6 hour–cGAMP-siPDL1@GalNPs group, 6 hour–cGAMP-siPDL1@NPs + L group, and 6 hour–cGAMP-siPDL1@GalNPs + L group (L refers to laser irradiation). Then, the cells were washed with PBS buffer and stained by LysoTracker Green (50 nM; catalog no. C1047S, Beyotime) for 50 min for labeling lysosomes/endosomes and Hoechst 33342 (catalog no. C1026, Beyotime) for labeling nuclei. For L groups, the cells received with laser irradiation at 660 nm (5 min, 100 mW/cm^2^) after washed with PBS, incubated for another 4 hours, and stained with LysoTracker Green and Hoechst 33342 as described above. All the cells were examined using CLSM (Zeiss LSM 880, Zeiss, Germany). Besides, the B16F10 cells (2 × 10^5^ per well) were added to six-well plates for 24 hours and exposed to cGAMP-siPDL1@NPs or cGAMP-siPDL1@GalNPs (20 μg/ml based on PPa) for 3 and 6 hours. After washing with PBS buffer, the cells were collected in PBS buffer, and the cellular uptake was examined using a flow cytometer (NovoCyte, Agilent, USA).

### Block cellular uptake/identification of cellular uptake pathways

The B16F10 cells (2 × 10^4^ per well) were added to a confocal dish for 24 hours and exposed to cGAMP-siPDL1@NPs or cGAMP-siPDL1@GalNPs (20 μg/ml based on PPa) for 6 hours. Phloretin (0.2 mM) as a GLUT-1 inhibitor was added 30 min before the addition of nanocarriers. Then, the cells were washed with PBS and stained with Hoechst 33342 (catalog no. C1026, Beyotime) for labeling nuclei. After that, the internalized nanocarriers within cells were detected by CLSM (Zeiss LSM 880, Zeiss, Germany). Besides, the B16F10 cells (2 × 10^5^per well) were added in six-well plates, cultured at 37°C for 24 hours, and then exposed to phloretin (0.2 mM) and cGAMP-siPDL1@NPs or cGAMP-siPDL1@GalNPs (20 μg/ml based on PPa) for 6 hours as described above, respectively. After washing with PBS buffer, the cells were collected in PBS buffer, and subsequently, cellular uptake was examined using a flow cytometer (NovoCyte, Agilent, USA).

### Cell viability assay

The B16F10 cells (4000 cells per well) were seeded in 96-well plates, cultured in a cell culture incubator for 24 hours, and exposed to cGAMP-siPDL1@NPs and cGAMP-siPDL1@GalNPs (cGAMP = 100 ng/ml, siPDL1 = 10 μg/ml, PPa = 43 μg/ml) for 6 hours. After washing with PBS buffer and adding 100 μl of fresh medium, the B16F10 cells received with or without laser irradiation at 660 nm (5 min, 100 mW/cm^2^) and incubated for another 24 hours. Cell counting kit-8 solution (10 μl per well) was added to the 96-well plate to incubate for 2 hours. Last, the cell viability was assessed by measuring the absorbance at 450 nm via a microplate reader (Infinite M200 Pro, Tecan, Switzerland).

### Induction of ICD with nanocarriers

To determine nanocarrier-induced ICD in vitro, surface expression of CRT, HMGB1 release, and ATP secretion were examined. The commercial ATP assay kit (catalog no. S0026, Beyotime) was used to test intracellular and extracellular ATP. Briefly, B16F10 cells (1 × 10^5^ cells per well) were seeded on 12-well plates and incubated for 24 hours. The cells were treated with cGAMP-siPDL1@NPs or cGAMP-siPDL1@GalNPs (20 μg/ml based on PPa) for 6 hours. After washing with PBS buffer and adding 1 ml of fresh medium, the cells received with or without laser irradiation at 660 nm (5 min, 100 mW/cm^2^) and incubated for another 4 hours. The cell culture supernatant was collected, and the sample was placed on ice. The B16F10 cells were treated with 100 μl of ATP lysis buffer from the ATP assay kit, and ATP lysis buffer was collected and placed on ice. The ATP concentration was detected using the ATP assay kit (catalog no. S0026, Beyotime).

Furthermore, the IF analysis was used to observe intracellular HMGB1 and cell surface CRT distribution. The B16F10 cells (2 × 10^4^ per well) were seeded in a confocal dish and incubated for 24 hours. The cells were treated with cGAMP-siPDL1@NPs or cGAMP-siPDL1@GalNPs (20 μg/ml based on PPa) for 6 hours. After washing with PBS buffer and adding 1 ml of fresh medium, the B16F10 cells received with or without a leaser irradiation at 660 nm (5 min, 100 mW/cm^2^) and incubated for another 4 hours. The cells were fixed in 4% paraformaldehyde (PFA) for 15 min at 37°C and permeabilized with 0.3% Triton X-100 for 5 min. Then, nonspecific binding sites were blocked with 5% bovine serum albumin (BSA) for 1 hour. The cells were incubated with the primary antibody HMGB1 at 4°C for 12 hours and then incubated with an iFluor 488–conjugated secondary antibody (1:1000) for 1 hour. Last, the cells were stained with 4′,6-diamidino-2-phenylindole (DAPI; catalog no. C1002, Beyotime) and observed using CLSM (Zeiss LSM 880, Zeiss, Germany). For IF staining of CRT, the cells were fixed in 4% PFA for 15 min at 37°C and blocked with 5% BSA for 1 hour, followed by incubation with primary antibody CRT (1:500) at 4°C for 12 hours, and then incubated with an iFluor 488–conjugated secondary antibody (1:1000) for 1 hour. Last, the cells stained with 1,1′-dioctadecyl-3,3,3′,3′-tetramethylindocarbocyanine perchlorate (Dil; catalog no. C1991S, Beyotime) 15 min at 37°C and DAPI (catalog no. C1002, Beyotime) to label cell membrane and nuclei, respectively. Cell surface CRT was observed using CLSM (Zeiss LSM 880, Zeiss, Germany).

Besides, WB was used to analysis intracellular HMGB1 expression. B16F10 cells were seeded in six-well plates at a density of 2 × 10^5^ cells per well and incubated for 24 hours. The cells were treated with cGAMP-siPDL1@NPs or cGAMP-siPDL1@GalNPs (20 μg/ml based on PPa) for 6 hours. After washing with PBS buffer and adding 1 ml of fresh medium, the cells received with or without laser irradiation at 660 nm (5 min, 100 mW/cm^2^) and incubated for another 4 hours. The cells were lysed by radioimmunoprecipitation assay (RIPA) lysis buffer (catalog no. P0013B, Beyotime) and then centrifuged (12,000 rpm, 10 min) to collect the supernatant. The concentration of protein solution was detected using a BCA protein assay kit (catalog no. P0012S, Beyotime). The sample were separated on 10% SDS polyacrylamide gels (catalog no. PG114, Epizyme). Proteins were transferred to a polyvinylidene difluoride (PVDF) membrane (200 mA, 1 hour), blocked with 5% BSA, and washed with tris-buffered saline containing 0.05% Tween 20 (TBST). The PVDF membrane was incubated with the primary antibody HMGB1 (1:5000) and β-actin (1:2000) at 4°C for 12 hours. After washed with TBST, the PVDF membranes were incubated with a secondary antibody (1:50,000) for 1 hour. Last, the PVDF membranes were tested using an eBLOT touch imager (eBLOT, China).

### WB and qPCR analyzing the activation of STING and down-regulation of PD-L1 in B16F10 cancer cells and tumors

Female C57BL/6 mice bearing B16F10 tumors were intratumorally injected with cGAMP, cGAMP-siPDL1@NPs, and cGAMP-siPDL1@GalNPs (25 μl; 15 μg of cGAMP per mouse and 15 μg of siPDL1 per mouse) for 8 hours to check the activation of the STING pathway and intratumorally injected with cGAMP, cGAMP-siNC@GalNPs, cGAMP-siNC@NPs, cGAMP-siPDL1@NPs, and cGAMP-siPDL1@GalNPs for 24 hours (25 μl; 15 μg cGAMP per mouse, 15 μg of siPDL1 per mouse, and 15 μg of siNC per mouse) to check the expression of PD-L1. For the laser irradiation groups, the B16F10 tumors or cancer cells received laser irradiation (660 nm, 5 min, 100 mW/cm^2^) at 6 hours after drug administration. Last, the tumors or B16F10 cells were collected to detect PD-L1 levels at 18 hours after laser irradiation. The tumors were collected after specified time, homogenized in RIPA lysis buffer (catalog no. P0013B, Beyotime) and centrifuged (12,000 rpm, 10 min) to obtain protein solutions. The protein concentration was measured using a BCA protein assay kit (catalog no. P0012S, Beyotime). The samples (20 μg total protein) were separated on 7.5% SDS polyacrylamide gels (Epizyme, catalog no. PG111). The separated protein bands were transferred onto a PVDF membrane (220 mA, 2 hours). The PVDF membranes were soaked in 5% BSA for 2 hours and then washed with TBST. Next, the PVDF membranes were incubated with primary antibodies P^Ser396^-IRF3 (1:1000), IRF3 (1:1000), P^Ser172^-TBK1 (1:1000), TBK1 (1:1000), PD-L1 (1:1000), and β-actin (1:2000) at 4°C for 12 hours and washed with TBST. Last, the PVDF membranes were incubated with a secondary antibody (1:50,000) for 1 hour and washed with TBST. Last, the PVDF membranes were detected using an eBLOT touch imager (eBLOT, China). Similarly, the B16F10 cancer cells were exposed to siNC@GalNPs, siNC@NPs, cGAMP, siPDL1@NPs, and siPDL1@GalNPs (siPDL1 = 10 μg/ml, siNC = 10 μg/ml) for 24 hours to analyze PD-L1 protein expression by WB.

In the qPCR experiments, the B16F10 tumor–bearing mice were intratumorally injected with cGAMP, cGAMP-siPDL1@NPs, and cGAMP-siPDL1@GalNPs for 8 hours (25 μl; 15 μg of cGAMP per mouse and 15 μg of siPDL1 per mouse) (*n* = 4) to check *Ifnb1* and *Cxcl10* expression level and intratumorally injected with cGAMP-siNC@GalNPs, cGAMP-siNC@NPs, cGAMP, cGAMP-siPDL1@NPs, and cGAMP-siPDL1@GalNPs for 24 hours (25 μl; 15 μg cGAMP per mouse, 15 μg of siPDL1 per mouse, and 15 μg of siNC per mouse) (*n* = 3) to check the *PD-L1* expression level. The tumors were collected. The total RNAs were extracted using the FastPure Cell/Tissue Total RNA Isolation Kit V2 (catalog no. RC112, Vazyme) and reversely transcribed into cDNA using HiScript III RT SuperMix for qPCR (+gDNA wiper) (catalog no. R323, Vazyme). The obtained cDNA was amplified with ChamQ SYBR color qPCR Master Mix (catalog no. Q411-02/03, Vazyme). Similarly, the B16F10 cancer cells were exposed to cGAMP-siNC@GalNPs, cGAMP-siNC@NPs, cGAMP, cGAMP-siPDL1@NPs, and cGAMP-siPDL1@GalNPs (cGAMP = 100 ng/ml, siPDL1 = 10 μg/ml, siNC = 10 μg/ml) (*n* = 3) to analyze *PD-L1* by qPCR. mRNA levels were normalized against the gene *Acta*.

### Cytokines in tumors and blood measured by an ELISA kit

B16F10 melanoma cancer cells (1 × 10^6^ cells per mouse, 100 μl) were subcutaneously injected into the flank of C57BL/6 mice (female, 6 to 8 weeks) to establish tumor models. When tumor grew to 50 to 100 mm^3^, the mice were randomly assigned into five groups (*n* = 3) and intratumorally injected with cGAMP + siPDL1, cGAMP-siNC@NPs, cGAMP-siPDL1@NPs, and cGAMP-siPDL1@GalNPs (25 μl, containing 15 μg of cGAMP and 15 μg of siPDL1 or siNC). At 48 hours, the tumors and blood were collected, and relevant cytokines (IL-6, TNF-α, IFN-α, and IFN-γ) were detected using an ELISA kit. For checking intratumoral cytokines, the tumors were homogenized in PBS buffer (200 mg/ml) and centrifuged to obtain the supernatant (12,000 rpm, 5 min).

### In vitro and in vivo imaging system

The B16F10-Luc cells (40,000 cells per well) were seeded in 12-well plates for 12 hours and exposed to cGAMP-siLuc@GalNPs with different N/P ratios for 24 hours (cGAMP = 100 ng/ml, siLuc = 10 μg/ml). After washing with PBS buffer three times, 1 ml of fresh culture medium containing d-luciferin potassium salt was added to each well, and the luciferase protein was imaged using IVIS (IVIS Lumina II, PerkinElmer, USA).

Based on the previous method, we established the melanoma models. On reaching ~100 mm^3^, two groups of B16F10 tumor–bearing mice were randomly divided (*n* = 3). Then, the tumors were injected intratumorally with cGAMP-siPDL1@NPs and cGAMP-siPDL1@GalNPs (25 μl, containing 10 μg of cGAMP and 10 μg of siPDL1). The fluorescence images in vivo were obtained at 1, 6, 24, 72, and 96 hours postinjection using IVIS (IVIS Lumina II, PerkinElmer, USA).

The 4T1-Luc cells (1 × 10^6^ cells per mouse, 100 μl) were injected orthotopically into the right fourth mammary fat pad of the female Balb/c mice. When the orthotopic breast tumors reached around 300 mm^3^, the tumors were injected intratumorally with Cy5 siPDL1 and cGAMP (Cy5 siPDL1 + cGAMP), cGAMP-Cy5 siPDL1@NPs, and cGAMP-Cy5 siPDL1@GalNPs (25 μl, containing 10 μg of cGAMP and 10 μg of Cy5 siPDL1). Then, the mice were imaged using IVIS (IVIS Lumina II, PerkinElmer, USA) at 1, 6, 12, and 24 h post–drug injection.

### Antitumor efficacy of primary and distant B16F10 tumors

A total of 1 × 10^6^ B16F10 cells were subcutaneously injected into the left flank of female C57BL/6 mice to construct B16F10 primary tumor models. To form the second tumor (distant tumor), B16F10 tumor cells (5 × 10^5^ cells per mouse, 100 μl) were subcutaneously injected into the right flank when the primary tumor grew to 80 to 100 mm^3^. The mice were randomly divided into 10 groups (*n* = 6) and received the following treatments by intratumorally drug injection: cGAMP + siPDL1, siPDL1@GalNPs, cGAMP-siNC@GalNP, cGAMP-siPDL1@NPs, cGAMP-siPDL1@GalNPs, siNC@GalNPs + L, cGAMP-siNC@GalNPs + L, cGAMP-siPDL1@NPs + L, and cGAMP-siPDL1@GalNPs + L (25 μl; 15 μg of cGAMP per mouse, 15 μg of siPDL1 per mouse, and 15 μg of siNC per mouse). At 6 hours after drug injection, the primary B16F10 tumors in the +L groups received laser irradiation (660 nm, 5 min, 100 mW/cm^2^). The primary B16F10 melanoma tumors were obtained on day 2 for IHC staining of cleaved caspase-3 and IF staining of Ki67 and TUNEL. The tumor volumes and body weight of all mice were recorded every other day. Tumor volumes of mice were calculated as the equation: tumor volume = 0.5 × width^2^ × length.

### Antitumor efficacy of orthotopic breast tumors and the spontaneous metastasis

The 4T1-Luc cells (1 × 10^6^ cells per mouse, 100 μl) were injected orthotopically into the right fourth mammary fat pad of the female Balb/c mice. When the orthotopic tumor grew to 80 to 100 mm^3^, we randomly divided the mice into 10 groups (*n* = 6), and the mice received the following treatments by intratumorally drug injection: cGAMP + siPDL1, siPDL1@GalNP, cGAMP-siNC@GalNPs, cGAMP-siPDL1@NPs, cGAMP-siPDL1@GalNPs, siNC@GalNPs + L, cGAMP-siNC@GalNPs + L, cGAMP-siPDL1@NPs + L, and cGAMP-siPDL1@GalNPs + L (25 μl; 15 μg of cGAMP per mouse and 15 μg of siPDL1/siNC per mouse). At 6 hours after drug injection, the orthotopic 4T1-Luc breast tumors in the +L groups received laser irradiation (5 min, 660 nm, 100 mW/cm^2^). The volumes of orthotopic breast tumors and the body weight of all mice were recorded every other day. On day 20, the orthotopic 4T1-Luc tumor was surgically removed, and the spontaneous lung metastasis was detected using IVIS (IVIS Lumina II, PerkinElmer, USA). The orthotopic breast tumors were collected on day 2 for IHC staining of cleaved caspase-3 and IF staining of Ki67 and TUNEL. When animals were euthanized, we counted lung metastasis nodules and collected lung tissues for H&E staining.

### Flow cytometric analysis of the B16F10 tumors

Cy5 siPDL1 was used to prepare cGAMP-Cy5 siPDL1@NPs and cGAMP-Cy5 siPDL1@GalNPs for studying cellular uptake in vivo. B16F10 melanoma tumors were harvested at 6 and 24 hours after administration of nanocarriers (15 μg of cGAMP per mouse and Cy5 siPDL1 15 μg per mouse). Tumors were digested in RPMI 1640 media containing deoxyribonuclease I (catalog no. 18047019, Invitrogen) and collagenase IV (catalog no. 17104019, Gibco) for 30 min at 37°C to obtained a single-cell suspension, which was detected using a flow cytometer (NovoCyte, Agilent, USA).

To study the immune responses, subcutaneous B16F10 tumor–bearing mice received the following treatments by intratumorally drug injection: cGAMP + siPDL1, siPDL1@GalNPs, cGAMP-siPDL1@NPs, cGAMP-siNC@GalNPs, cGAMP-siPDL1@GalNPs, siNC@GalNPs + L, cGAMP-siPDL1@NPs + L, cGAMP-siNC@GalNPs + L, and cGAMP-siPDL1@GalNPs + L. At 6 hours after drug injection, the tumors in the +L groups received laser irradiation (5 min, 660 nm, 100 mW/cm^2^). Forty-eight hours after injection, the tumors were collected and digested in RPMI 1640 media containing deoxyribonuclease I (catalog no. 18047019, Invitrogen) and collagenase IV (catalog no. 17104019, Gibco) for 30 min at 37°C. The solution was filtered with nylon mesh filters to prepare single-cell suspension. The single-cell suspensions were lysed with the red blood cell lysis buffer (catalog no. R1010, Solarbio). The cells were resuspended in staining buffer (catalog no. 00-4222-26, eBioscience) at a concentration of 1 × 10^7^ cells/ml. The cells were stained with several panels of antibodies including the fluorescein isothiocyanate (FITC) anti-mouse CD45, phycoerythrin (PE)–Cy7 anti-mouse CD3e, BV510 anti-mouse CD8a, Brilliant Violent 421 anti-mouse CD8, anti-mouse granzyme B, allophycocyanin anti-mouse IFN-γ, Brilliant Violent 510 anti-mouse CD45, cyanine 5.5 anti-mouse CD3e, FITC anti-mouse CD4, APC anti-mouse CD8a, Brilliant Violent 421 anti-mouse CD25, PE anti-mouse Foxp3, allophycocyanin anti-mouse CD11c, PE/Cy7 anti-mouse CD80, PE anti-mouse CD86, FITC anti-mouse CD11b, PE anti-mouse F4/80, PE/Cy7 anti-mouse CD206, and allophycocyanin anti-mouse CD86. All stained cells were measured using a flow cytometer (Fortessa X20, BD Biosciences, USA). Gating strategies of immune cells in the TME are shown in fig. S40.

### In vivo safety evaluation

4T1 breast tumor–burdened female Balb/c mice were treated intratumorally as follows: cGAMP + siPDL1, cGAMP-siNC@NPs, cGAMP-siPDL1@NPs, and cGAMP-siPDL1@GalNPs (25 μl; 15 μg of cGAMP per mouse, 15 μg of siPDL1 per mouse, and 15 μg of siNC per mouse). Forty-eight hours after injection, mice were euthanized, and their blood, heart, liver, spleen, lung, and kidney were collected. The organs were fixed in 4% PFA and stained with H&E. Obtained H&E sections were examined on a pathological section scanner (MIDI, Pannoramic, USA). Blood was taken for biochemical analysis and blood routine examination.

### Statistical analysis

GraphPad Prism 9 software was used for statistical analysis. Data are presented as means ± SD. The difference between two groups was evaluated using an unpaired Student’s *t* test. A Kaplan-Meier survival analysis with a Mantel-Cox test was used to compare differences in overall survival between groups of mice. One-way analysis of variance (ANOVA) with Tukey’s multiple comparisons was used to compare more than two groups. A Kruskal-Wallis test is a nonparametric alternative to the one-way ANOVA test for multiple comparisons. Specific statistical methods are indicated in the figure legends.

## Supplementary Materials

### This PDF file includes:

Supplementary Text Figs. S1 to S40 Table S1 References
