# Supplementary material for: Tumor-targeting nanocarriers amplified immunotherapy of cold tumors by STING activation and inhibiting immune evasion
Source: Sci Adv. 2025 Jun 27;11(26):eadr1728. doi: 10.1126/sciadv.adr1728 (PMC12204131; doi:10.1126/sciadv.adr1728)
Supplement: Supplementary file 1 — Supplementary Text Figs. S1 to S40 Table S1 References [file sciadv.adr1728_sm.pdf]

Science Advances
AAAS

Supplementary Materials for

Tumor-targeting nanocarriers amplified immunotherapy of cold tumors by STING activation and inhibiting immune evasion

Jinhua Zhao *et al.*

Corresponding author: Peng Mi, mi@scu.edu.cn

Sci. Adv. **11**, eadr1728 (2025)
DOI: 10.1126/sciadv.adr1728

This PDF file includes:

Supplementary Text
Figs. S1 to S40
Table S1
References

Supplementary Text

1. Materials and Methods

1.1 Main materials, cells and animals

All chemicals were purchased from Bide Pharmatech Ltd unless otherwise indicated. Pyropheophorbide-alpha (PPa) was purchased from Shanghai Yuanye Bio-Technology Co., Ltd (Shanghai, China). Dimethyl-3,3'-dithiobispropionimidate/2HCl (DTBP/HCl) was purchased from Merk. 2'3'-cGAMP (Cat#B8362) were bought from APExBIO. Mouse ELISA kit of TNF-α (Cat#abs520010) was purchased from Absin Biotech Co., Ltd (Shanghai, China). Mouse ELISA kits for IL-6 (Cat# 1310602), IFN-γ (Cat#1210002) were purchased from Dakewe Biotech Co., Ltd (Shenzhen, China). The mouse ELISA kit of IFN-α (Cat#EM1146) was purchased from Wuhan Fine Biotech Co., Ltd (Wuhan, China). The antibodies used in this study are listed in **Table S1**. PD-L1 siRNA (siPDL1) (sense 5'-3': GAGGUAAUCUGGACAAACATT; antisense 5'-3': UGUUUGUCCAGAUUACCUCTT), negative control siRNA (siNC), firefly luciferase siRNA (siLuc) (sense 5'-3': ATTACACCCGAGGGGGATGA; antisense 5'-3': TCTCACACACAGTTCGCCTC) and DNA primers were purchased from Sangon Biotech (Shanghai) Co., Ltd (Shanghai, China) (*83*). The DNA primers were listed here: mouse *Ifnb1*: ATGAGTGGTGGTTGCAGGC, TGACCTTTCAAATGCAGTAGATTCA; mouse *Cxcl10*: GGAGTGAAGCCACGCACAC, ATGGAGAGAGGCTCTCTGCTGT; mouse *Actb*: ACACCCGCCACCAGTTCGC, ATGGGGTACTTCAGGGTCAGGATA; mouse *PD-L1*: TGCGGACTACAAGCGAATCACG, CTCAGCTTCTGGATAACCCTCG. B16F10, B16F10-Luc, 4T1 and 4T1-Luc cells were maintained in Roswell Park Memorial Institute-1640 (RPMI 1640) medium (HyClone, Cat#SH30096.02) with 10% FBS (Gemini, Cat#900-108) and 1% penicillin/streptomycin (Gibco, Cat#15070063) at 37°C under a 5% CO_2 incubation. C57BL/6 mice (female, 18-22 g) and Balb/c mice (female, 18-20 g) were bought from the Experimental Animal Centre of Sichuan University (Chengdu, China).

1.2 Synthesis of PPa-NH_2

PPa-NH_2 was synthesized by referring previous protocols (*84, 85*). As the synthetic route shown in Fig. S1A, the pyrropheophorbide-alpha (PPa, 1 eqv, 200 mg, 0.37 mmol), 1-ethyl-3-(3-dimethylaminopropyl) carbodiimide hydrochloride (EDCI, 2 eqv, 141.86 mg, 0.74 mmol) and N-hydroxy succinimide (NHS, 2 eqv, 85.17 mg, 0.74 mmol) were dissolved in dry dichloromethane (DCM, 10 mL) to stir at 37°C for 4 h under argon gas. Then, tert-butyl (2-aminoethyl) carbamate (1.5 eqv, 89.9 mg, 0.56 mmol) and triethylamine (TEA, 20 μL) were added to react for 24 h. The reacted solution was diluted with DCM and washed with brine, while the organic phase was separated, evaporated and purified by SiO_2 column (DCM/MeOH = 98:2) to collect product **PPa-NHBoc**. The **PPa-NHBoc** (100 mg, 0.15 mmol) was dissolved in DCM (5 mL) and then added trifluoroacetic acid (TFA, 2 ml) to react for 4 h at 37°C. Then, sodium bicarbonate solution was added to adjust the pH, while the organic phase was separated and evaporated to obtained **PPa-NH_2**. The **PPa-NHBoc** and **PPa-NH_2** were confirmed with ^1H NMR as shown in Fig. S2-3 (Advance 400M, Bruker, Germany).

1.3 Synthesis of thiol-galactose (Gal-SH)

The **Gal-SH** was synthesized as shown in Fig. S1B (*86*). The 11-mercaptoundecanoic acid (1 eqv, 106 mg, 0.99 mmol), EDCI (1.1 eqv, 210 mg, 1.1 mmol) and NHS (1.1 eqv, 127 mg 1.1 mmol) were dissolved in dry dimethyl sulfoxide (DMSO, 10 mL) to stir at 37°C for 4 h under Argon gas, and then added with β-D-Galactosamine (1.1 eqv, 216 mg, 1.1 mmol) and TEA (500 μL) to react for 24 h. The product was obtained by precipitation with Milli-Q water (20 mL), treated with 1 M NaOH solution (10 mL) and 1 M HCl (pH 5.0) subsequently, washed and lyophilized to get **Gal-SH**, while it was characterized by ^1H NMR as shown in Fig. S4 (Advance 400M, Bruker, Germany).

1.4 Synthesis of pheophorbide a-poly(β-benzyl-L-aspartate) (PPa-PBLA)

The **PPa-PBLA** was obtained by the ring-opening polymerization of benzyl-L-aspartate N-carboxy anhydride (BLA-NCA) with **PPa-NH_2**. The synthetic route has shown in Fig. S1C. Briefly, **PPa-NH_2** (20 mg, 34.68 μmol) dissolved in 1 mL dry N, N-dimethylformamide (DMF) was reacted with BLA-NCA (432 mg, 1.73 mmol) dissolved in dry DCM/DMF (9:1 v/v, 10 mL) at 35°C under stirring and argon gas protection for 72 h. Then, the products were obtained

by precipitating in diethyl ether three times, and freeze-dried with benzene. The degree of polymerization (DP) of PPa-PBLA was 43 as checked by ^{1}H NMR in Fig. S5 (Advance 400M, Bruker, Germany).

1.5 Synthesis of PEG-PBLA-PPa and Gal-PEG-PBLA- PPa

The synthetic route of **PEG-PBLA-PPa** and **Gal-PEG-PBLA-PPa** has shown in Fig. S1C. PPa-PBLA (200 mg) and methoxy poly (ethylene glycol) succinimidyl ester (2 K, MeO-PEG-NHS) (1.2 eqv, 54 mg) were dissolved in dry DCM/DMF (9:1 v/v, 15 mL) and mixed to react at 30°C under stirring, argon gas protection and dark condition for 24 h. Then, Added the reaction solution to cold diethyl ether, filtrated to obtain precipitation, washed with cold ether and dried under vacuum to obtain **PEG-PBLA-PPa**. Similarly, PPa-PBLA (200 mg), **Gla-SH** (1.2 eqv, 5 mg) and maleimide-PEG-NHS ester (2 K, NHS-PEG-Mal) (1.2 eqv, 54 mg) in dry DCM/DMF (9:1 v/v, 15 mL) at 30°C under stirring and argon gas protection in dark condition for 24 h. Then, the reaction solution was precipitated with cold diethyl ether as the same way and dried under vacuum to obtain **Gal-PEG-PBLA-PPa**.

1.6 Synthesis of PEG-PAsp(DET)-PPa and Gal-PEG-PAsp(DET)-PPa

As shown in Fig. S1C, **PEG-PAsp(DET)-PPa** and **Gal-PEG-PAsp(DET)-PPa** were synthesized through the aminolysis reaction of **PEG-PBLA-PPa** and **Gal-PEG-PBLA-PPa** with diethylenetriamine (DET), respectively. **Gal-PEG-PBLA-PPa** polymers (150 mg) dissolved in 15 mL dry N-methylpyrrolidone (NMP) were dropped into DET (2 mL, 50 eqv. to benzyl groups of **PPa-PBLA**) dissolved in dry NMP to react at 4°C under stirring and argon gas protection for 4 h. The products were added with cold HCl solution (5 M, 50 mL) and dialyzed against 0.01 M HCl and Milli-Q water at 4°C for several times, and finally lyophilized to obtain **Gal-PEG-PAsp(DET)-PPa**. In the same method, the **PEG-PAsp(DET)-PPa** was synthesized. The **PEG-PAsp(DET)-PPa** and **Gal-PEG-PAsp(DET)-PPa** were characterized by ^{1}H NMR in Fig. S6-7 (Advance 400M, Bruker, Germany)

1.7 Synthesis of PEG-PAsp(DET/MPA)-PPa and Gal-PEG-PAsp(DET/MPA)-PPa

As shown in Fig. S1C, **Gal-PEG-PAsp(DET/MPA)-PPa** was synthesized by reacting the amine groups in DET with DTBP. **Gal-PEG-PAsp(DET)-PPa** (80 mg, 0.3 mmol DET) dissolved in HEPES buffer (100 mM, pH 9) was mixed and reacted with DTBP (34.5 mg, 0.1

mmol) under stirring at 4°C for 2 h. The solution was dialyzed against phosphate buffered saline (PBS, 10 mM, pH 7.4) for 2 h, then PBS (10 mM, pH 6) containing 100 mM dithiothreitol (DTT) for 30 min for disulfide reduction. Finally, it was dialyzed in Milli-Q water for 24 h and lyophilized to collect **Gal-PEG-PAsp(DET/MPA)-PPa**. Similarly, **PEG-PAsp(DET/MPA)-PPa** was also synthesized in the same way. Both **Gal-PEG-PAsp(DET/MPA)-PPa** and **PEG-PAsp(DET/MPA)-PPa** polymers were characterized by ^{1}H NMR in Fig. S8-9 (Advance 400M, Bruker, Germany).

2. Figures

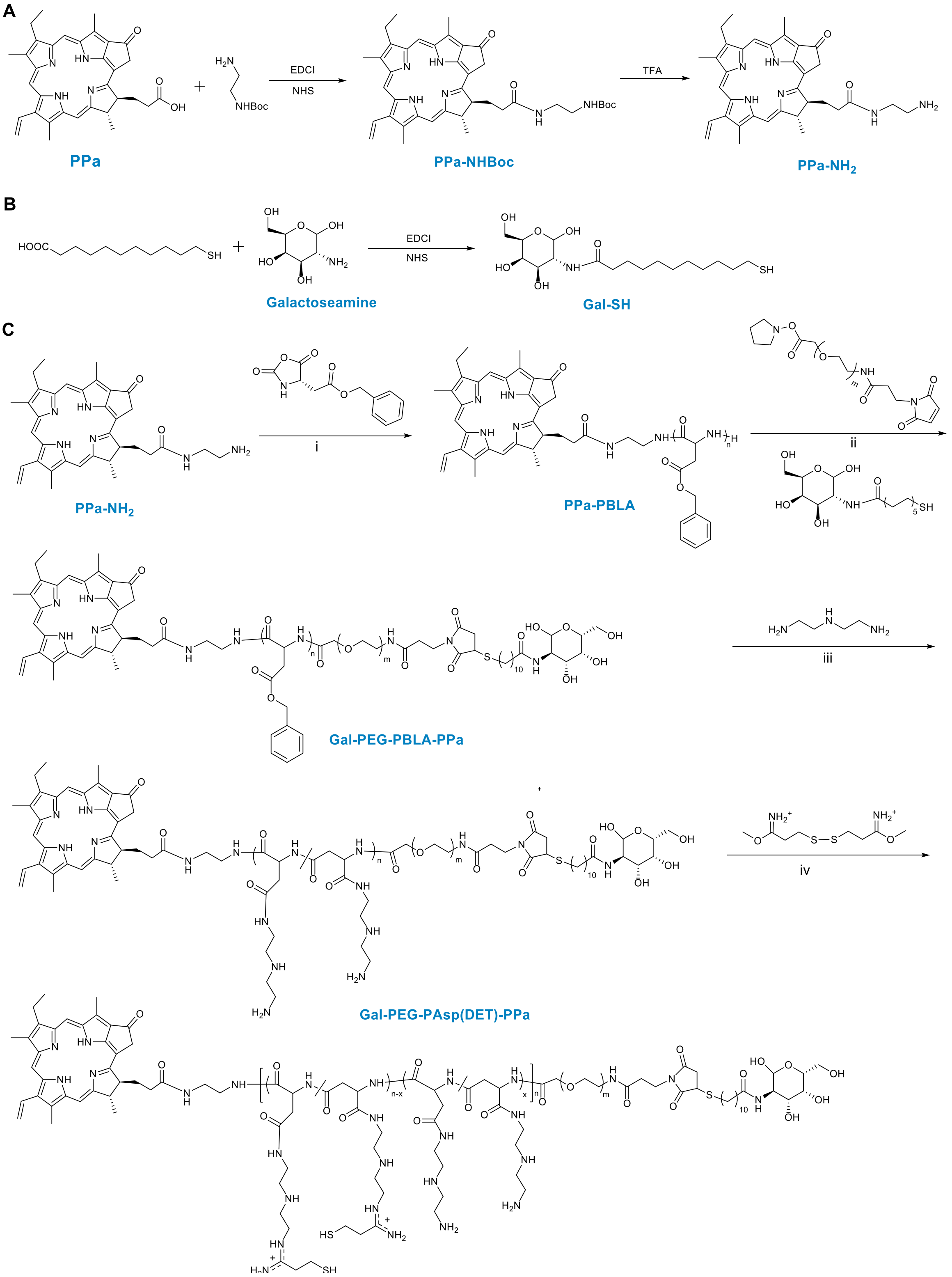

Fig. S1. Synthetic routes for obtaining PPa-NH₂ (A), Gal-SH (B) and Gal-PEG-PAsp(DET/MPA)-PPa (C). (i) dry DCM/DMF (9:1 v/v), Ar_2, 35°C, 3 d. (ii) dry DCM/DMF (9:1 v/v), Ar_2, 30°C, 24 h. (iii) dry NMP, 4°C, Ar_2, 4 h. (iv) HEPES (0.1 mM, pH = 9), 4°C, 2 h.

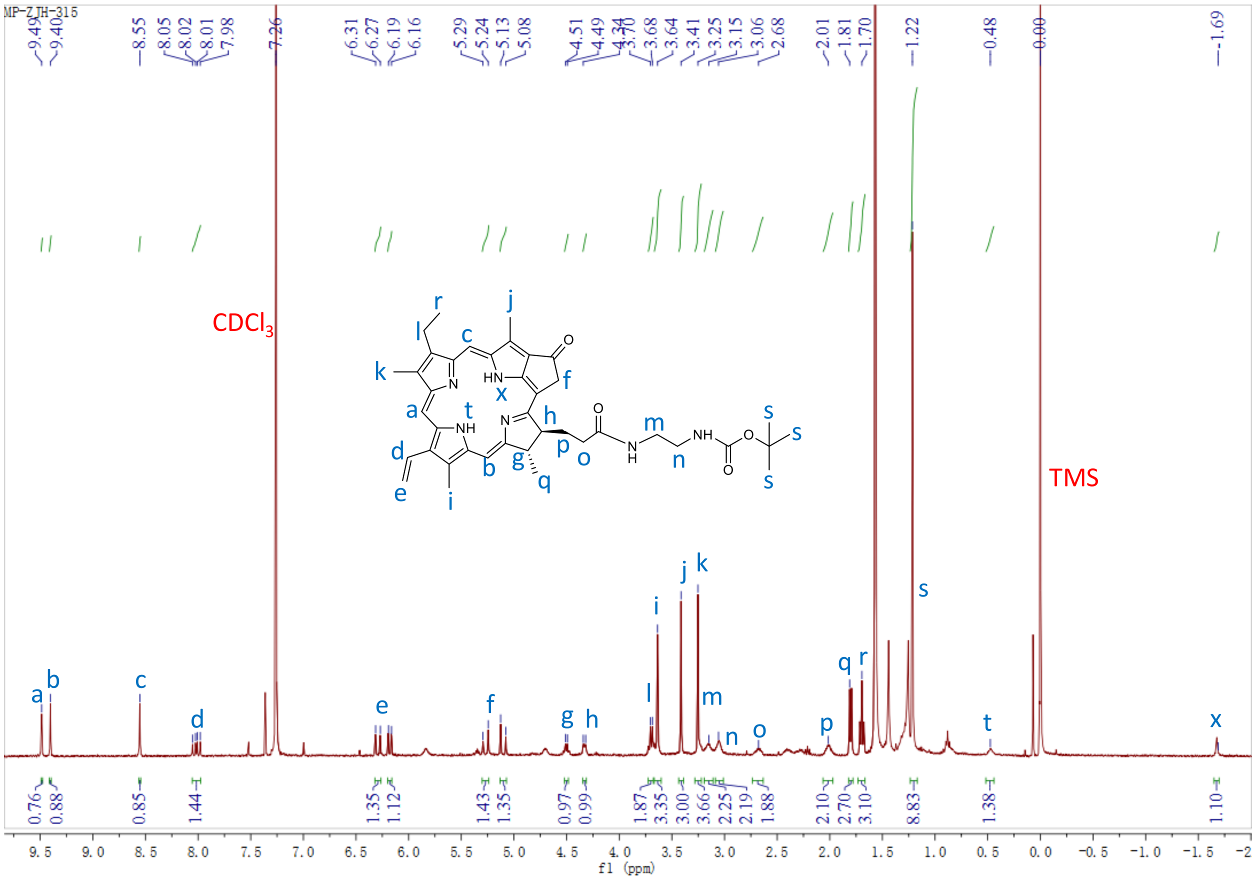

Fig. S2. ^{1}H NMR spectrum (400 MHz) of **PPa-NHBoc** in $CDCl_3$.

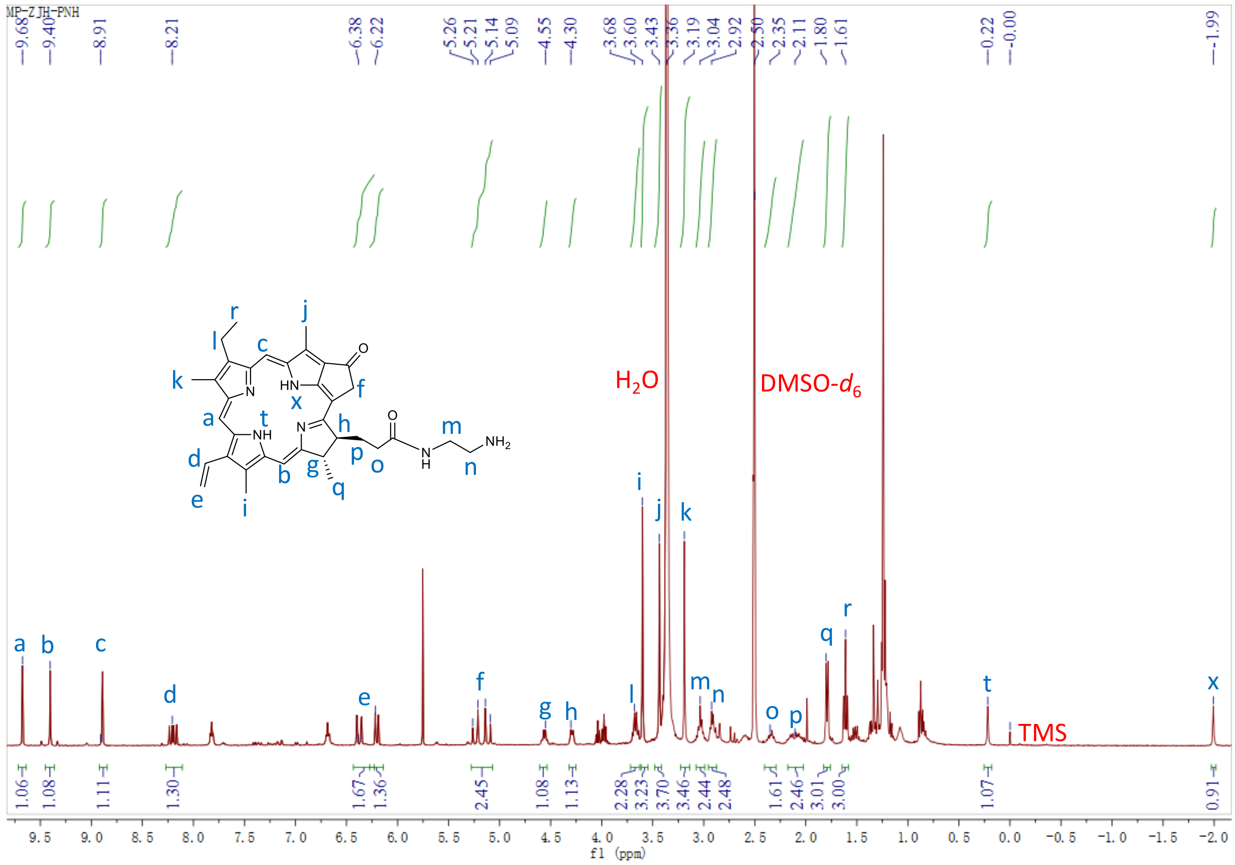

Fig. S3. ^{1}H NMR spectrum (400 MHz) of **PPa-NH$_2$** in DMSO-d_6.

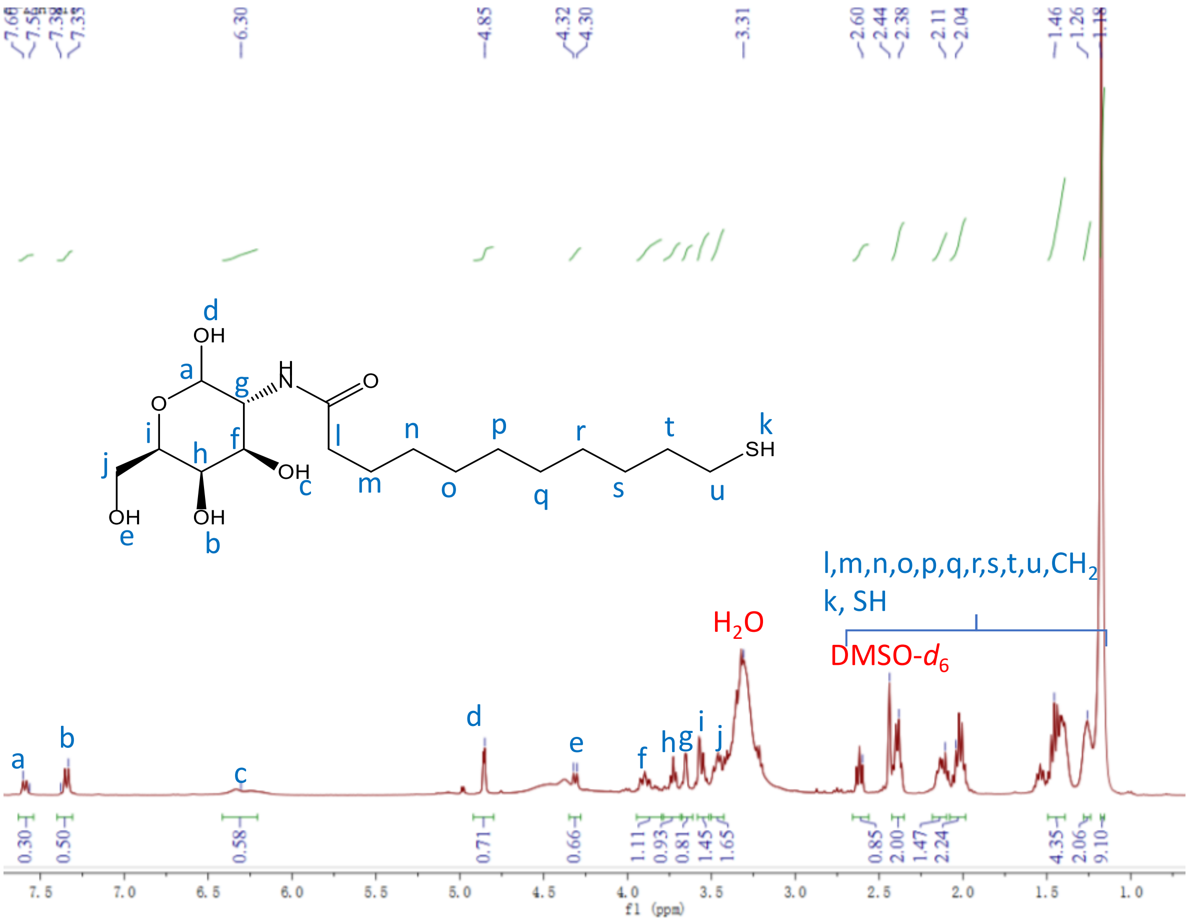

Fig. S4. ^{1}H NMR spectrum (400 MHz) of **Gal-SH** in DMSO-d_6.

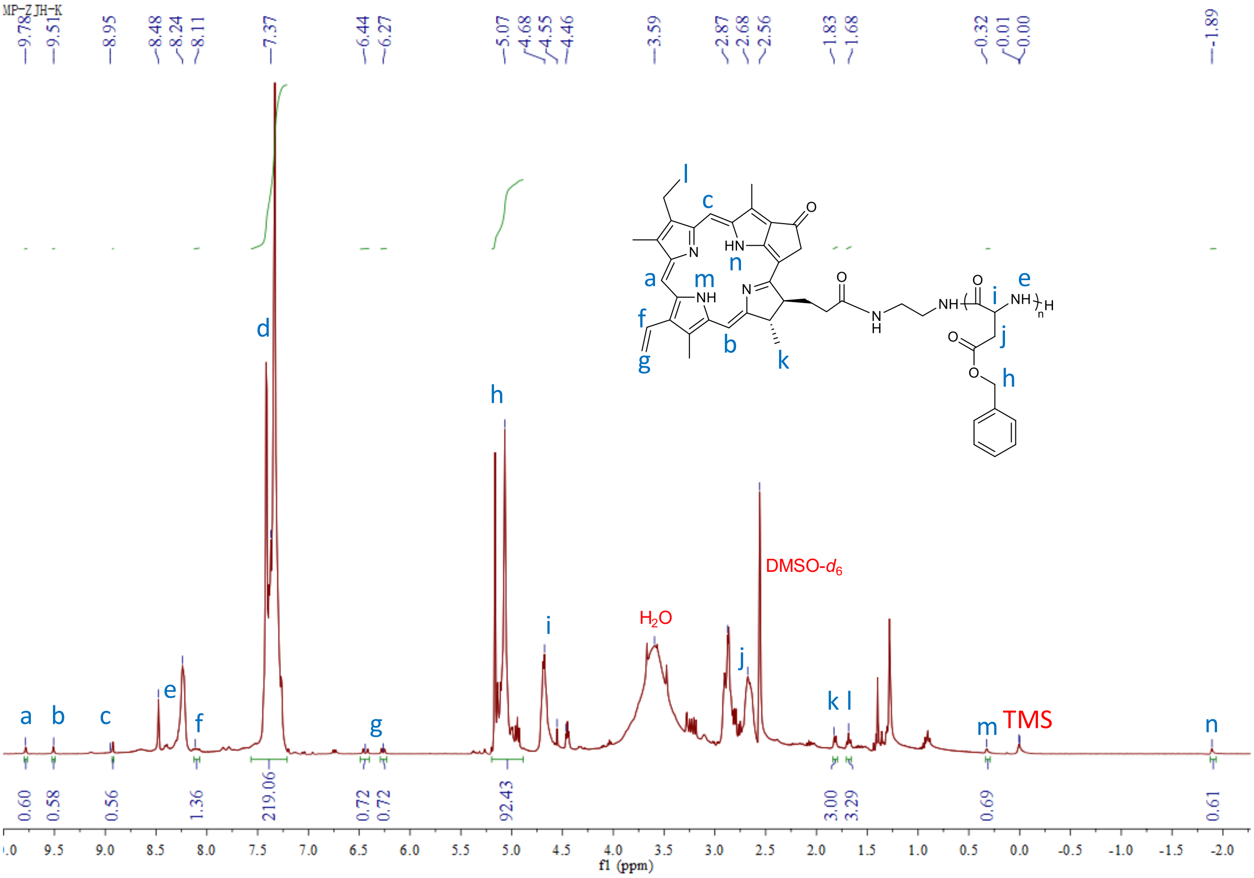

Fig. S5. ^{1}H NMR spectrum (400 MHz) of **PPa-PBLA** in DMSO-d_6.

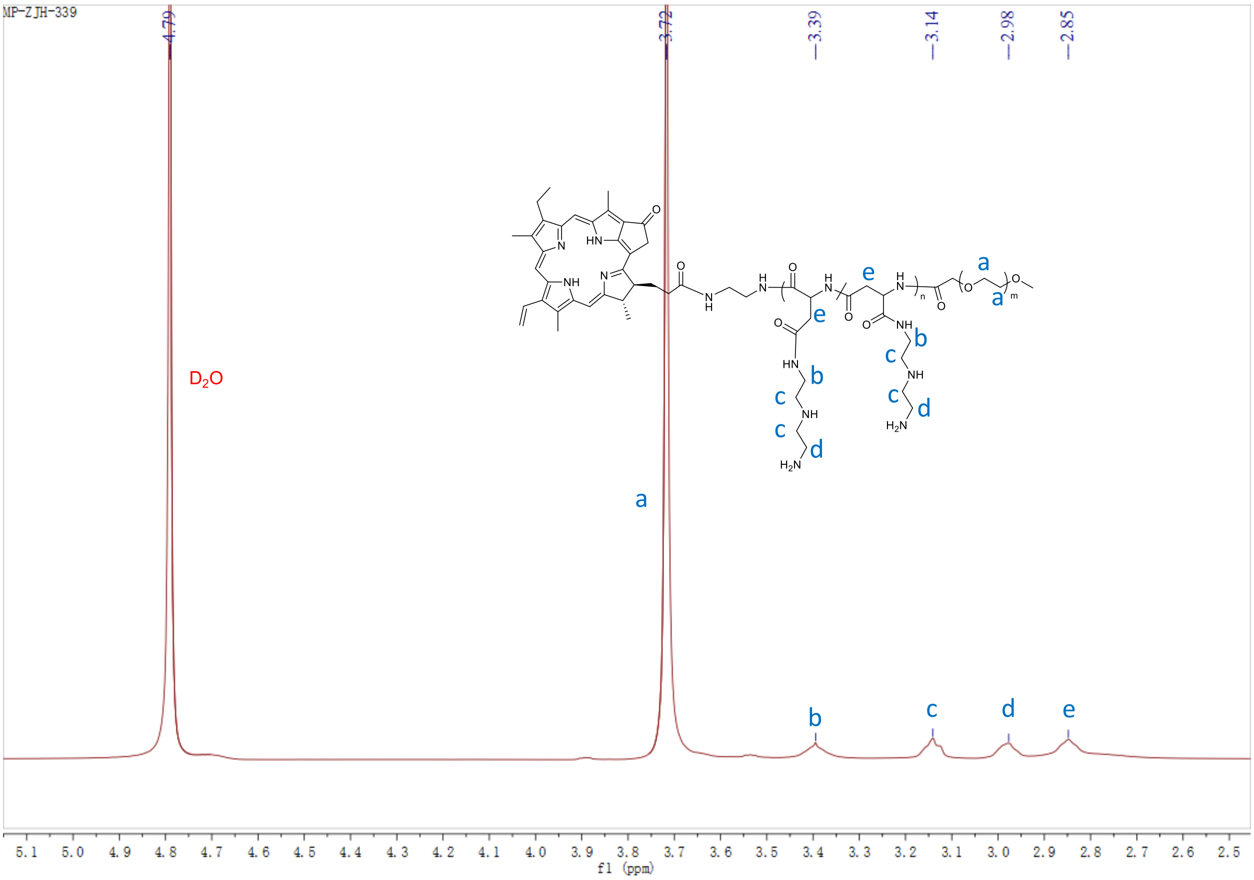

Fig. S6. ^1H NMR spectrum (400 MHz) of **PEG-PAsp(DET)-PPa** in D_2O.

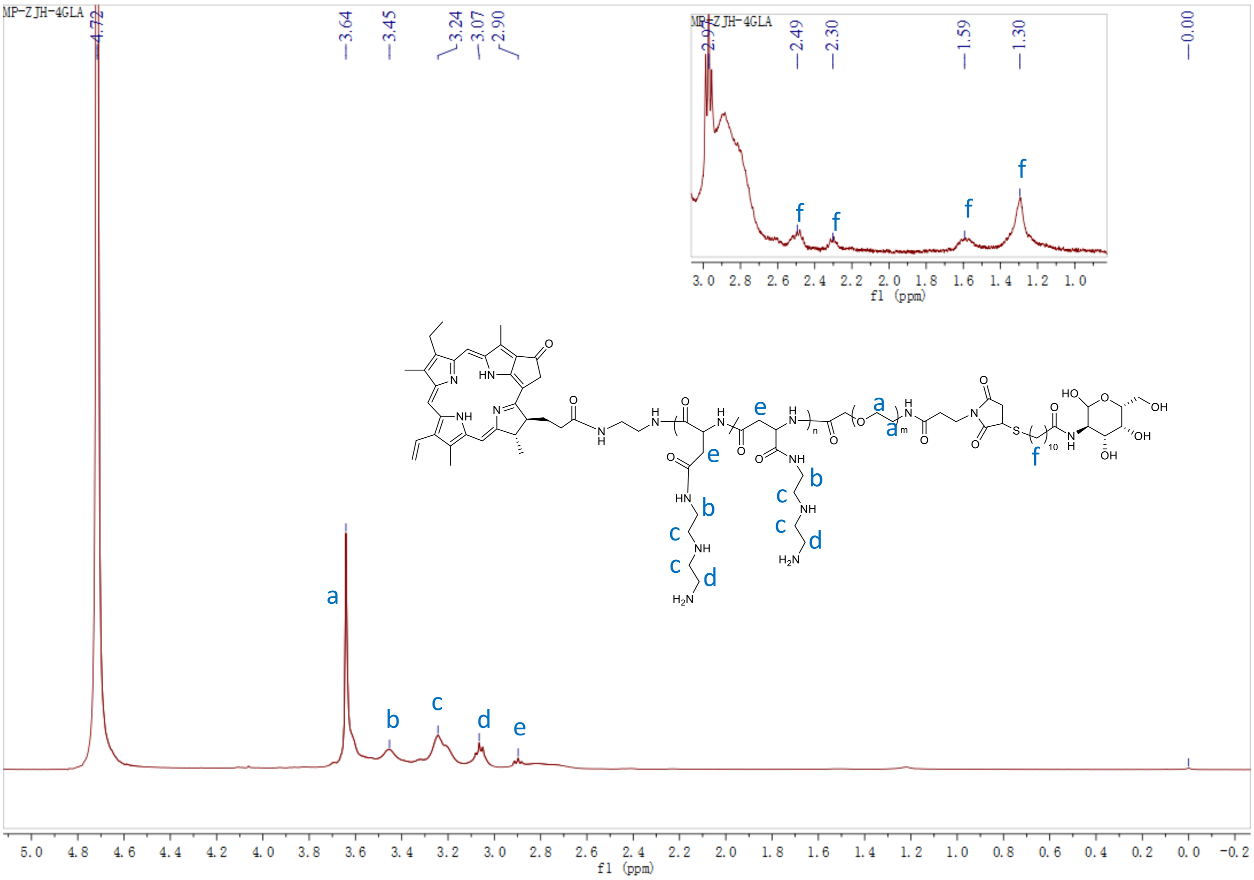

Fig. S7. ^{1}H NMR spectrum (400 MHz) of **Gal-PEG-PAsp(DET)-PPa** in D_2O.

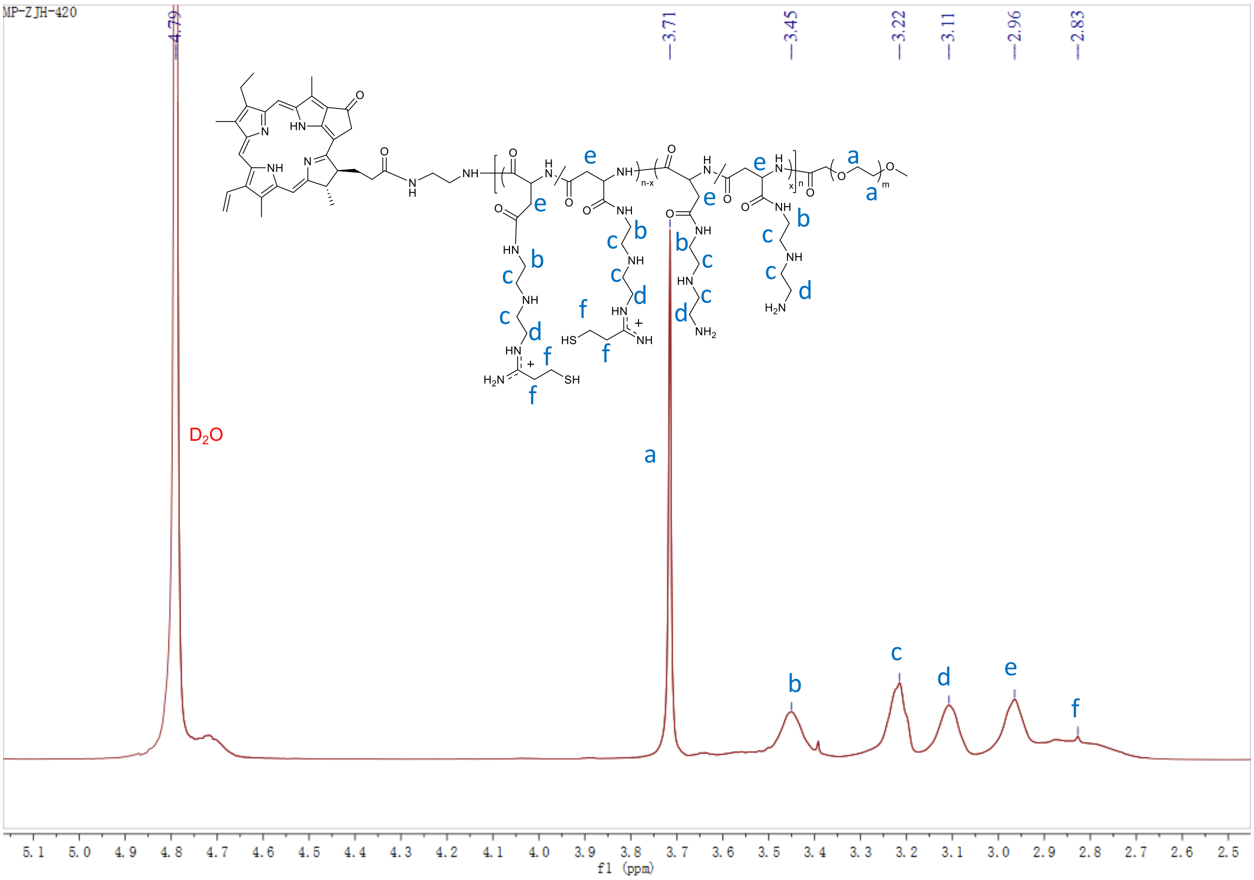

Fig. S8. ^{1}H NMR spectrum (400 MHz) of **PEG-PAsp(DET/MPA)-PPa** in D_2O.

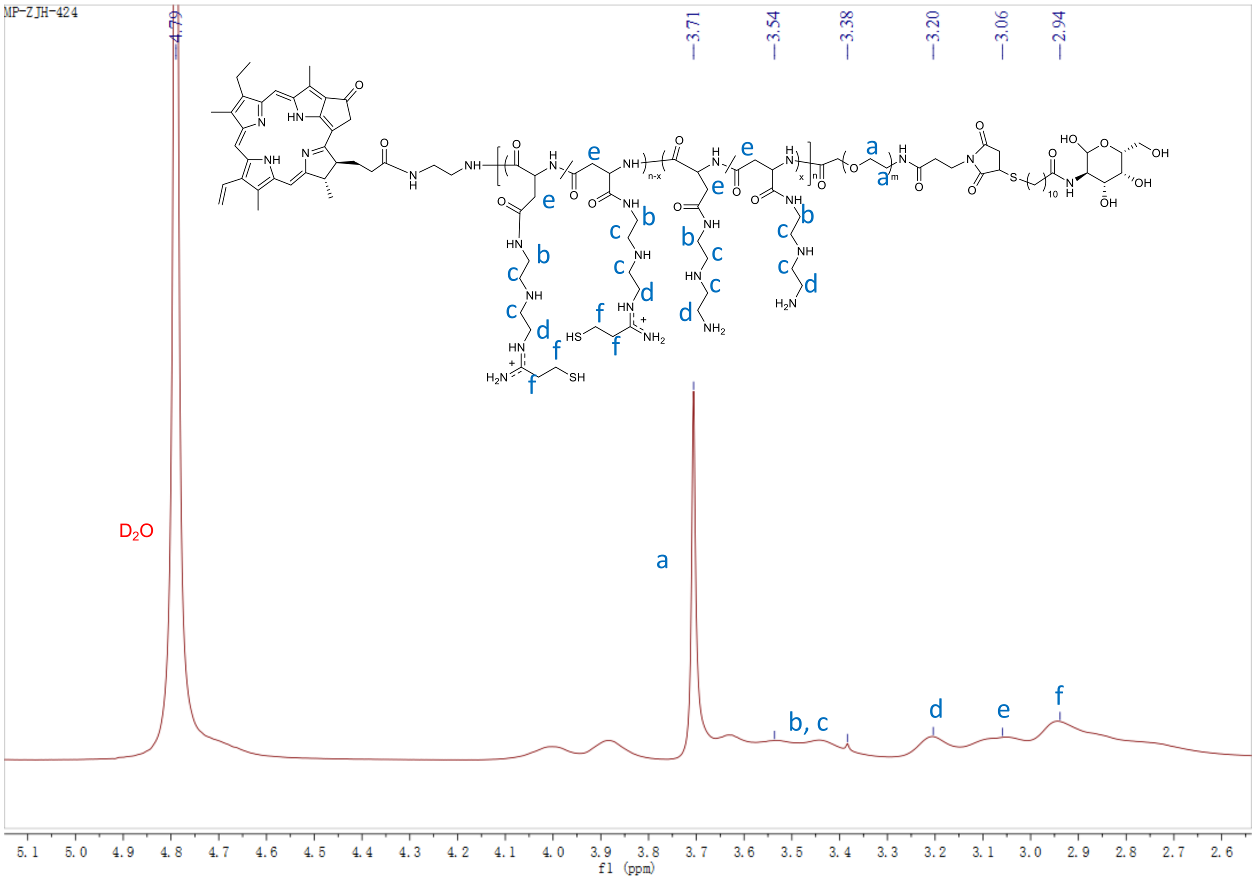

Fig. S9. ^{1}H NMR spectrum (400 MHz) of **Gal-PEG-PAsp(DET/MPA)-PPa** in D_2O.

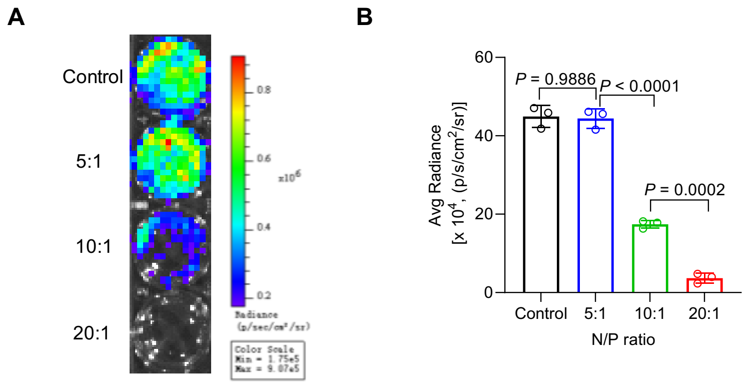

Fig. S10. A. IVIS images show the effects of luciferase silencing in B16F10-Luc cancer cells after adding cGAMP-siLuc@GalNPs with different N/P ratios for 24 h. **B**. Quantitative analysis of the luminescence intensity of luciferase in B16F10-Luc cancer cells. Data were expressed as mean ± s.d. (n = 3). Statistical significance was determined by one-way ANOVA with Tukey's test.

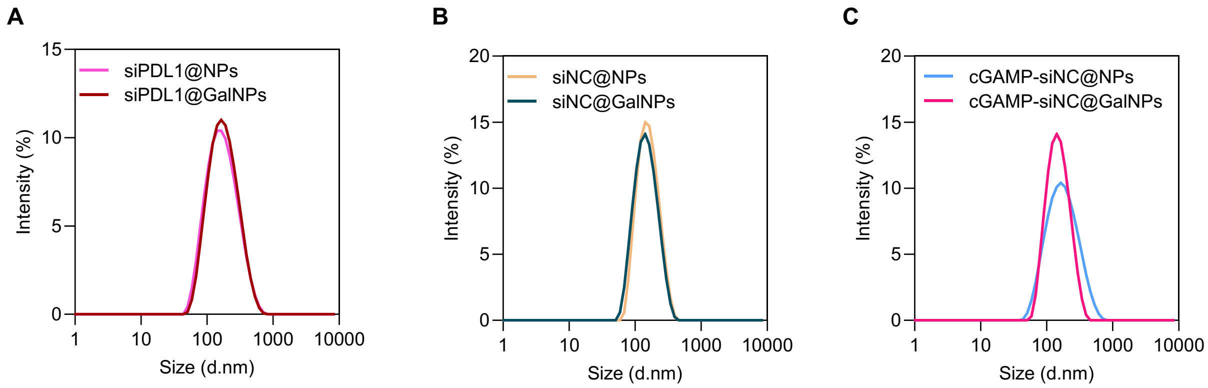

Fig. S11. Size distributions of the control nanocarriers: siPDL1@NPs (**A**), siPDL1@GalNPs (**A**), siNC@NPs (**B**), siNC@GalNPs (**B**), cGAMP-siNC@NPs (**C**) and cGAMP-siNC@GalNPs (**C**).

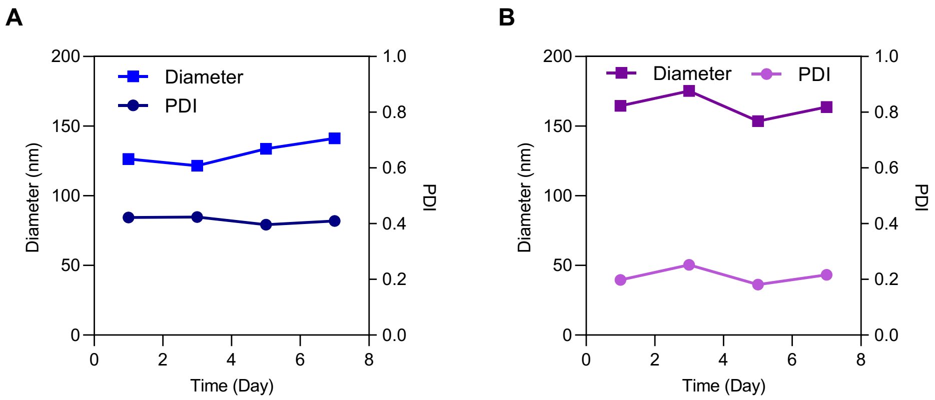

Fig. S12. The stability of nanocarriers. **A**, **B** Diameter and PDI changes of cGAMP-siPDL1@NPs (**A**) and cGAMP-siPDL1@GalNPs (**B**) in cell culture medium (RPMI 1640 + 10%FBS) monitored at different time points.

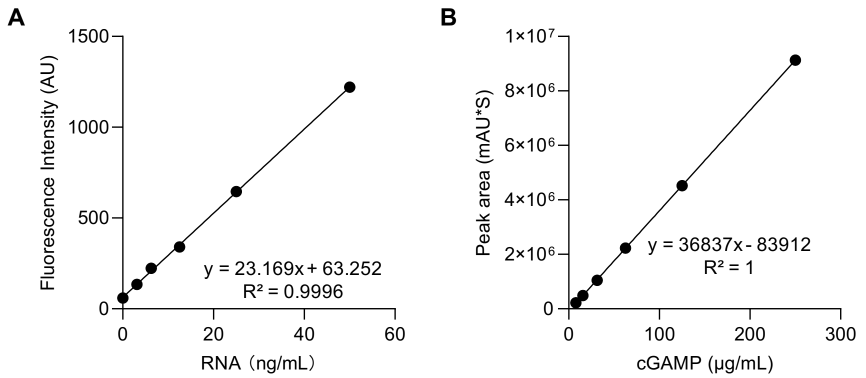

Fig. S13. A. Standard curve obtained by measuring standard ribosomal RNA using RiboGreen RNA reagent. **B.** The HPLC calibration curve of cGAMP.

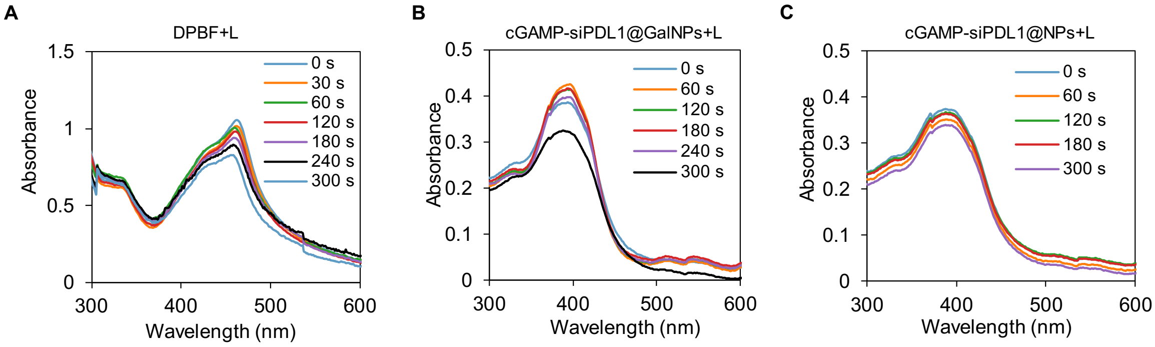

Fig. S14. UV-Vis absorption spectra of DPBF (**A**), cGAMP-siPDL1@GalNPs (**B**) and cGAMP-siPDL1@NPs (**C**) under laser irradiation (660 nm, 100 mW/cm^2).

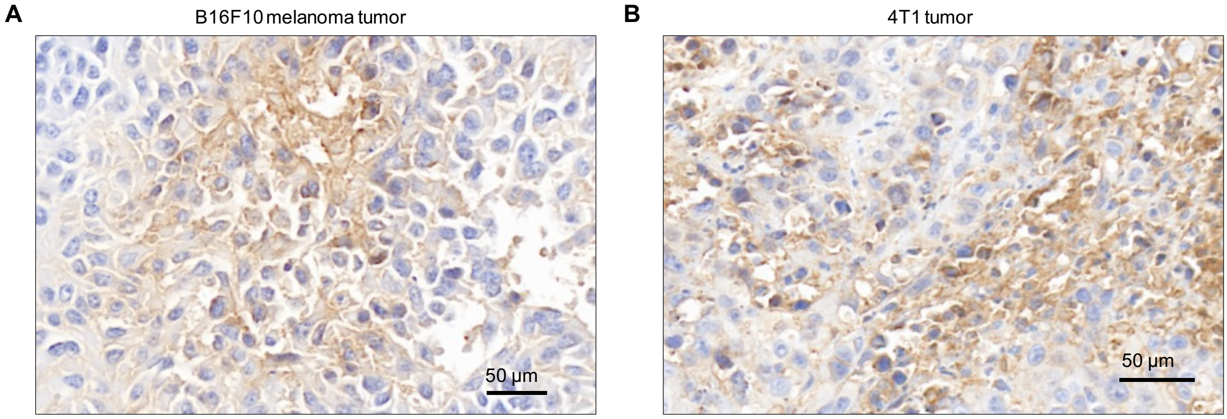

Fig. S15. Immunohistochemical staining of GLUT-1 in B16F10 melanoma tumor (**A**) and 4T1 breast tumor (**B**).

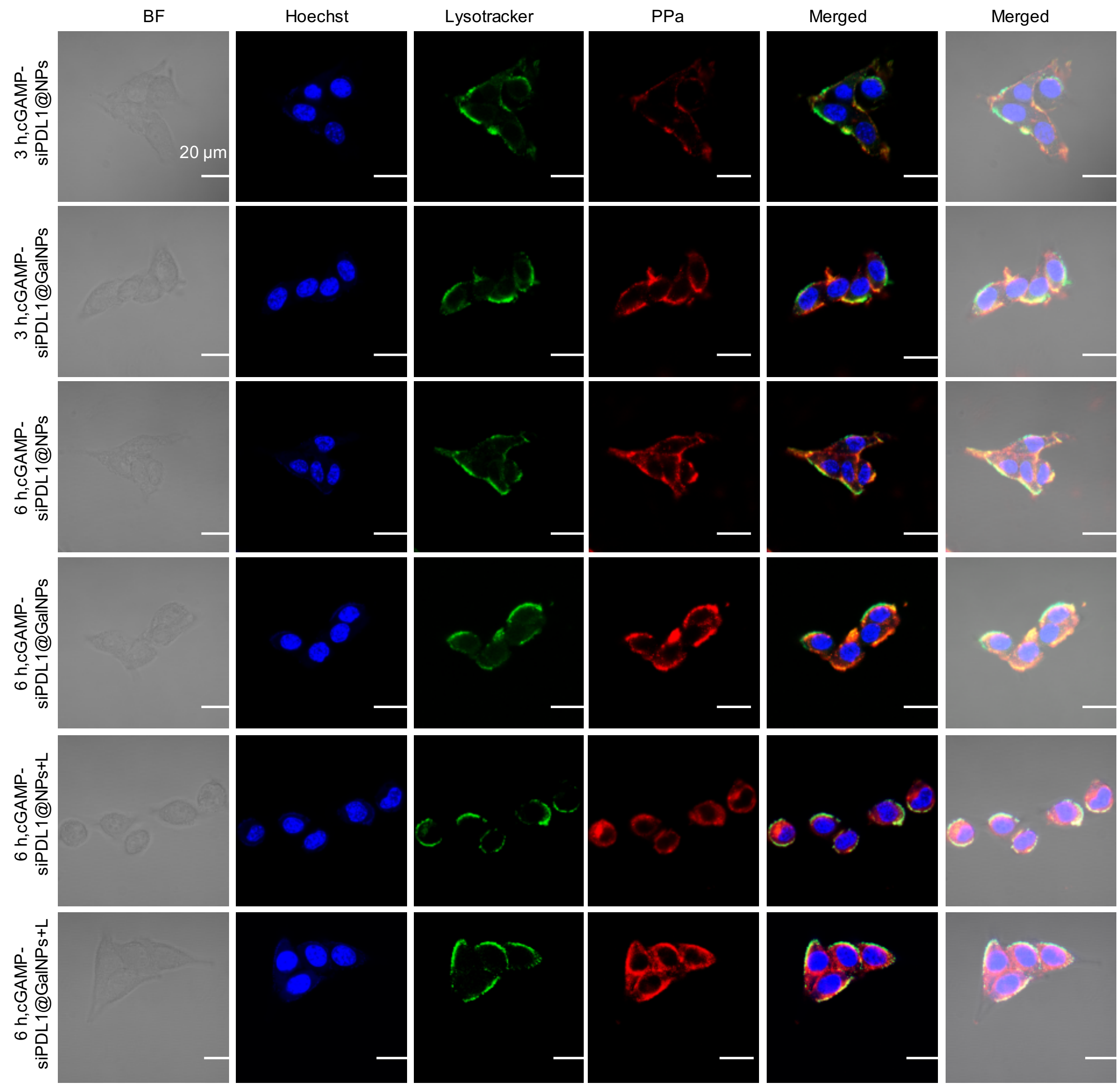

Fig. S16. CLSM images of B16F10 cancer cells after exposing to cGAMP-siPDL1@NPs and cGAMP-siPDL1@GalNPs for 3 and 6 h with/without laser irradiation (660 nm, 5 min, 100 mW/cm^2). Nuclei: blue; lysosomes: green; nanocarriers: red. Scale bar: 20 μm.

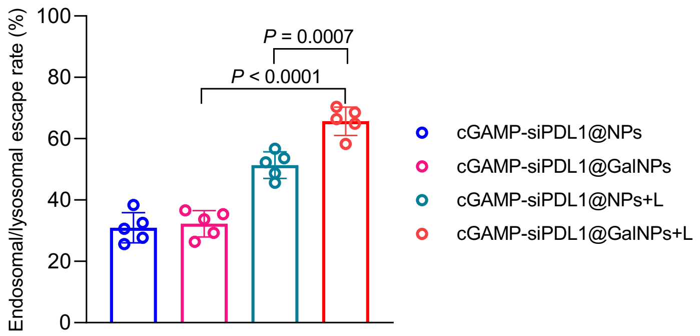

Fig. S17. The endosomal/lysosomal escape rate of nanocarriers from cancer cells was performed by quantifying the CLSM images with the Zeiss LSM880 fluorescence microscope. The data are represented as means ± s.d. (n = 5 section images). Statistical analysis was performed using one-way ANOVA with Tukey's test.

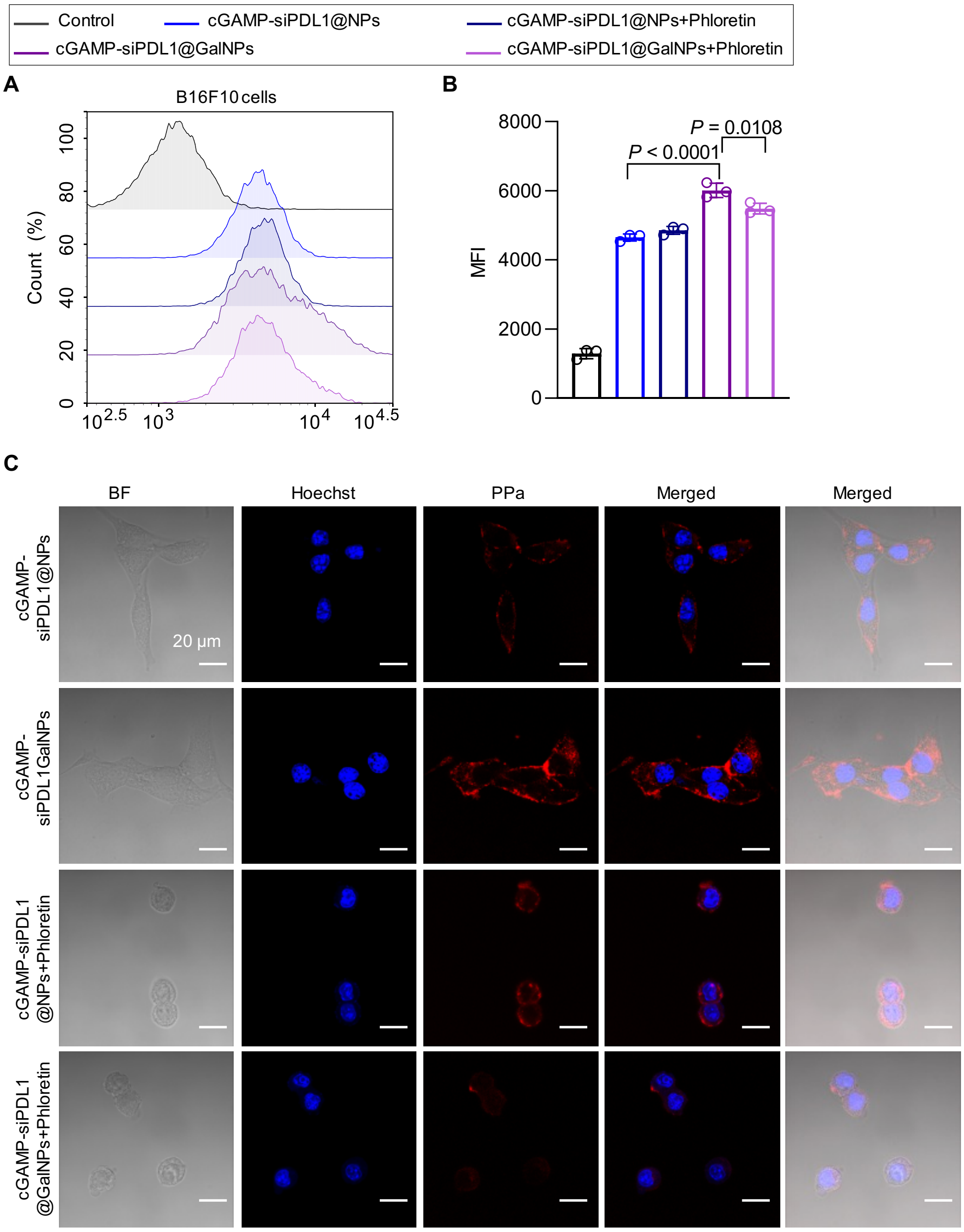

Fig. S18. The cellular uptake of nanocarriers with/without Phloretin inhibition. **A, B.** Cellular uptake of nanocarriers at 6 h after exposing to B16F10 cancer cells measured by flow cytometer (**A**) and the mean fluorescent intensity (MFI) (**B**). **C.** CLSM images of B16F10 cells at 6 h after treating with cGAMP-siPDL1@NPs and cGAMP-siPDL1@GalNPs with/without blocked by phloretin (0.2 mM) 30 min earlier. Color in images: red (PPa in cGAMP-siPDL1@NPs or cGAMP-siPDL1@GalNPs), blue (Hoechst, nuclei). Scale bar, 20 μm. Data were expressed as mean ± s.d. (n = 3). Statistical significance was determined by one-way ANOVA with Tukey's test.

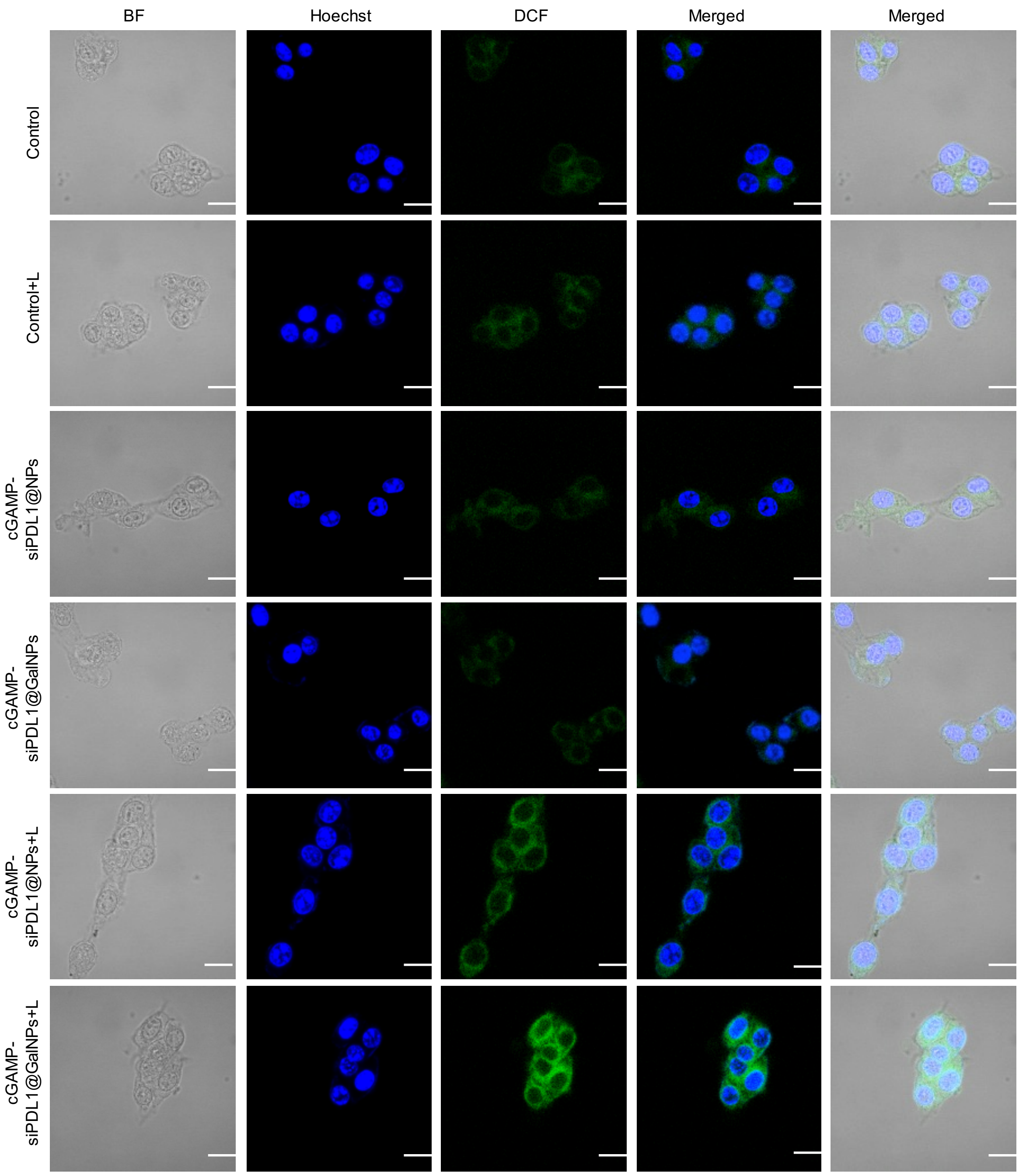

Fig. S19. CLSM images of ROS produced by cGAMP-siPDL1@NPs and cGAMP-siPDL1@GalNPs in B16F10 cells with/without laser irradiation (660 nm, 100 mW/cm^2, 5 min). Nuclei: blue; ROS: green. Scale bar, 20 μm.

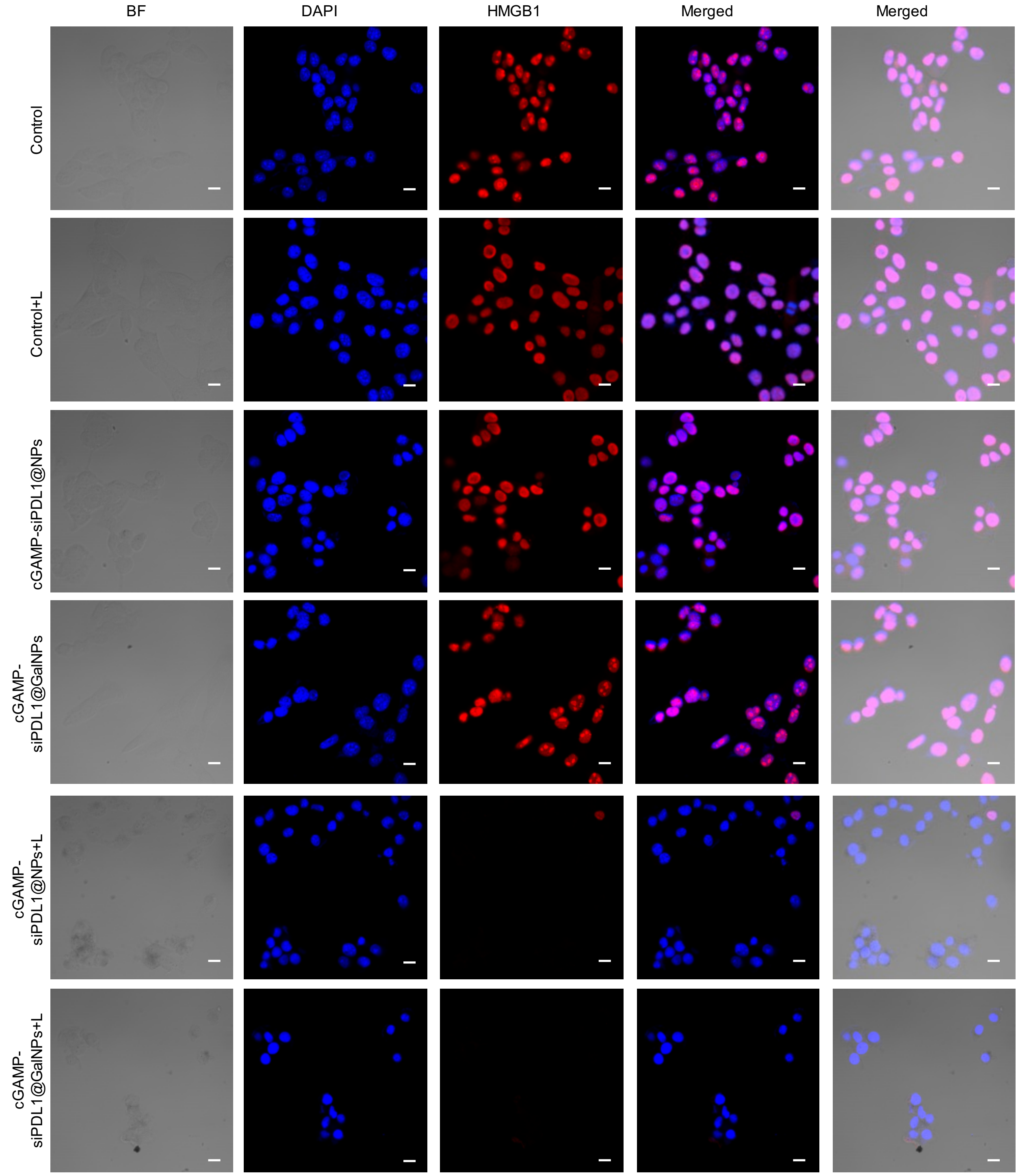

Fig. S20. Immunofluorescence imaging of HMGB1 release treated with cGAMP-siPDL1@NPs and cGAMP-siPDL1@GalNPs in the presence or absence of laser irradiation (660 nm, 100 mW/cm^2, 5 min). Nuclei: blue; HMGB1: red. Scale bar, 20 μm.

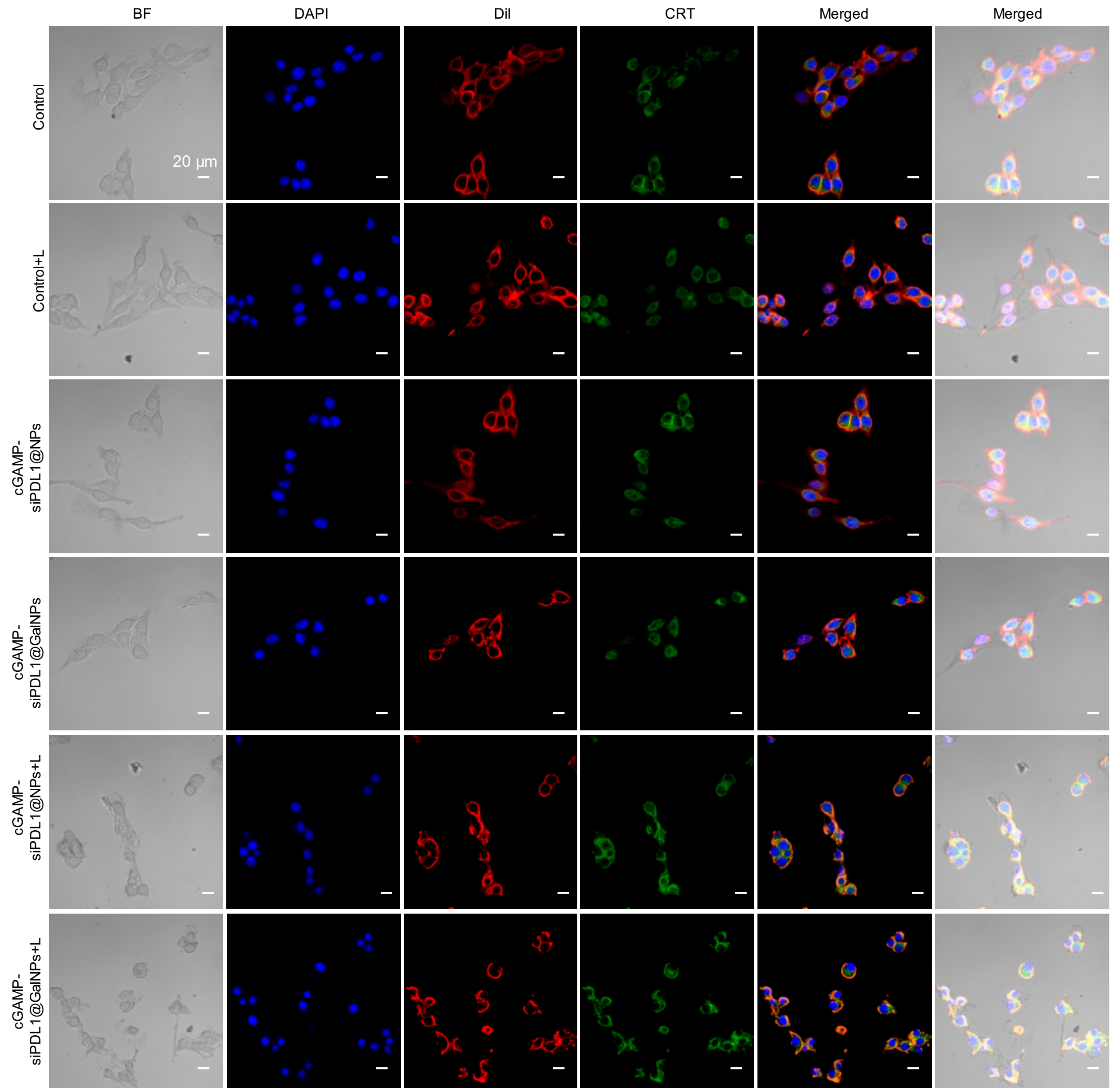

Fig. S21. Immunofluorescence imaging of CRT expression on the cell surface of B16F10 cells treated with cGAMP-siPDL1@NPs and cGAMP-siPDL1@GalNPs in the presence or absence of laser irradiation (660 nm, 100 mW/cm^2, 5 min). Nuclei: blue; CRT: green; cell membranes: red. Overlap of CRT and cell membrane: orange. Scale bar, 20 μm.

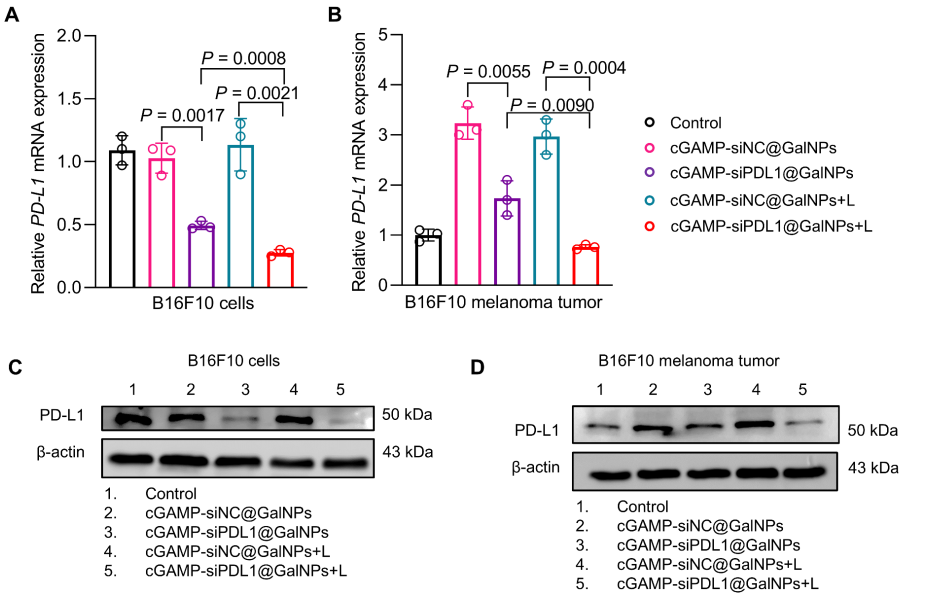

Fig. S22. A, B. qPCR analysis of *PD-L1* mRNA levels in B16F10 cells (**A**) and B16F10 tumors (**B**) after exposure to cGAMP-siNC@GalNPs and cGAMP-siPDL1@GalNPs for 6 h with/without laser irradiation (660 nm, 100 mW/cm^2, 5 min). The data were analyzed by one-way ANOVA with Tukey's test (n = 3). **C, D**. WB analysis of PD-L1 expression in B16F10 cells (**C**) and B16F10 tumors (**D**) when treated with cGAMP-siNC@GalNPs and cGAMP-siPDL1@GalNPs for 6 h with/without laser irradiation (660 nm, 100 mW/cm^2, 5 min).

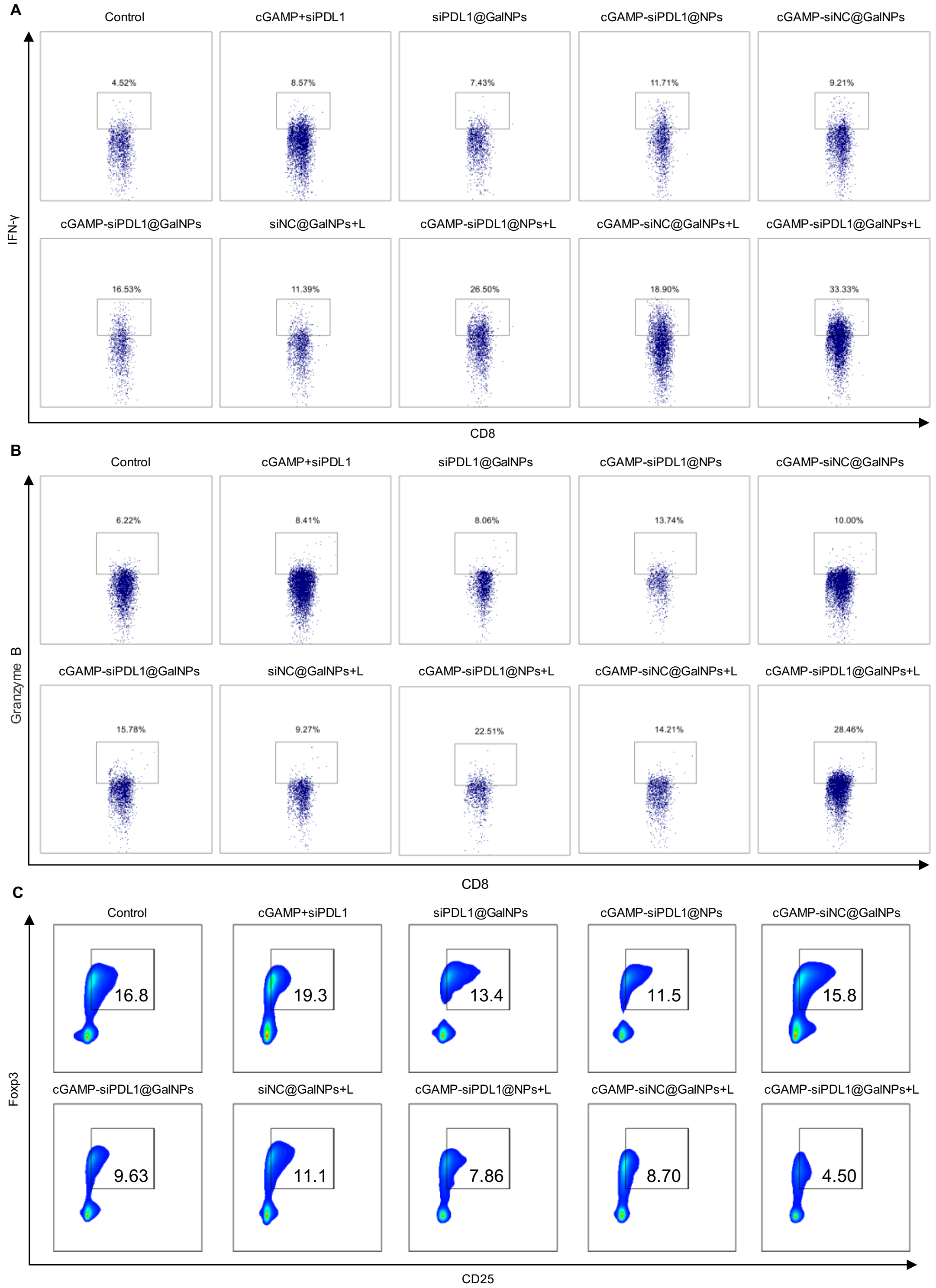

Fig. S23. Representative FCM dot plots of tumor-infiltrating IFN-γ^+CD8$^+$ T cells (**A**), Granzyme B$^+$CD8$^+$ T cells (**B**) and Tregs (CD4$^+$CD25$^+$Foxp3$^+$) (**C**) analyzed at 48 h after drug administration and followed with/without laser irradiation of tumors.

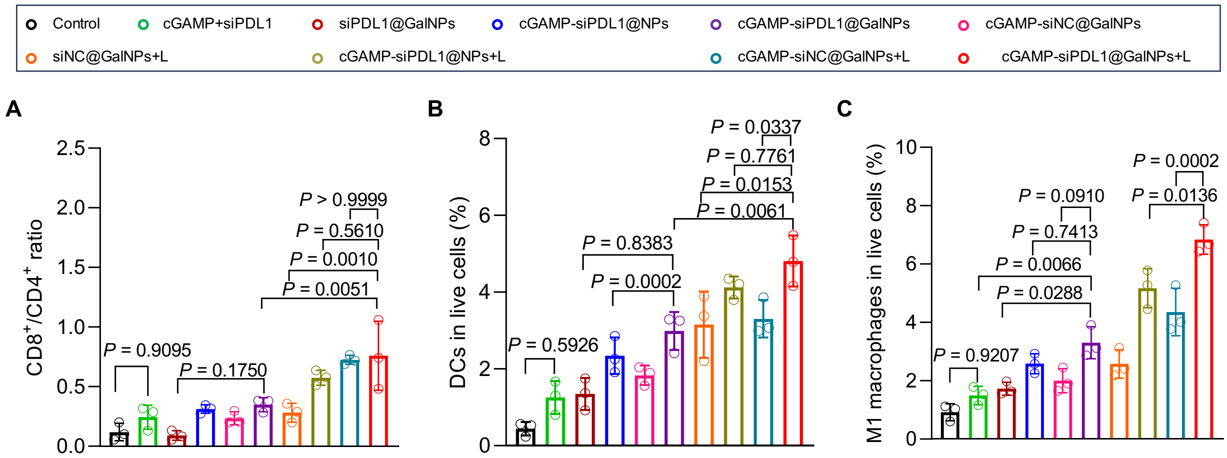

Fig. S24. A. The $CD8^+$ to $CD4^+$ ratio in tumors. **B, C.** Percentages of DCs (**B**) and M1 macrophages (**C**) infiltrating in B16F10 melanoma TME among all live cells. Data are shown as means ± s.d. Statistical analysis was performed using one-way ANOVA with Tukey's test (n = 3 mice).

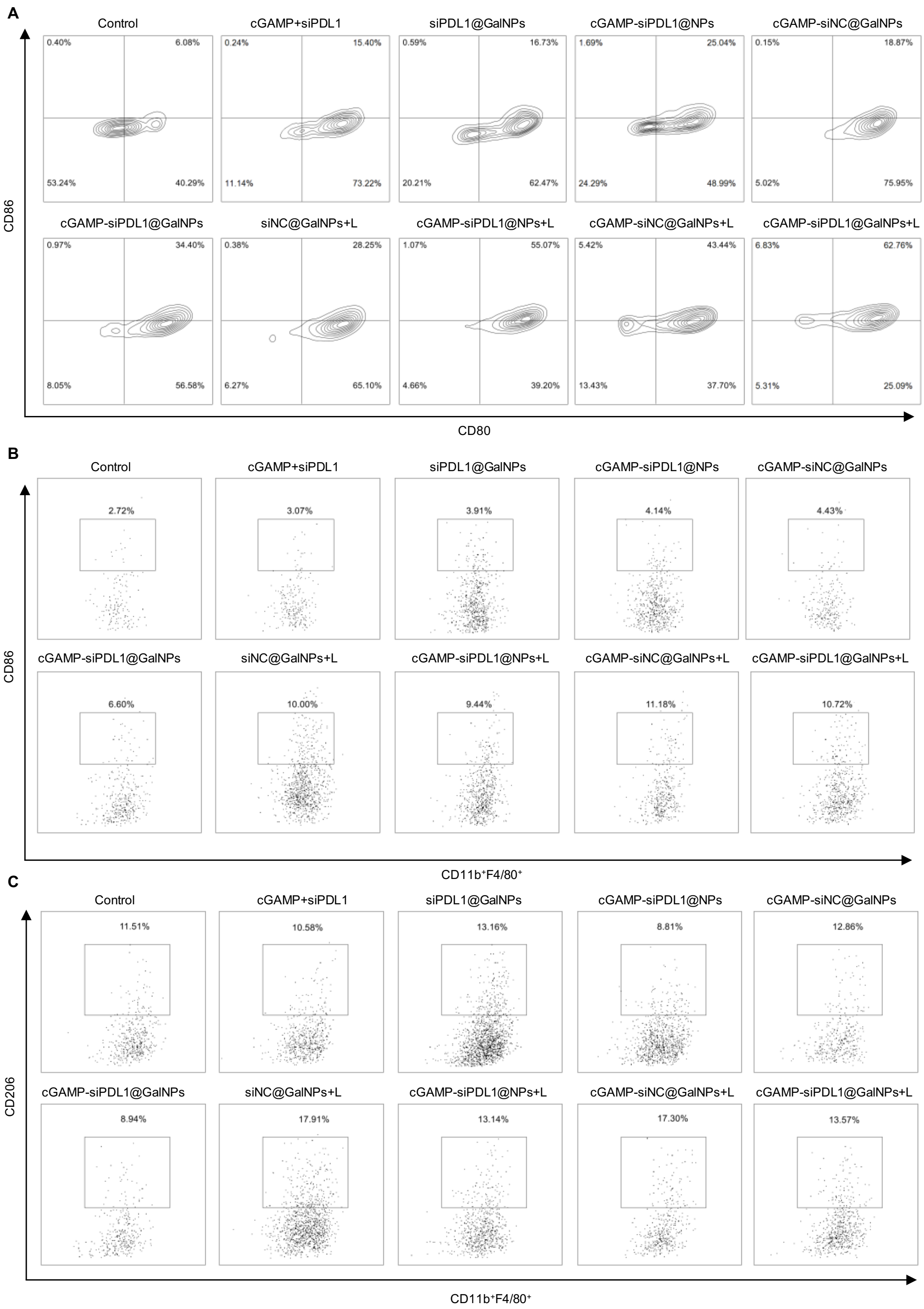

Fig. S25. Representative FCM dot plots of DC maturation ($CD86^{+}CD80^{+}CD11c^{+}$) (**A**), M1 macrophages ($CD86^{+}CD11b^{+}F4/80^{+}$) (**B**) and M2 macrophages ($CD206^{+}CD11b^{+}F4/80^{+}$) (**C**) analyzed at 48 h after drug administration and followed with/without laser irradiation of tumors.

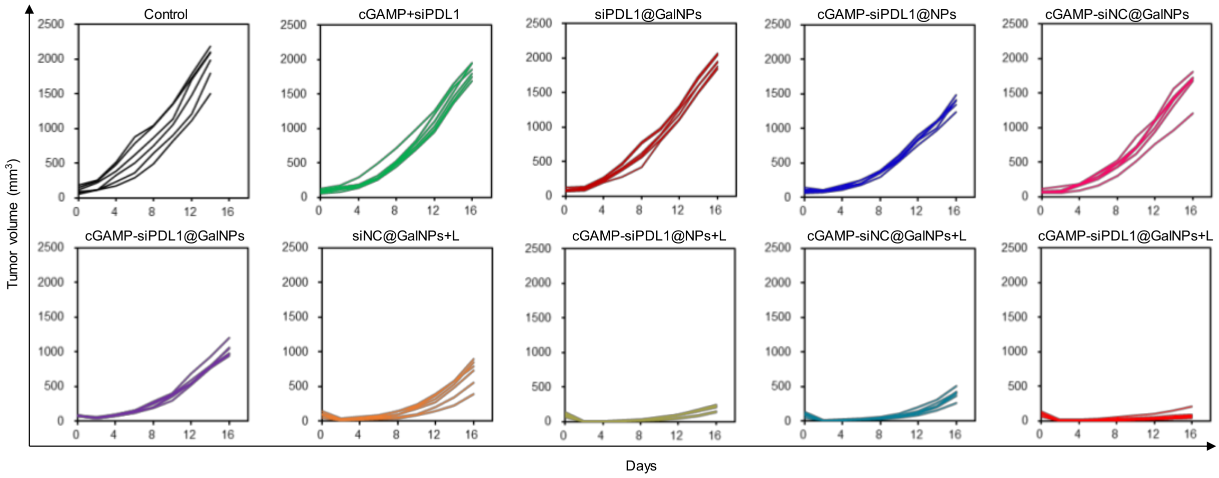

Fig. S26. Individual tumor growth curves of the primary B16F10 melanoma tumors.

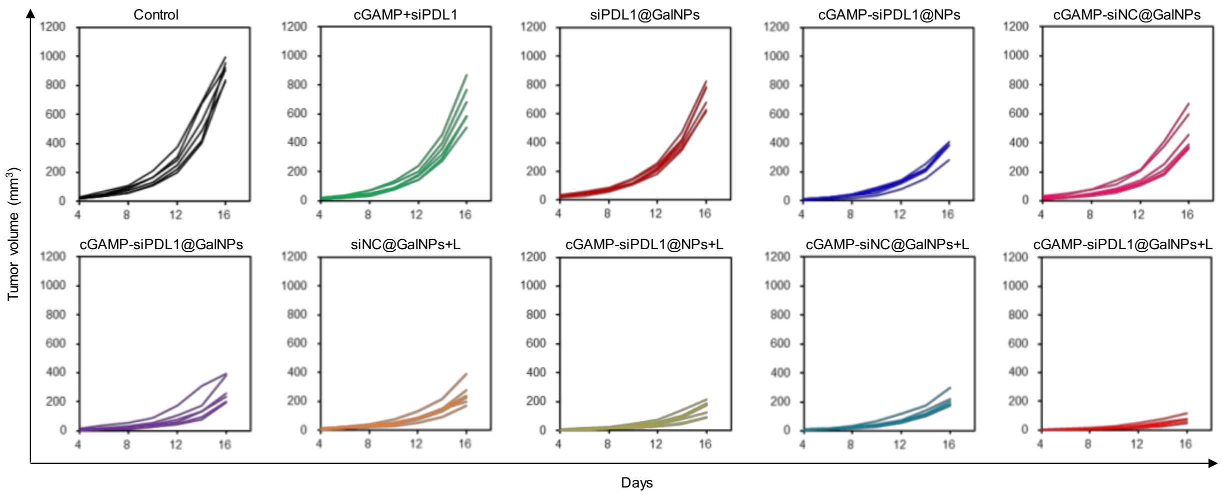

Fig. S27. Individual tumor growth curves of the distant B16F10 melanoma tumors.

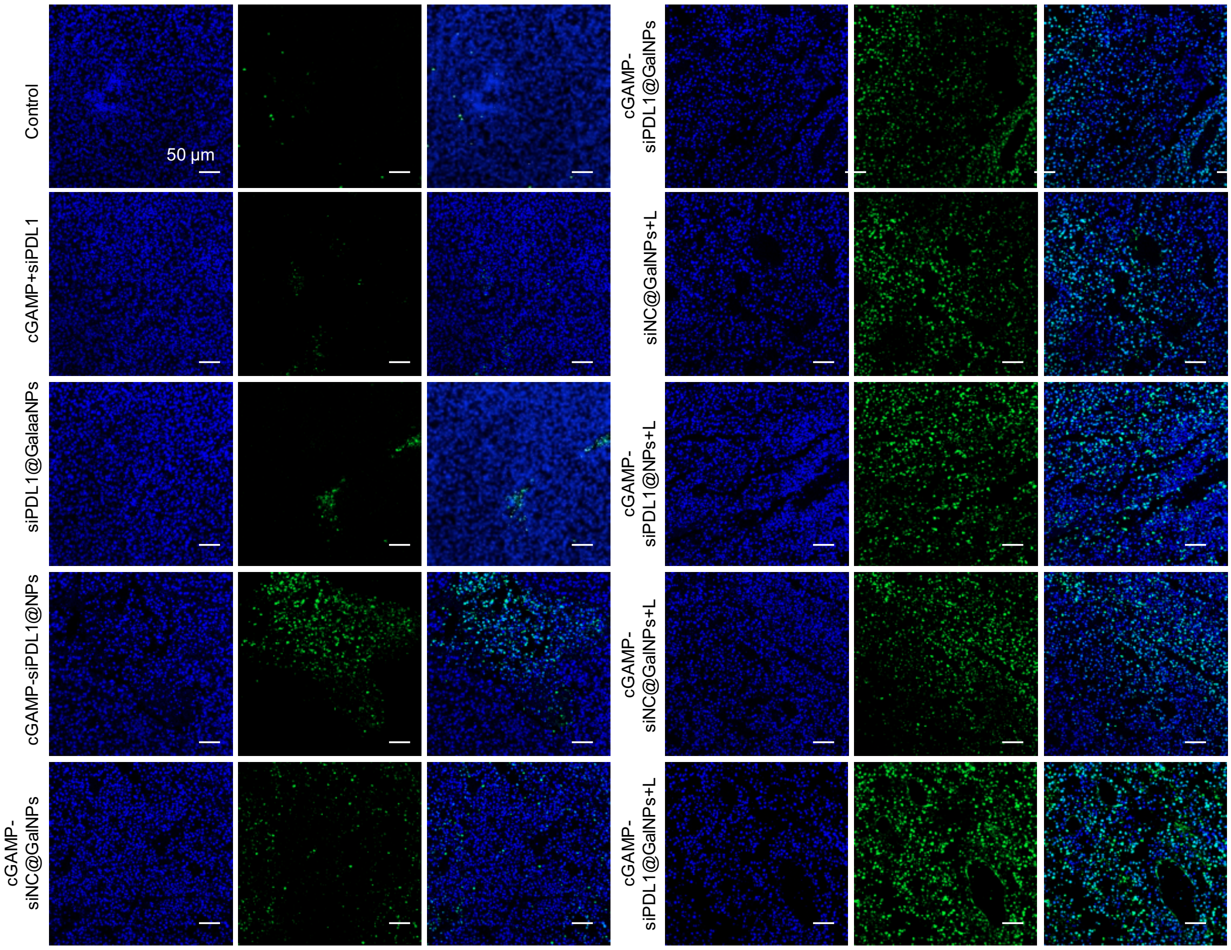

Fig. S28. TUNEL staining of B16F10 tumor on day 2 of the antitumor study.

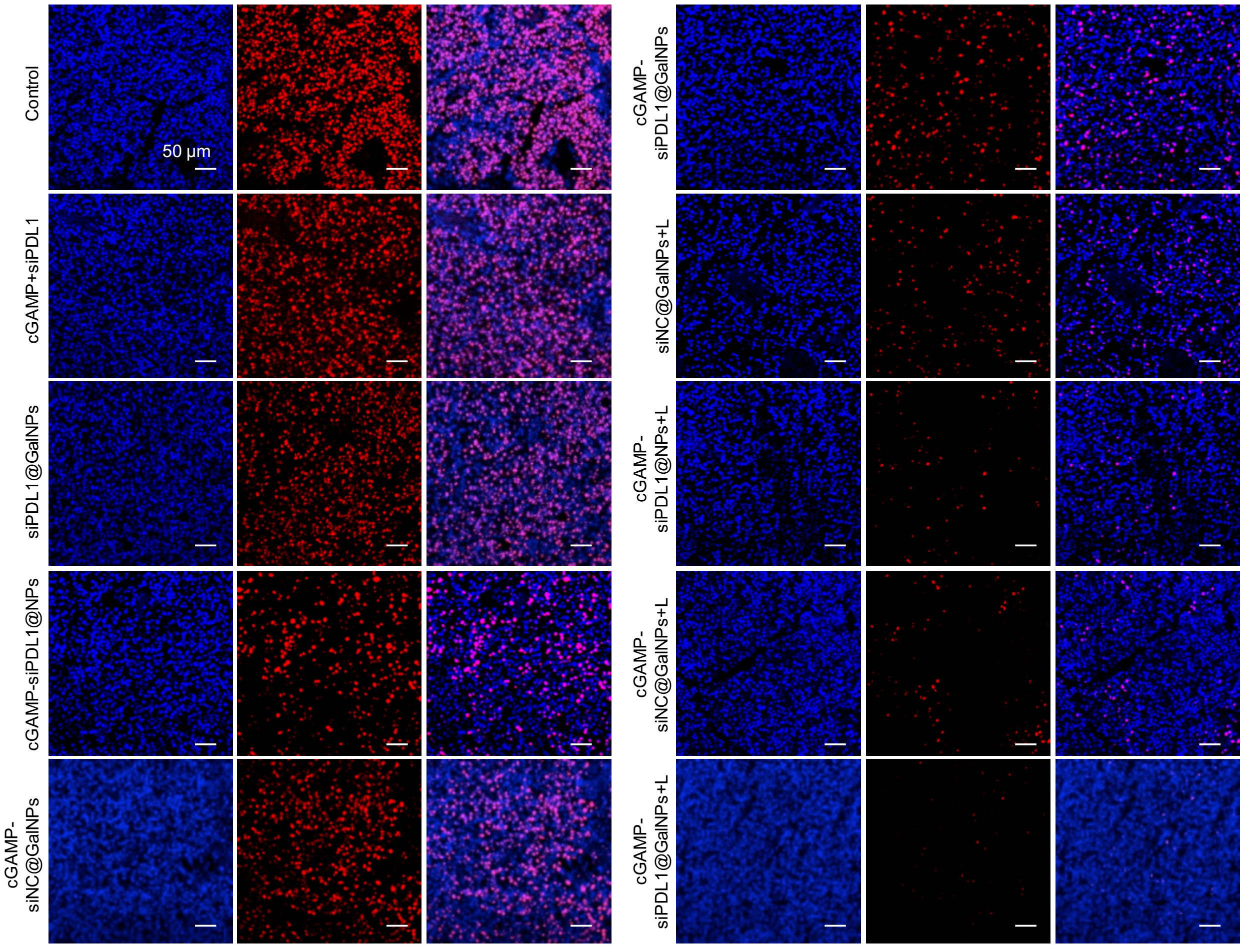

Fig. S29. Ki67 staining of B16F10 tumor on day 2 of the antitumor study.

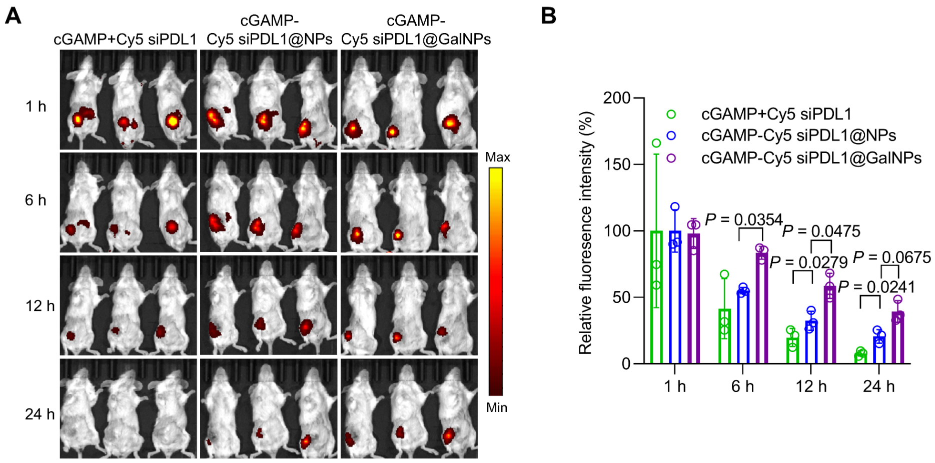

Fig. S30. IVIS images (A) and quantitative analysis (B) show cGAMP-Cy5 siPDL1@NPs and cGAMP-Cy5 siPDL1@GalNPs in orthotopic 4T1 tumors after drug administration. Data are shown as means ± s.d. Statistical analysis was performed using one-way ANOVA with Tukey's test (n = 3 mice).

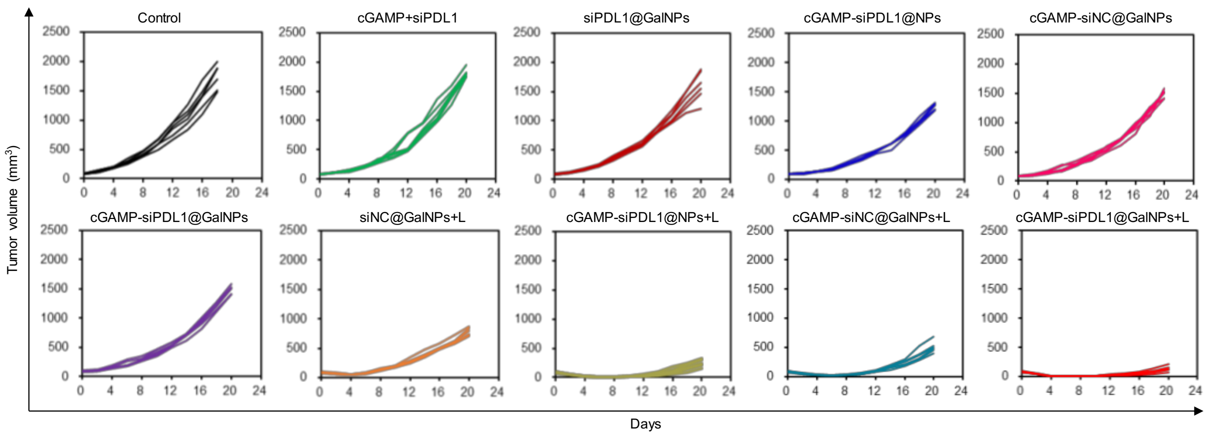

Fig. S31. Individual tumor growth curves of the orthotopic 4T1 tumors.

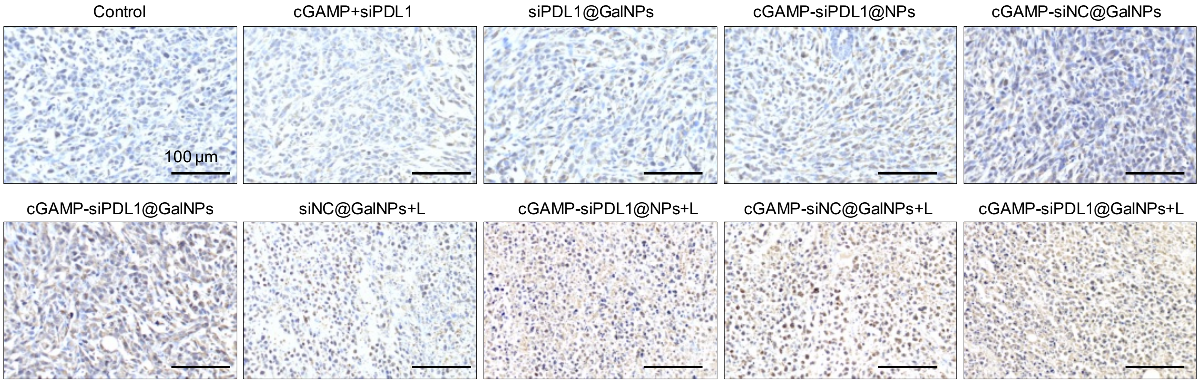

Fig. S32. IHC staining of cleaved caspase 3 in the orthotopic breast tumors obtained on day 2.

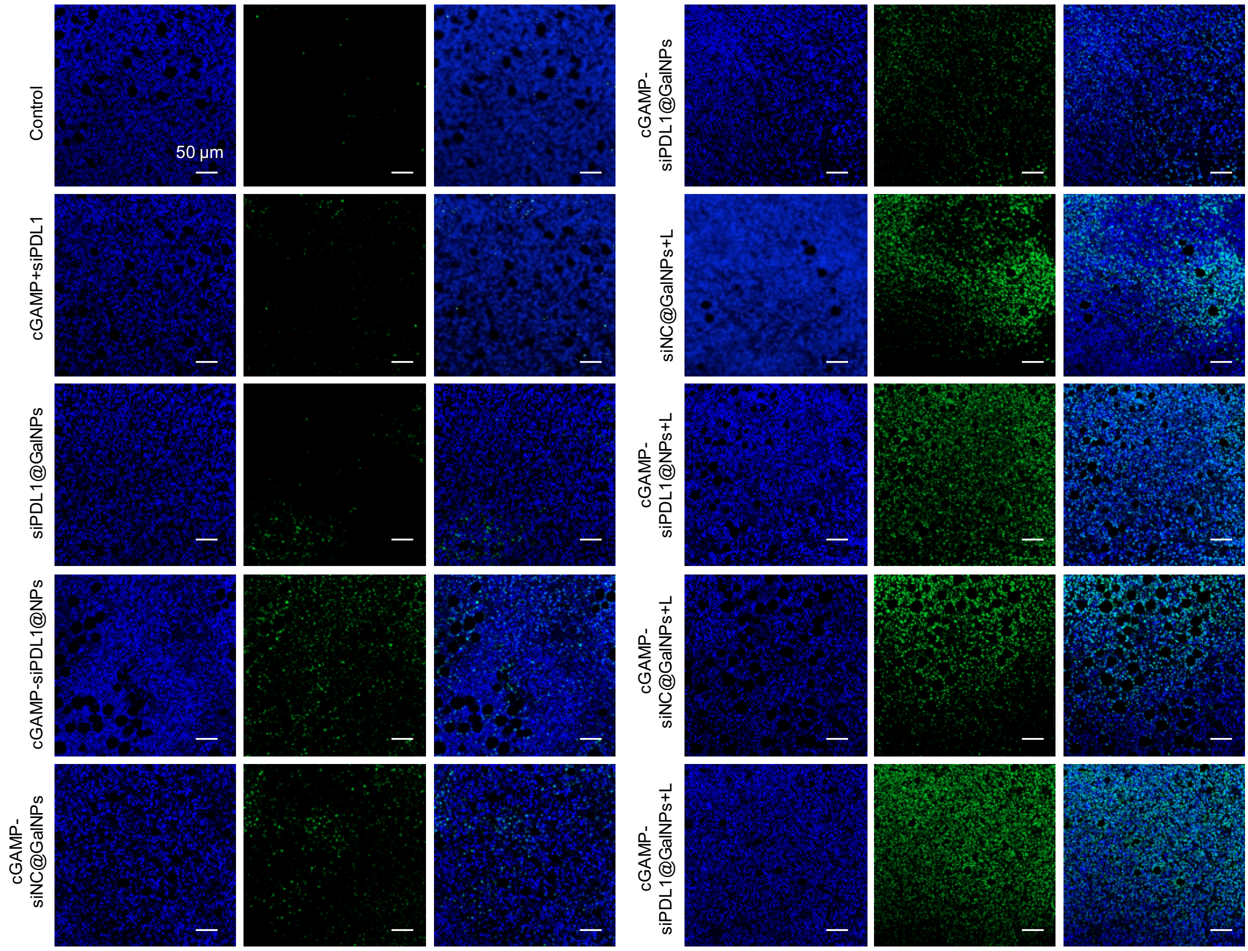

Fig. S33. TUNEL staining of the orthotopic breast tumors on day 2 of the antitumor study.

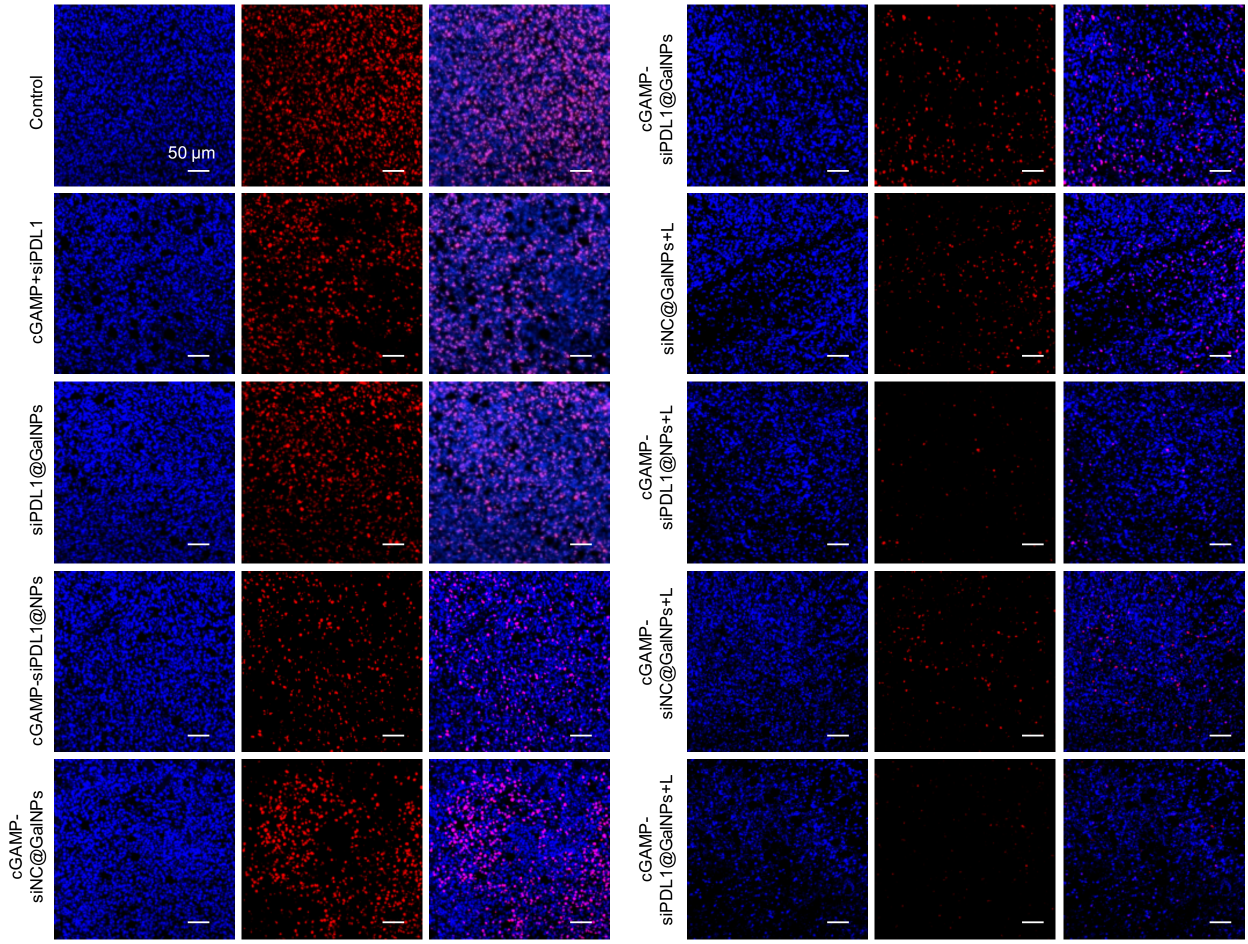

Fig. S34. Ki67 staining of the orthotopic breast tumors on day 2 of the antitumor study.

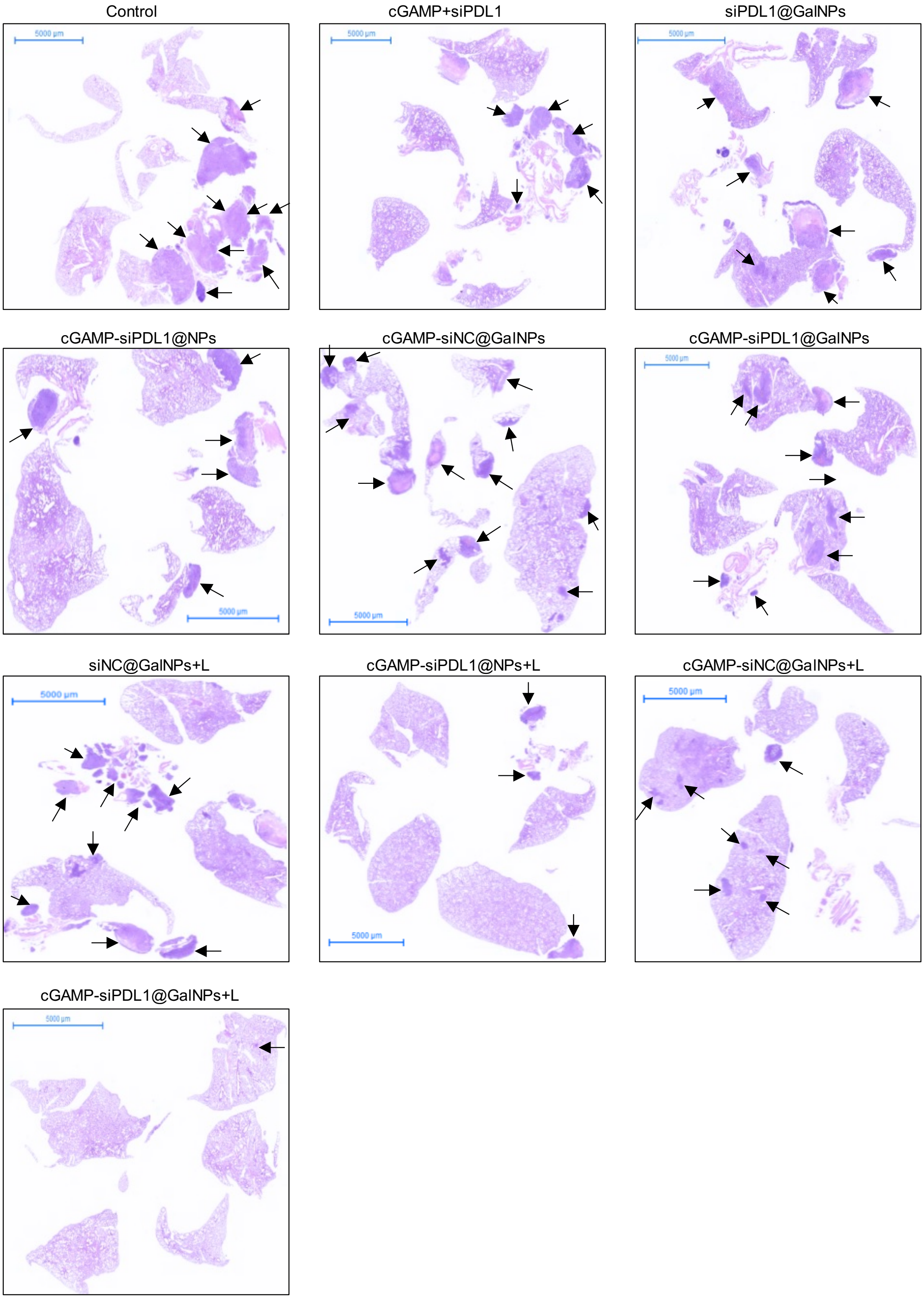

Fig. S35. H&E staining shows spontaneous lung metastases from the orthotopic 4T1-Luc tumors. Black arrows point to lung metastatic tumors.

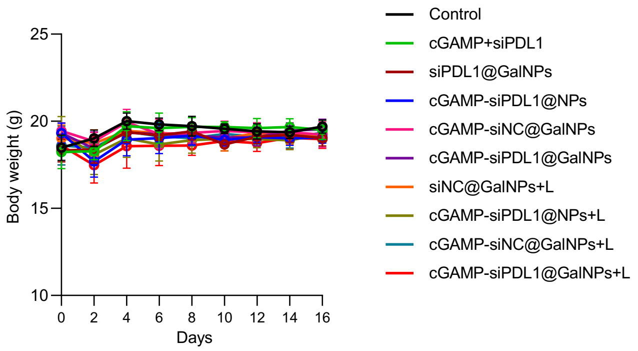

Fig. S36. Body weight of B16F10 tumor-bearing mice during the treatment period. Data were expressed as mean ± SD. (n = 6 mice).

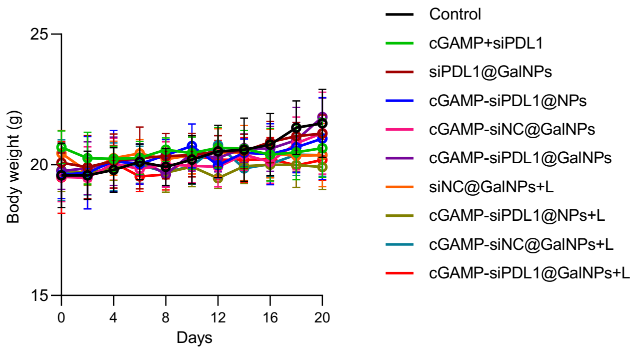

Fig. S37. Body weight of the 4T1 breast tumor-bearing mice during the treatment period. Data were expressed as mean ± SD. (n = 6 mice)

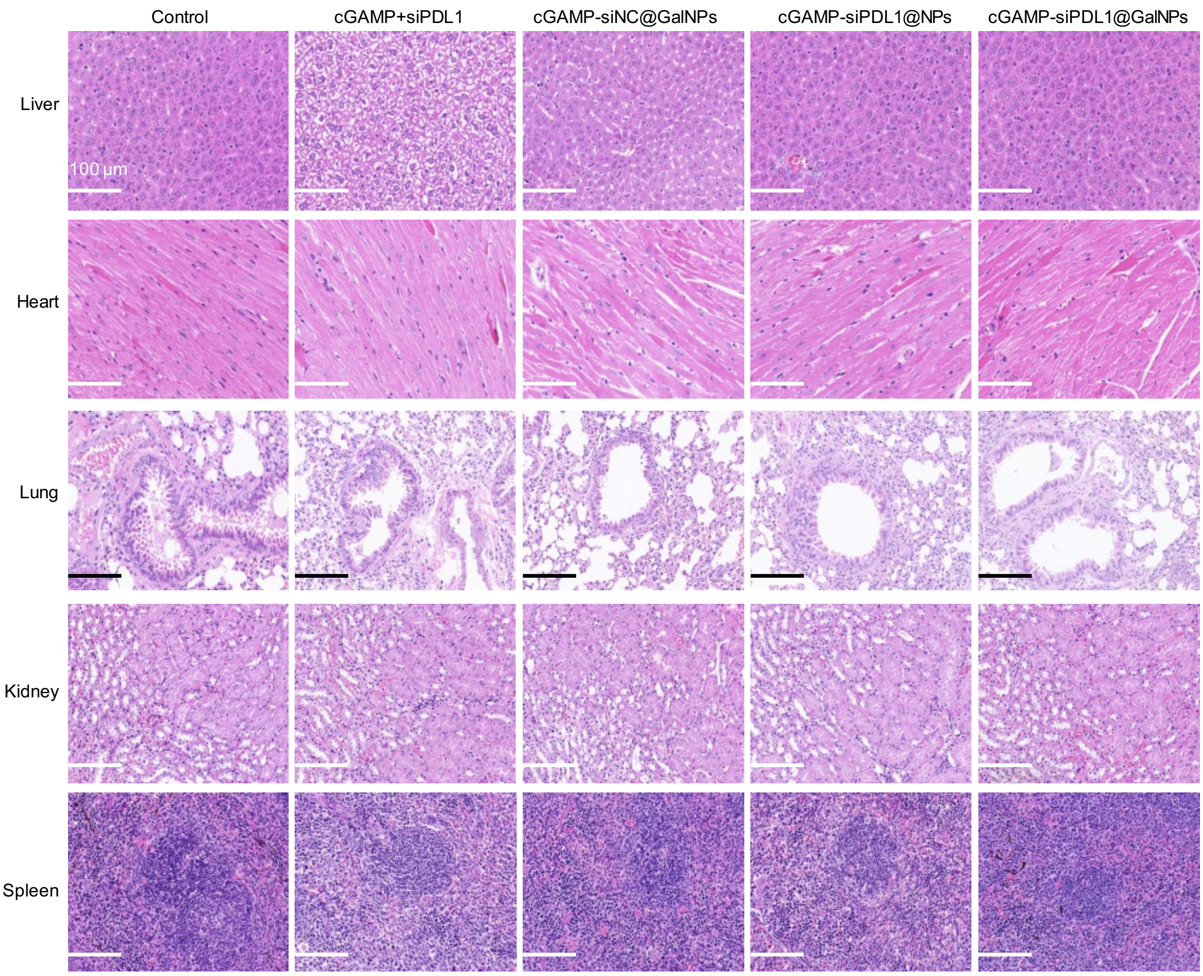

Fig. S38. Histological analysis of major organs. H&E staining of heart, liver, lung and kidney from B16F10 melanoma tumor-bearing mice at day 3 after administration of cGAMP+siPDL1, cGAMP-siNC@NPs, cGAMP-siPDL1@NPs and cGAMP-siPDL1@GalNPs (Dose: cGAMP = 15 µg, siPDL1 = 15 µg, siNC = 15 µg). Scale bar, 100 µm.

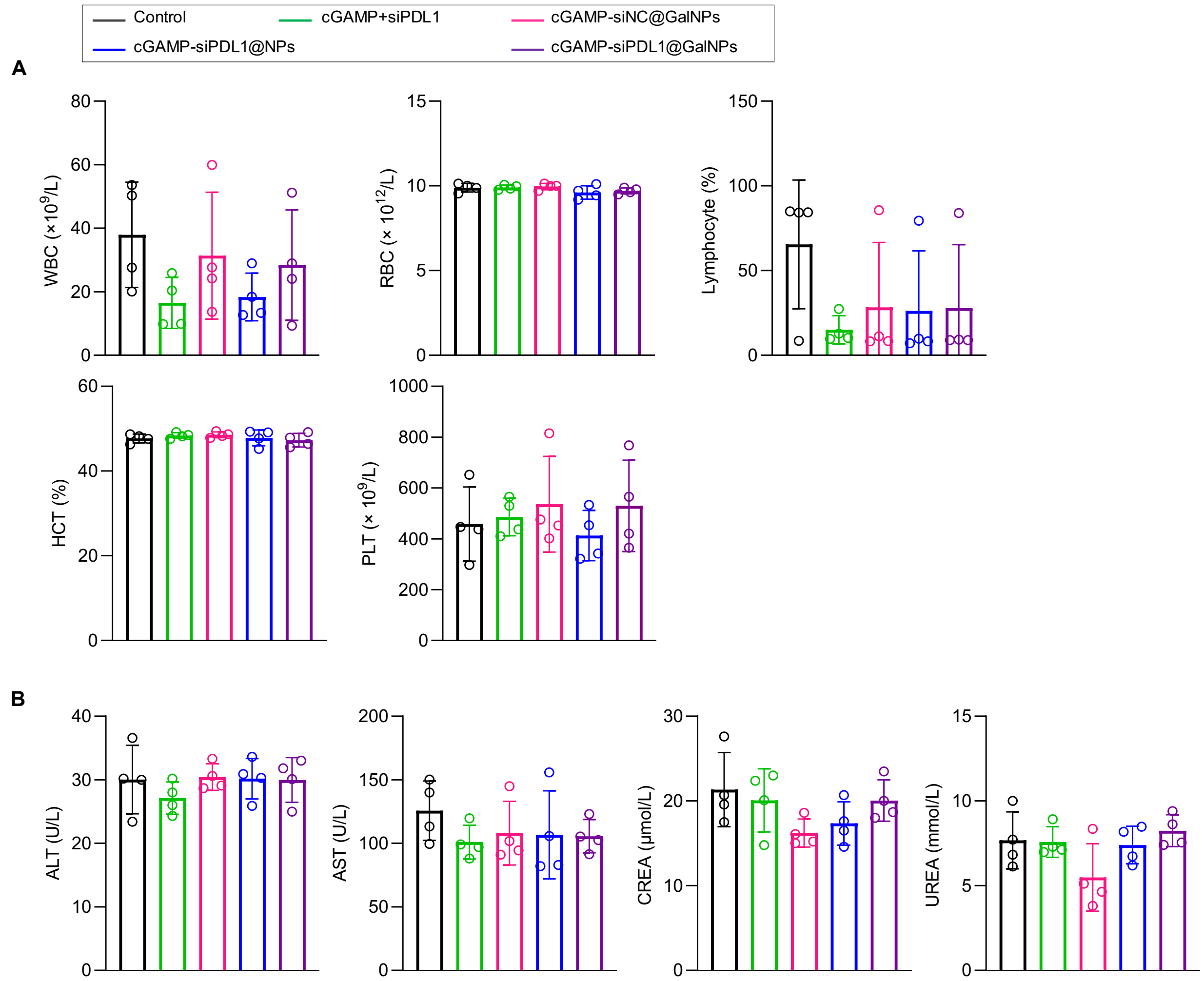

Fig. S39. Blood test results. **A**, **B.** Blood routine examinations (**A**) and serum biochemistry data (**B**) of mice when reached the endpoint of tumor size. Data were expressed as mean ± s.d. (n = 4).

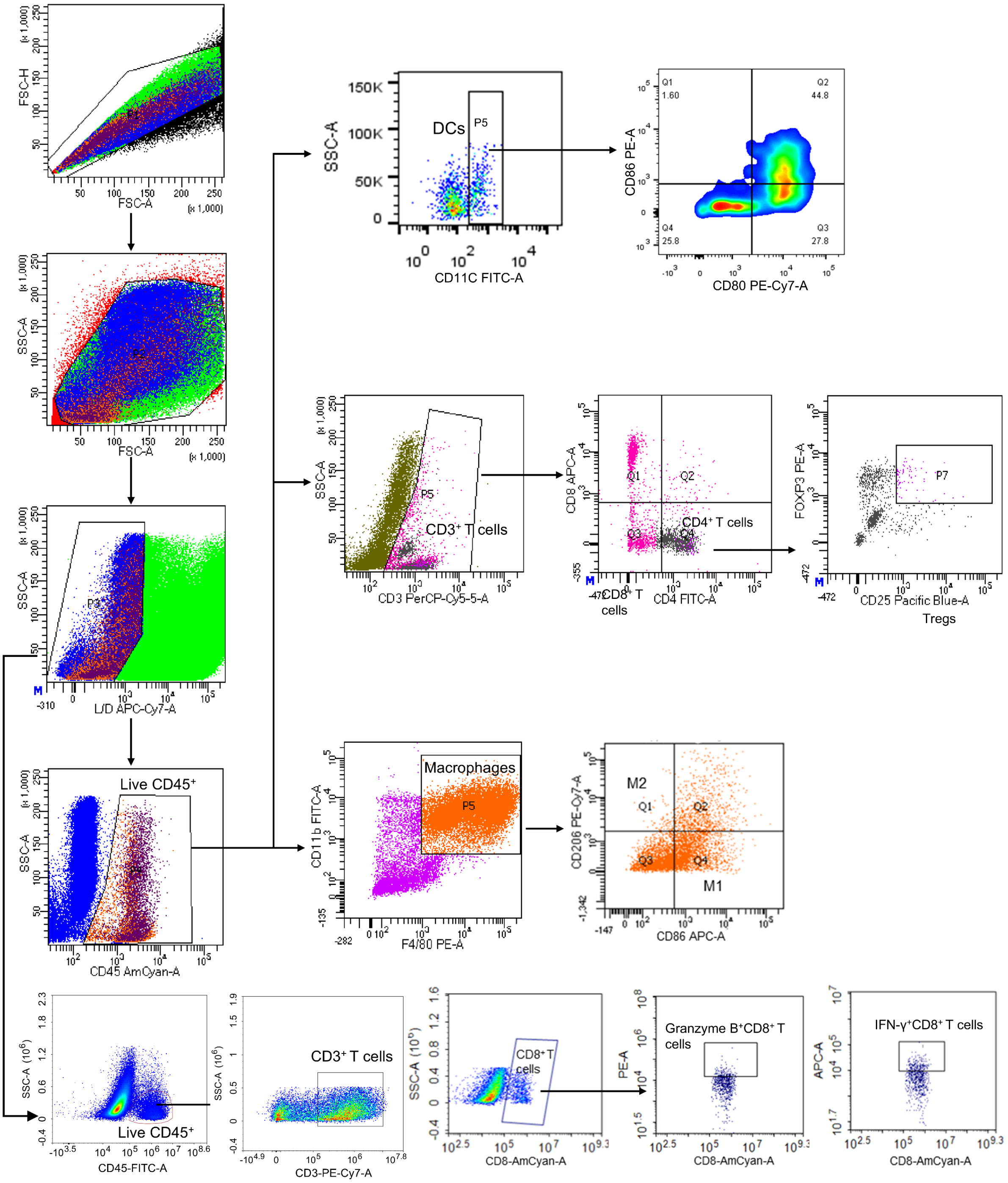

Fig. S40. Gating strategies for analysis immune cells by FCM. The above gating strategy was applied for analysis data in **Fig. 6** and **Fig. S23-25**.

2.2 Supplementary tables

Table S1. Antibodies used in this study. PerCP, peridinin chlorophyll protein complex; PE, phycoerythrin; APC, allophycocyanin; FITC, fluorescein isothiocyanate; Cy, Cyanine; BV, Brilliant Violent.

**Species**	**Fluorophore**	**Antibody**	**Vendor**	**Catalog number**	**Recommended concentration**
**Flow cytometry**					
anti-mouse	BV510	CD45	BioLegend	103137	5 μl/10^6 cells
anti-mouse	PerCP-Cy5.5	CD3e	Invitrogen	45-0031-82	5 μl/10^6 cells
anti-mouse	FITC	CD4	Invitrogen	11-0042-82	0.4 μl/10^6 cells
anti-mouse	APC	CD8a	Invitrogen	17-0081-82	0.63 μl/10^6 cells
anti-mouse	BV421	CD25	BioLegend	101923	0.63 μl/10^6 cells
anti-mouse	PE	Foxp3	Invitrogen	12-5773-82	5 μl/10^6 cells
anti-mouse	FITC	CD45	Invitrogen	11-0451-82	1 μl/10^6 cells
anti-mouse	PE-Cy7	CD3e	Biolegend	100220	1.25 μl/10^6 cells
anti-mouse	BV510	CD8a	BioLegend	10072	2.5 μl/10^6 cells
anti-mouse	PE	Granzyme B	Invitrogen	12-8898-82	0.625μl/10^6 cells
anti-mouse	APC	IFN-γ	BioLegend	505810	5 μl/10^6 cells
anti-mouse	APC	CD11c	BioLegend	117310	1.25 μl/10^6 cells
anti-mouse	PE/Cy7	CD80	BioLegend	104734	2.5μl /10^6 cells
anti-mouse	PE	CD86	BioLegend	105008	5 μl/10^6 cells
anti-mouse	FITC	CD11b	BioLegend	101205	0.5 μl/10^6 cells
anti-mouse	PE	F4/80	BioLegend	123110	5 μl/10^6 cells
anti-mouse	PE/Cy7	CD206	BioLegend	141720	1.25 μl/10^6 cells
anti-mouse	APC	CD86	BioLegend	159216	0.625μl/10^6 cells
**Western blot**					
anti-rabbit		IRF3	Cell Signaling Technology (CST)	4302S	1:1000
anti-rabbit		TBK1	CST	3504T	1:1000
anti-rabbit		P^{Ser396}-IRF3	CST	4947s	1:1000
anti-rabbit		P^{Ser172}-TBK1	CST	54835s	1:1000
anti-rabbit		PD-L1	Abcam	ab213480	1:1000
anti-rabbit		HMGB1	HUABIO	ET1601-2	1:5000
anti-rabbit		β-actin	Abcam	ab8227	1:2000
		Secondary antibodies	HUABIO	HA1001	1:50000
**Immunofluorescence**					
anti-rabbit		HMGB1	HUABIO	ET1601-2	1:100
anti-rabbit		CRT	Abcam	Ab92516	1:500
	iFluor™ 488	Secondary antibodies	HUABIO	HA1121	1:1000

REFERENCES AND NOTES

1. K. M. Mahoney, P. D. Rennert, G. J. Freeman, Combination cancer immunotherapy and new immunomodulatory targets. *Nat. Rev. Drug Discov.* **14**, 561–584 (2015).

2. P. Sharma, J. P. Allison, The future of immune checkpoint therapy. *Science* **348**, 56–61 (2015).

3. M. Yarmarkovich, A. Farrel, A. Sison, M. di Marco, P. Raman, J. L. Parris, D. Monos, H. Lee, S. Stevanovic, J. M. Maris, Immunogenicity and immune silence in human cancer. *Front. Immunol.* **11**, 69 (2020).

4. Q. Chen, L. Sun, Z. J. Chen, Regulation and function of the cGAS-STING pathway of cytosolic DNA sensing. *Nat. Immunol.* **17**, 1142–1149 (2016).

5. G. N. Barber, STING: Infection, inflammation and cancer. *Nat. Rev. Immunol.* **15**, 760–770 (2015).

6. T. F. Gajewski, E. F. Higgs, Immunotherapy with a sting. *Science* **369**, 921–922 (2020).

7. E. N. Chin, C. Yu, V. F. Vartabedian, Y. Jia, M. Kumar, A. M. Gamo, W. Vernier, S. H. Ali, M. Kissai, D. C. Lazar, N. Nguyen, L. E. Pereira, B. Benish, A. K. Woods, S. B. Joseph, A. Chu, K. A. Johnson, P. N. Sander, F. Martínez-Peña, E. N. Hampton, T. S. Young, D. W. Wolan, A. K. Chatterjee, P. G. Schultz, H. M. Petrassi, J. R. Teijaro, L. L. Lairson, Antitumor activity of a systemic STING-activating non-nucleotide cGAMP mimetic. *Science* **369**, 993–999 (2020).

8. S. Yum, M. Li, Z. J. Chen, Old dogs, new trick: Classic cancer therapies activate cGAS. *Cell Res.* **30**, 639–648 (2020).

9. A. Decout, J. D. Katz, S. Venkatraman, A. Ablasser, The cGAS-STING pathway as a therapeutic target in inflammatory diseases. *Nat. Rev. Immunol.* **21**, 548–569 (2021).

10. S. Skopelja-Gardner, J. An, K. B. Elkon, Role of the cGAS-STING pathway in systemic and organ-specific diseases. *Nat. Rev. Nephrol.* **18**, 558–572 (2022).

11. K. P. Hopfner, V. Hornung, Molecular mechanisms and cellular functions of cGAS-STING signalling. *Nat. Rev. Mol. Cell Biol.* **21**, 501–521 (2020).

12. A. Ablasser, Z. J. Chen, cGAS in action: Expanding roles in immunity and inflammation. *Science* **363**, eaat8657 (2019).

13. M. McLaughlin, E. C. Patin, M. Pedersen, A. Wilkins, M. T. Dillon, A. A. Melcher, K. J. Harrington, Inflammatory microenvironment remodelling by tumour cells after radiotherapy. *Nat. Rev. Cancer* **20**, 203–217 (2020).

14. S. Lee, K. Margolin, Cytokines in cancer immunotherapy. *Cancers* **3**, 3856–3893 (2011).

15. W. Li, L. Lu, J. Lu, X. Wang, C. Yang, J. Jin, L. Wu, X. Hong, F. Li, D. Cao, Y. Yang, M. Wu, B. Su, J. Cheng, X. Yang, W. Di, L. Deng, cGAS-STING-mediated DNA sensing maintains CD8+ T cell stemness and promotes antitumor T cell therapy. *Sci. Transl. Med.* **12**, eaay9013 (2020).

16. B. Jneid, A. Bochnakian, C. Hoffmann, F. Delisle, E. Djacoto, P. Sirven, J. Denizeau, C. Sedlik, Y. Gerber-Ferder, F. Fiore, R. Akyol, C. Brousse, R. Kramer, I. Walters, S. Carlioz, H. Salmon, B. Malissen, M. Dalod, E. Piaggio, N. Manel, Selective STING stimulation in dendritic cells primes antitumor T cell responses. *Sci. Immunol.* **8**, eabn6612 (2023).

17. Q. Wang, J. S. Bergholz, L. Ding, Z. Lin, S. K. Kabraji, M. E. Hughes, X. He, S. Xie, T. Jiang, W. Wang, J. J. Zoeller, H. J. Kim, T. M. Roberts, P. A. Konstantinopoulos, U. A. Matulonis, D. A. Dillon, E. P. Winer, N. U. Lin, J. J. Zhao, STING agonism reprograms tumor-associated macrophages and overcomes resistance to PARP inhibition in BRCA1-deficient models of breast cancer. *Nat. Commun.* **13**, 3022 (2022).

18. L. Vornholz, S. E. Isay, Z. Kurgyis, D. C. Strobl, P. Loll, M. H. Mosa, M. D. Luecken, M. Sterr, H. Lickert, C. Winter, F. R. Greten, H. F. Farin, F. J. Theis, J. Ruland, Synthetic enforcement of STING signaling in cancer cells appropriates the immune microenvironment for checkpoint inhibitor therapy. *Sci. Adv.* **9**, eadd8564 (2023).

19. X. Jiang, T. Luo, K. Yang, M. J. Lee, J. Liu, L. Tillman, W. Zhen, R. R. Weichselbaum, W. Lin, STING activation disrupts tumor vasculature to overcome the EPR limitation and increase drug deposition. *Sci. Adv.* **10**, eado0082 (2024).

20. S. Chattopadhyay, Y. H. Liu, Z. S. Fang, C. L. Lin, J. C. Lin, B. Y. Yao, C. M. J. Hu, Synthetic immunogenic cell death mediated by intracellular delivery of STING agonist nanoshells enhances anticancer chemo-immunotherapy. *Nano Lett.* **20**, 2246–2256 (2020).

21. T. Su, Y. Zhang, K. Valerie, X. Y. Wang, S. Lin, G. Zhu, STING activation in cancer immunotherapy. *Theranostics* **9**, 7759–7771 (2019).

22. B. S. Pan, S. A. Perera, J. A. Piesvaux, J. P. Presland, G. K. Schroeder, J. N. Cumming, B. W. Trotter, M. D. Altman, A. V. Buevich, B. Cash, S. Cemerski, W. Chang, Y. Chen, P. J. Dandliker, G. Feng, A. Haidle, T. Henderson, J. Jewell, I. Kariv, I. Knemeyer, J. Kopinja, B. M. Lacey, J. Laskey, C. A. Lesburg, R. Liang, B. J. Long, M. Lu, Y. Ma, E. C. Minnihan, G. O'Donnell, R. Otte, L. Price, L. Rakhilina, B. Sauvagnat, S. Sharma, S. Tyagarajan, H. Woo, D. F. Wyss, S. Xu, D. J. Bennett, G. H. Addona, An orally available non-nucleotide STING agonist with antitumor activity. *Science* **369**, eaba6098 (2020).

23. D. Shae, K. W. Becker, P. Christov, D. S. Yun, A. K. R. Lytton-Jean, S. Sevimli, M. Ascano, M. Kelley, D. B. Johnson, J. M. Balko, J. T. Wilson, Endosomolytic polymersomes increase the activity of cyclic dinucleotide STING agonists to enhance cancer immunotherapy. *Nat. Nanotechnol.* **14**, 269–278 (2019).

24. Y. Liu, W. N. Crowe, L. Wang, Y. Lu, W. J. Petty, A. A. Habib, D. Zhao, An inhalable nanoparticulate STING agonist synergizes with radiotherapy to confer long-term control of lung metastases. *Nat. Commun.* **10**, 5108 (2019).

25. T. W. Dubensky, Jr., D. B. Kanne, M. L. Leong, Rationale, progress and development of vaccines utilizing STING-activating cyclic dinucleotide adjuvants. *Ther. Adv. Vaccines* **1**, 131–143 (2013).

26. L. Zhou, B. Hou, D. Wang, F. Sun, R. Song, Q. Shao, H. Wang, H. Yu, Y. Li, Engineering polymeric prodrug nanoplatform for vaccination immunotherapy of cancer. *Nano Lett.* **20**, 4393–4402 (2020).

27. E. E. Parkes, S. M. Walker, L. E. Taggart, N. McCabe, L. A. Knight, R. Wilkinson, K. D. McCloskey, N. E. Buckley, K. I. Savage, M. Salto-Tellez, S. McQuaid, M. T. Harte, P. B. Mullan, D. P. Harkin, R. D. Kennedy, Activation of STING-dependent innate immune signaling by S-phase-specific DNA damage in breast cancer. *J. Natl. Cancer Inst.* **109**, djw199 (2016).

28. L. M. Snell, T. L. McGaha, D. G. Brooks, Type I interferon in chronic virus infection and cancer. *Trends Immunol.* **38**, 542–557 (2017).

29. E. Moore, P. E. Clavijo, R. Davis, H. Cash, C. Van Waes, Y. Kim, C. Allen, Established T cell-inflamed tumors rejected after adaptive resistance was reversed by combination STING activation and PD-1 pathway blockade. *Cancer Immunol. Res.* **4**, 1061–1071 (2016).

30. J. Fu, D. B. Kanne, M. Leong, L. H. Glickman, S. M. McWhirter, E. Lemmens, K. Mechette, J. J. Leong, P. Lauer, W. Liu, K. E. Sivick, Q. Zeng, K. C. Soares, L. Zheng, D. A. Portnoy, J. J. Woodward, D. M. Pardoll, T. W. Dubensky, Y. Kim, STING agonist formulated cancer vaccines can cure established tumors resistant to PD-1 blockade. *Sci. Transl. Med.* **7**, 283ra252 (2015).

31. T. Sen, B. L. Rodriguez, L. Chen, C. M. D. Corte, N. Morikawa, J. Fujimoto, S. Cristea, T. Nguyen, L. Diao, L. Li, Y. Fan, Y. Yang, J. Wang, B. S. Glisson, I. I. Wistuba, J. Sage, J. V. Heymach, D. L. Gibbons, L. A. Byers, Targeting DNA damage response promotes antitumor immunity through STING-mediated T-cell activation in small cell lung cancer. *Cancer Discov.* **9**, 646–661 (2019).

32. T. Yang, D. Huang, C. Li, D. Zhao, J. Li, M. Zhang, Y. Chen, Q. Wang, Z. Liang, X.-J. Liang, Z. Li, Y. Huang, Rolling microneedle electrode array (RoMEA) empowered nucleic acid delivery and cancer immunotherapy. *Nano Today* **36**, 101017 (2021).

33. T. Chen, Q. Li, Z. Liu, Y. Chen, F. Feng, H. Sun, Peptide-based and small synthetic molecule inhibitors on PD-1/PD-L1 pathway: A new choice for immunotherapy? *Eur. J. Med. Chem.* **161**, 378–398 (2019).

34. Y. Guo, Q. Zhang, Q. Zhu, J. Gao, X. Zhu, H. Yu, Y. Li, C. Zhang, Copackaging photosensitizer and PD-L1 siRNA in a nucleic acid nanogel for synergistic cancer photoimmunotherapy. *Sci. Adv.* **8**, eabn2941 (2022).

35. O. Demaria, S. Cornen, M. Daëron, Y. Morel, R. Medzhitov, E. Vivier, Harnessing innate immunity in cancer therapy. *Nature* **574**, 45–56 (2019).

36. M. Zheng, Y. Liu, Y. Wang, D. Zhang, Y. Zou, W. Ruan, J. Yin, W. Tao, J. B. Park, B. Shi, ROS-responsive polymeric siRNA nanomedicine stabilized by triple interactions for the robust glioblastoma combinational RNAi therapy. *Adv. Mater.* **31**, e1903277 (2019).

37. S. J. Tan, P. Kiatwuthinon, Y. H. Roh, J. S. Kahn, D. Luo, Engineering nanocarriers for siRNA delivery. *Small* **7**, 841–856 (2011).

38. U. Scherf, A. Gutacker, N. Koenen, All-conjugated block copolymers. *Acc. Chem. Res.* **41**, 1086–1097 (2008).

39. J. Liu, H. Cabral, P. Mi, Nanocarriers address intracellular barriers for efficient drug delivery, overcoming drug resistance, subcellular targeting and controlled release. *Adv. Drug Deliv. Rev.* **207**, 115239 (2024).

40. P. Mi, K. Miyata, K. Kataoka, H. Cabral, Clinical translation of self-assembled cancer nanomedicines. *Adv. Ther.* **4**, 2000159 (2021).

41. H. Cabral, K. Miyata, K. Osada, K. Kataoka, Block copolymer micelles in nanomedicine applications. *Chem. Rev.* **118**, 6844–6892 (2018).

42. K. Miyata, R. J. Christie, K. Kataoka, Polymeric micelles for nano-scale drug delivery. *React. Funct. Polym.* **71**, 227–234 (2011).

43. D. Kim, H. Matsuoka, S. I. Yusa, Y. Saruwatari, Collapse behavior of polyion complex (PIC) micelles upon salt addition and reforming behavior by dialysis and its temperature responsivity. *Langmuir* **36**, 15485–15492 (2020).

44. P. Mi, H. Cabral, K. Kataoka, Ligand-installed nanocarriers toward precision therapy. *Adv. Mater.* **32**, e1902604 (2020).

45. P. Mi, Stimuli-responsive nanocarriers for drug delivery, tumor imaging, therapy and theranostics. *Theranostics* **10**, 4557–4588 (2020).

46. Z. Guo, H. He, Y. Zhang, J. Rao, T. Yang, T. Li, L. Wang, M. Shi, M. Wang, S. Qiu, X. Song, H. Ke, H. Chen, Heavy-atom-modulated supramolecular assembly increases antitumor potency against malignant breast tumors via tunable cooperativity. *Adv. Mater.* **33**, e2004225 (2021).

47. H. Chen, L. Xiao, Y. Anraku, P. Mi, X. Liu, H. Cabral, A. Inoue, T. Nomoto, A. Kishimura, N. Nishiyama, K. Kataoka, Polyion complex vesicles for photoinduced intracellular delivery of amphiphilic photosensitizer. *J. Am. Chem. Soc.* **136**, 157–163 (2014).

48. N. Nishiyama, A. Iriyama, W. D. Jang, K. Miyata, K. Itaka, Y. Inoue, H. Takahashi, Y. Yanagi, Y. Tamaki, H. Koyama, K. Kataoka, Light-induced gene transfer from packaged DNA enveloped in a dendrimeric photosensitizer. *Nat. Mater.* **4**, 934–941 (2005).

49. T. Nomoto, S. Fukushima, M. Kumagai, K. Machitani, Arnida, Y. Matsumoto, M. Oba, K. Miyata, K. Osada, N. Nishiyama, K. Kataoka, Three-layered polyplex micelle as a multifunctional nanocarrier platform for light-induced systemic gene transfer. *Nat. Commun.* **5**, 3545 (2014).

50. H. C. Yen, H. Cabral, P. Mi, K. Toh, Y. Matsumoto, X. Liu, H. Koori, A. Kim, K. Miyazaki, Y. Miura, N. Nishiyama, K. Kataoka, Light-induced cytosolic activation of reduction-sensitive camptothecin-loaded polymeric micelles for spatiotemporally controlled in vivo chemotherapy. *ACS Nano* **8**, 11591–11602 (2014).

51. A. S. Piotrowski-Daspit, A. C. Kauffman, L. G. Bracaglia, W. M. Saltzman, Polymeric vehicles for nucleic acid delivery. *Adv. Drug Deliv. Rev.* **156**, 119–132 (2020).

52. P. Mi, H. Cabral, D. Kokuryo, M. Rafi, Y. Terada, I. Aoki, T. Saga, I. Takehiko, N. Nishiyama, K. Kataoka, Gd-DTPA-loaded polymer-metal complex micelles with high relaxivity for MR cancer imaging. *Biomaterials* **34**, 492–500 (2013).

53. S. Takae, K. Miyata, M. Oba, T. Ishii, N. Nishiyama, K. Itaka, Y. Yamasaki, H. Koyama, K. Kataoka, PEG-detachable polyplex micelles based on disulfide-linked block catiomers as bioresponsive nonviral gene vectors. *J. Am. Chem. Soc.* **130**, 6001–6009 (2008).

54. Y. Oe, R. J. Christie, M. Naito, S. A. Low, S. Fukushima, K. Toh, Y. Miura, Y. Matsumoto, N. Nishiyama, K. Miyata, K. Kataoka, Actively-targeted polyion complex micelles stabilized by cholesterol and disulfide cross-linking for systemic delivery of siRNA to solid tumors. *Biomaterials* **35**, 7887–7895 (2014).

55. R. J. Christie, Y. Matsumoto, K. Miyata, T. Nomoto, S. Fukushima, K. Osada, J. Halnaut, F. Pittella, H. J. Kim, N. Nishiyama, K. Kataoka, Targeted polymeric micelles for siRNA treatment of experimental cancer by intravenous injection. *ACS Nano* **6**, 5174–5189 (2012).

56. K. Abstiens, M. Gregoritza, A. M. Goepferich, Ligand density and linker length are critical factors for multivalent nanoparticle-receptor interactions. *ACS Appl. Mater. Interfaces* **11,** 1311–1320 (2019).

57. K. Suzuki, Y. Miura, Y. Mochida, T. Miyazaki, K. Toh, Y. Anraku, V. Melo, X. Liu, T. Ishii, O. Nagano, H. Saya, H. Cabral, K. Kataoka, Glucose transporter 1-mediated vascular translocation of nanomedicines enhances accumulation and efficacy in solid tumors. *J. Control. Release* **301,** 28–41 (2019).

58. Y. Cui, J. Liu, L. Cui, C. Wei, M. Xu, Z. Wu, Y. Guo, P. Mi, Tumor-targeting and activatable biomimetic nanococktails synergistically regulate immune responses for spatiotemporal immunotherapy of low immunogenic solid tumors. *Nano Today* **57**, 102380 (2024).

59. X. Zheng, L. Wang, Q. Pei, S. He, S. Liu, Z. Xie, Metal-organic framework@porous organic polymer nanocomposite for photodynamic therapy. *Chem. Mater.* ***29***, 2374–2381 (2017).

60. A. Bukkuri, R. A. Gatenby, J. S. Brown, GLUT1 production in cancer cells: A tragedy of the commons. *NPJ. Syst. Biol. Appl.* **8**, 22 (2022).

61. Z. Zhang, X. Li, F. Yang, C. Chen, P. Liu, Y. Ren, P. Sun, Z. Wang, Y. You, Y. X. Zeng, X. Li, DHHC9-mediated GLUT1 S-palmitoylation promotes glioblastoma glycolysis and tumorigenesis. *Nat. Commun.* **12**, 5872 (2021).

62. M. Wu, H. Li, R. Liu, X. Gao, M. Zhang, P. Liu, Z. Fu, J. Yang, D. Zhang-Negrerie, Q. Gao, Galactose conjugated platinum (II) complex targeting the Warburg effect for treatment of non-small cell lung cancer and colon cancer. *Eur. J. Med. Chem.* **110**, 32–42 (2016).

63. Y. Zhou, F. Zhu, Y. Liu, M. Zheng, Y. Wang, D. Zhang, Y. Anraku, Y. Zou, J. Li, H. Wu, X. Pang, W. Tao, O. Shimoni, A. I. Bush, X. Xue, B. Shi, Blood-brain barrier-penetrating siRNA nanomedicine for Alzheimer's disease therapy. *Sci. Adv.* **6**, eabc7031 (2020).

64. Y. Yi, H. J. Kim, M. Zheng, P. Mi, M. Naito, B. S. Kim, H. S. Min, K. Hayashi, F. Perche, K. Toh, X. Liu, Y. Mochida, H. Kinoh, H. Cabral, K. Miyata, K. Kataoka, Glucose-linked sub-50-nm unimer polyion complex-assembled gold nanoparticles for targeted siRNA delivery to glucose transporter 1-overexpressing breast cancer stem-like cells. *J. Control. Release* **295**, 268–277 (2019).

65. C. Zhang, Z. Zeng, D. Cui, S. He, Y. Jiang, J. Li, J. Huang, K. Pu, Semiconducting polymer nano-PROTACs for activatable photo-immunometabolic cancer therapy. *Nat. Commun.* **12**, 2934 (2021).

66. A. Gao, B. Chen, J. Gao, F. Zhou, M. Saeed, B. Hou, Y. Li, H. Yu, Sheddable prodrug vesicles combating adaptive immune resistance for improved photodynamic immunotherapy of cancer. *Nano Lett.* **20**, 353–362 (2020).

67. D. Liu, B. Chen, Y. Mo, Z. Wang, T. Qi, Q. Zhang, Y. Wang, Redox-activated porphyrin-based liposome remote-loaded with indoleamine 2,3-dioxygenase (IDO) inhibitor for synergistic photoimmunotherapy through induction of immunogenic cell death and blockage of IDO pathway. *Nano Lett.* **19**, 6964–6976 (2019).

68. B. Hou, L. Zhou, H. Wang, M. Saeed, D. Wang, Z. Xu, Y. Li, H. Yu, Engineering stimuli-activatable boolean logic prodrug nanoparticles for combination cancer immunotherapy. *Adv. Mater.* **32**, e1907210 (2020).

69. L. P. Zhao, R. R. Zheng, J. Q. Huang, X. Y. Chen, F. A. Deng, Y. B. Liu, C. Y. Huang, X. Y. Yu, H. Cheng, S. Y. Li, Self-delivery photo-immune stimulators for photodynamic sensitized tumor immunotherapy. *ACS Nano* **14**, 17100–17113 (2020).

70. L. Wang-Bishop, M. Wehbe, D. Shae, J. James, B. C. Hacker, K. Garland, P. P. Chistov, M. Rafat, J. M. Balko, J. T. Wilson, Potent STING activation stimulates immunogenic cell death to enhance antitumor immunity in neuroblastoma. *J. Immunother. Cancer* **8**, e000282 (2020).

71. K. M. Garland, T. L. Sheehy, J. T. Wilson, Chemical and biomolecular strategies for STING pathway activation in cancer immunotherapy. *Chem. Rev.* **122**, 5977–6039 (2022).

72. A. K. Mehta, E. M. Cheney, C. A. Hartl, C. Pantelidou, M. Oliwa, J. A. Castrillon, J. R. Lin, K. E. Hurst, M. de Oliveira Taveira, N. T. Johnson, W. M. Oldham, M. Kalocsay, M. J. Berberich, S. A. Boswell, A. Kothari, S. Johnson, D. A. Dillon, M. Lipschitz, S. Rodig, S. Santagata, J. E. Garber, N. Tung, J. Yelamos, J. E. Thaxton, E. A. Mittendorf, P. K. Sorger, G. I. Shapiro, J. L. Guerriero, Targeting immunosuppressive macrophages overcomes PARP inhibitor resistance in BRCA1-associated triple-negative breast cancer. *Nat. Cancer* **2**, 66–82 (2021).

73. M. Arnold, E. Morgan, H. Rumgay, A. Mafra, D. Singh, M. Laversanne, J. Vignat, J. R. Gralow, F. Cardoso, S. Siesling, I. Soerjomataram, Current and future burden of breast cancer: Global statistics for 2020 and 2040. *Breast* **66**, 15–23 (2022).

74. V. Geldhof, L. P. M. H. de Rooij, L. Sokol, J. Amersfoort, M. De Schepper, K. Rohlenova, G. Hoste, A. Vanderstichele, A. M. Delsupehe, E. Isnaldi, N. Dai, F. Taverna, S. Khan, A.-C. K. Truong, L.-A. Teuwen, F. Richard, L. Treps, A. Smeets, I. Nevelsteen, B. Weynand, S. Vinckier, L. Schoonjans, J. Kalucka, C. Desmedt, P. Neven, M. Mazzone, G. Floris, K. Punie, M. Dewerchin, G. Eelen, H. Wildiers, X. Li, Y. Luo, P. Carmeliet, Single cell atlas identifies lipid-processing and immunomodulatory endothelial cells in healthy and malignant breast. *Nat. Commun.* **13**, 5511 (2022).

75. G. Kroemer, L. Senovilla, L. Galluzzi, F. Andre, L. Zitvogel, Natural and therapy-induced immunosurveillance in breast cancer. *Nat. Med.* **21**, 1128–1138 (2015).

76. L. Szablewski, Expression of glucose transporters in cancers. *Biochim. Biophys. Acta* **1835**, 164–169 (2013).

77. K. C. Carvalho, I. W. Cunha, R. M. Rocha, F. R. Ayala, M. M. Cajaíba, M. D. Begnami, R. S. Vilela, G. R. Paiva, R. G. Andrade, F. A. Soares, GLUT1 expression in malignant tumors and its use as an immunodiagnostic marker. *Clinics* **66**, 965–972 (2011).

78. N. Sasaki, M. Homme, S. Kitajima, Targeting the loss of cGAS/STING signaling in cancer. *Cancer Sci.* **114**, 3806–3815 (2023).

79. S. Kitajima, E. Ivanova, S. Guo, R. Yoshida, M. Campisi, S. K. Sundararaman, S. Tange, Y. Mitsuishi, T. C. Thai, S. Masuda, B. P. Piel, L. M. Sholl, P. T. Kirschmeier, C. P. Paweletz, H. Watanabe, M. Yajima, D. A. Barbie, Suppression of STING associated with LKB1 loss in KRAS-driven lung cancer. *Cancer Discov.* **9**, 34–45 (2019).

80. X. Guo, P. Tu, X. Wang, C. Du, W. Jiang, X. Qiu, J. Wang, L. Chen, Y. Chen, J. Ren, Decomposable nanoagonists enable NIR-elicited cGAS-STING activation for tandem-amplified photodynamic-metalloimmunotherapy. *Adv. Mater.* **36**, e2313029 (2024).

81. F. Ding, J. Liu, K. Ai, C. Xu, X. Mao, Z. Liu, H. Xiao, Simultaneous activation of pyroptosis and cGAS-STING pathway with epigenetic/photodynamic nanotheranostic for enhanced tumor photoimmunotherapy. *Adv. Mater.* **36**, e2306419 (2024).

82. Z. Mai, J. Zhong, J. Zhang, G. Chen, Y. Tang, W. Ma, G. Li, Z. Feng, F. Li, X. J. Liang, Y. Yang, Z. Yu, Carrier-free immunotherapeutic nano-booster with dual synergistic effects based on glutaminase inhibition combined with photodynamic therapy. *ACS Nano* **17**, 1583–1596 (2023).

83. X. Tang, Q. Sheng, C. Xu, M. Li, J. Rao, X. Wang, Y. Long, Y. Tao, X. He, Z. Zhang, Q. He, pH/ATP cascade-responsive nano-courier with efficient tumor targeting and siRNA unloading for photothermal-immunotherapy. *Nano Today* **37**, 101083 (2021).

84. Q. Liu, J. Wang, S. Li, G. Li, Q. Chen, Z. Hong, Folate-targeted polyethylene glycol-modified photosensitizers for photodynamic therapy. *J. Pharm. Sci.* **108**, 2102–2111 (2019).

85. Y. Chen, M. Sajjad, Y. Wang, C. Batt, H. A. Nabi, R. K. Pandey, TSPO 18 kDa (PBR) targeted photosensitizers for cancer imaging (PET) and PDT. *ACS Med. Chem. Lett.* **2**, 136–141 (2011).

86. X. Zhao, C. X. Yang, L. G. Chen, X. P. Yan, Dual-stimuli responsive and reversibly activatable theranostic nanoprobe for precision tumor-targeting and fluorescence-guided photothermal therapy. *Nat. Commun.* **8**, 14998 (2017).
